# Comorbid and co-occurring conditions in migraine and associated risk of increasing headache pain intensity and headache frequency: results of the migraine in America symptoms and treatment (MAST) study

**DOI:** 10.1186/s10194-020-1084-y

**Published:** 2020-03-02

**Authors:** Dawn C. Buse, Michael L. Reed, Kristina M. Fanning, Ryan Bostic, David W. Dodick, Todd J. Schwedt, Sagar Munjal, Preeti Singh, Richard B. Lipton

**Affiliations:** 1 Albert Einstein College of Medicine, 1250 Waters Place, 8th Floor, Bronx, NY 10461 USA; 2 Vedanta Research, 23 Tanyard Court, Chapel Hill, NC 27517 USA; 3 Mayo Clinic, 5777 E Mayo Blvd, Phoenix, AZ 85054 USA; 4 Promius Pharma, 107 College Road East, Princeton, NJ 08534 USA; 5 Montefiore Medical Center, 1165 Morris Park Avenue, Rousso Building, Room 332, Bronx, NY 10461 USA

**Keywords:** Epidemiology, Migraine, Comorbidities, Sociodemographics, Headache pain intensity, Headache frequency

## Abstract

**Background:**

Migraine has many presumed comorbidities which have rarely been compared between samples with and without migraine. Examining the association between headache pain intensity and monthly headache day (MHD) frequency with migraine comorbidities is novel and adds to our understanding of migraine comorbidity.

**Methods:**

The MAST Study is a prospective, web-based survey that identified US population samples of persons with migraine (using modified *International Classification of Headache Disorders*-3 beta criteria) and without migraine. Eligible migraine participants averaged ≥1 MHDs over the prior 3 months. Comorbidities “confirmed by a healthcare professional diagnosis” were endorsed by respondents from a list of 21 common cardiovascular, neurologic, psychiatric, sleep, respiratory, dermatologic, pain and medical comorbidities. Multivariable binary logistic regression calculated odds ratios (OR) and 95% confidence intervals for each condition between the two groups adjusting for sociodemographics. Modeling within the migraine cohort assessed rates of conditions as a function of headache pain intensity, MHD frequency, and their combination.

**Results:**

Analyses included 15,133 people with migraine (73.0% women, 77.7% White, mean age 43 years) and 77,453 controls (46.4% women, 76.8% White, mean age 52 years). People with migraine were significantly (*P* < 0.001) more likely to report insomnia (OR 3.79 [3.6, 4.0]), depression (OR 3.18 [3.0, 3.3]), anxiety (OR 3.18 [3.0 3.3]), gastric ulcers/GI bleeding (OR 3.11 [2.8, 3.5]), angina (OR 2.64 [2.4, 3.0]) and epilepsy (OR 2.33 [2.0, 2.8]), among other conditions. Increasing headache pain intensity was associated with comorbidities related to inflammation (psoriasis, allergy), psychiatric disorders (depression, anxiety) and sleep conditions (insomnia). Increasing MHD frequency was associated with increased risk for nearly all conditions and most prominent among those with comorbid gastric ulcers/GI bleeding, diabetes, anxiety, depression, insomnia, asthma and allergies/hay fever.

**Conclusions:**

In regression models controlled for sociodemographic variables, all conditions studied were reported more often by those with migraine. Whether entered into the models separately or together, headache pain intensity and MHD frequency were associated with increased risk for many conditions. Future work is required to understand the causal sequence of relationships (direct causality, reverse causality, shared underlying predisposition), the potential confounding role of healthcare professional consultation and treatment, and potential detection bias.

## Background

Migraine is a common and often disabling disease. The 2016 Global Burden of Disease data revealed that migraine was the second most disabling condition worldwide, second only low back pain [1]. Migraine is often divided into episodic migraine (EM) or chronic migraine (CM) based largely on the number of monthly headache days (MHDs).

Comorbidities and co-occurring conditions (hereafter referred to as comorbidities) contribute to the overall burden of migraine. Diseases are said to be co-occurring if the same person has more than one disease. Comorbidity is the greater than chance association between the two conditions in the same individual [2]. There are many comorbidities associated with migraine including cardiovascular disorders (i.e., stroke, myocardial infarction [3–10], psychiatric disorders (i.e., depression, anxiety, panic disorder, bipolar disorder, personality disorders, suicide attempts) [11–21], neurologic diseases (i.e., epilepsy), sleep conditions (i.e., insomnia, restless leg syndrome, sleep apnea, poor sleep quality and duration) [22–24], inflammatory conditions (i.e., allergic rhinitis, asthma) [25, 26] as well as chronic pain conditions (i.e., fibromyalgia) [27], among many others [28]. Many comorbidities have been identified as risk factors for progression to chronic migraine [29] and recent work has shown that the combination of comorbidities or “multimorbidity” is associated with medication overuse and new onset CM [30, 31].

Understanding specific comorbidities that occur with migraine are important for several reasons [32]. The identification of migraine comorbidities may help recognize common or overlapping underlying genetic and biologic mechanisms of diseases which may facilitate the development of new treatments targeted to subgroups [33]. Data from the Chronic Migraine Epidemiology and Outcomes (CaMEO) Study confirmed that certain comorbid diseases occur more frequently among those with migraine and, using latent class modeling, the study group identified 8 natural subgroups of migraine that exhibited distinct headache features [34]. Additional analysis of the same data set found that membership in some empirically defined comorbidity subgroups was associated with an increased risk of progression to chronic migraine [31]. In addition to disease progression, comorbid health problems may complicate the diagnosis of migraine as well as limit treatment options, as in the case with cardiovascular disease and the use of triptan medications. Comorbid and co-occurring health problems also contribute to disease burden for persons with migraine and may lower health related quality of life [17] and add to their economic burden [35].

Several studies have examined rates of single comorbidities comparing people with migraine and those without [3–19, 28]. Data from a primary care database study conducted in Scotland found that patients with *chronic* migraine (defined differently from International Committee of Headache Disorders (ICHD) criteria as patients had ≥4 prescriptions for migraine in the past 12 months) were more likely to experience a comorbid condition compared to control patients [36]. Prior work has also found that rates of some comorbid health problems increased with headache day frequency among people with migraine. This effect was seen in comparisons of people with EM and CM and when stratifying by low, moderate and high MHD frequency in those with EM [26, 30, 31, 37–41].

To the best of our knowledge, no study to date has assessed a large panel of already known or likely comorbidities in a representative sample of persons with migraine and migraine-free controls. This is also true for an evaluation on the impact of headache pain intensity, and the combination of MHD frequency and headache pain intensity on the rate of occurrence for comorbid conditions.

The Migraine in America Symptoms and Treatment (MAST) Study was undertaken to evaluate patterns of migraine consultation, diagnosis, treatment and comorbid health burden among a representative non-clinical sample of people with migraine with an average of at least one headache day per month. Data on comorbid health problems from a non-migraine control sample were also obtained. Within the migraine cohort, we evaluated the impact of headache pain intensity, MHD frequency, and the combined effect of these variables on the rates of occurrence for comorbid health conditions. Our intent with this analysis was to compare rates of several conditions believed to be comorbidities of migraine including cardiovascular, neurologic, psychiatric, sleep, respiratory, dermatologic, pain, and general medical conditions among a large representative sample of people with active migraine and people without migraine. Additionally, we aimed to lay the groundwork for more detailed study of specific conditions by providing an overview of population comorbidity rates in persons with active migraine as well as to examine their association with sociodemographics and key headache characteristics - headache pain intensity and headache frequency.

## Methods

### Study design

A complete description of the MAST Study design and baseline sociodemographics has been published and will be briefly reviewed [42]. The MAST Study is a prospective, longitudinal cross-sectional web-based survey among United States (US) adults with and without migraine. At baseline, our goal was to identify a US sample that matched 2015 US Census data for age, gender and other demographic characteristics. Sampling fractions were adjusted as necessary during the recruiting phase to improve the representativeness of the sample where possible. Follow-up assessments were completed at 6 and 12 months. The current analysis utilizes the baseline respondent data set that was collected from October 2016 through January 2017.

### Participants

Members of an internet research panel (Research Now, Plano, TX), constructed to be demographically representative of the US population, were invited via email to participate in an online survey about health. An initial screening survey asked respondents about demographics and comorbid health conditions. Those participants who endorsed headache or migraine were asked to complete the American Migraine Study (AMS)/ American Migraine Prevalence and Prevention Study (AMPP) diagnostic module [43, 44], a validated tool which assesses self-reported migraine criteria using modified diagnostic criteria for migraine, taken from the *ICHD*-2 migraine criteria [45–47]. The criteria were considered modified because criterion A (at least 5 lifetime migraine events) and criterion B (duration of attack untreated from 4 to 72 h) were not confirmed. While the module was validated on *ICHD-2* criteria [48], there were no major changes in migraine criteria between the *ICHD-2* and the current *ICHD-3* criteria [45]. Respondents meeting the symptom criteria and headache frequency criteria (3 or more headache days in the prior 3 months and at least 1 headache in the past 30 days) were included in the migraine cohort. Respondents who did not meet migraine symptom criteria were classified as non-migraine control subjects, and those with inactive or low frequency headache were excluded from the analysis.

The MAST Study and respondent consent forms were reviewed by the Ethical and Independent Review Services (Independence, MO, USA) which granted an exemption from federal regulation 45 CFR 46.101(b)(2). All survey respondents provided informed consent.

### Assessments

The sociodemographic characteristics captured for both migraine and non-migraine cases included self-reported gender, age, marital status, Hispanic ethnicity, race, employment status, and total annual household income. Comorbid and co-occurring conditions were assessed by asking respondents “Do you have, or have you ever had, any of the following health conditions?”. Pre-coded response options included: allergies/hay fever, angina/heart disease (chest pain with exertion), arthritis – type unknown, osteoarthritis, rheumatoid arthritis, asthma, peripheral artery disease, anxiety, depression, diabetes, epilepsy, gastric ulcer or gastrointestinal (GI) bleeding, myocardial infarction (heart attack), high cholesterol, hypertension (high blood pressure), insomnia, kidney disease, psoriasis, rosacea, stroke/transient ischemic attack (TIA) and vitamin D deficiency. If respondents answered “yes”, they were then asked, “Has this condition been diagnosed or confirmed by a physician or other healthcare provider?” Conditions with a self-reported “yes” to healthcare provider diagnosis were included in the analysis.

MHDs were determined by asking respondents, “In the last 3 months (past 90 days), on how many days did you have a headache of any type or intensity? If a headache lasted more than 1 day, count each day.” This past 3 month estimate of the number of headaches was then divided by 3 to calculate MHD frequency. The migraine cohort was stratified based on MHDs into 5 groups: 1–4, 5–9, 10–14, 15–20 and ≥ 21 MHDs. Headache pain intensity was determined by asking, “On a scale of 0 to 10 (where 0 =no pain at all and 10 = pain as bad as it can be), on average how painful are your headaches?” Ratings of 0 to 3 were considered mild pain, ratings of 4 to 6 were considered moderate pain, and ratings of 7 to 10 were considered severe pain.

### Statistical analyses

Data cleaning and quality control measures were implemented to identify respondents with unreliable and/or inconsistent data. Analyses were performed using IBM SPSS Statistics Version 24.0 (IBM, Armonk NY). The goal of this analysis was to identify potential differences in the occurrence of comorbid or associated health problems between those with migraine and non-migraine controls. Descriptive data were generated for sociodemographics and each of the comorbid health conditions. Chi-square (for percentages) and t-tests for independent samples (for means) were used to compare the migraine and non-migraine cohorts. Multivariable binary logistic regression models were performed to assess differences in the likelihood of each comorbid condition as a function of the presence of a positive migraine screen, adjusting for sociodemographics (gender, age category, Hispanic ethnicity, race, marital status, employment status, and household income). Odds ratios (OR) and 95% confidence intervals (CI) are shown along with *P*-values.

Additional binary logistic regression modeling was implemented among the migraine cohort to assess first, the association of MHD frequency with each comorbid or associated health problem, second the association with headache pain intensity, and third to assess the combined effect of MHD frequency and headache pain intensity. MHD was assessed using frequency categories 1–4, 5–9, 10–14, 15–20 and ≥ 21 (reference = 1 to 4 MHDs). The headache pain intensity variable was categorized as low, moderate and severe (reference = low pain intensity). Each model included adjustments for sociodemographics (gender, age category, Hispanic ethnicity, race, marital status, employment status, and household income) and *P* ≤ 0.05 identified significant effects. Missing data were minimal and imputation procedures were not implemented.

## Results

### Sampling and Sociodemographics

A total of 117,150 people accepted the invitation to participate in the study. At the time of consent, 5,710 declined to participate and 18,854 failed to complete the survey, provided unreliable data, or otherwise did not meet inclusion criteria. This yielded 15,133 respondents in the migraine sample who met the symptom and minimal MHD frequency inclusion criteria, and 77,453 respondents in the non-migraine sample (Additional file 1). Demographic characteristics for the total sample of respondents after reliability checks were similar to US demography (when compared to the 2015 US Census Data [49, 50] (50.7% women vs. 51.6% census, 76.9% White vs. 78.4% census, 7.8% Hispanic origin vs. 15.5% census, 59.4% married vs. 52.4% census). Similarly, demographic characteristics and headache features for the migraine respondents were similar to the benchmark AMPP Study (90.4% EM vs. 91.9% in AMPP, 27.2% men with EM vs 21.0% in AMPP, 72.8% women with EM vs. 79.0% in AMPP, 38.8% grade III/IV Migraine Disability Assessment [MIDAS] scale vs. 31.5% in AMPP, 2.67 mean monthly attack frequency for men vs 2.33 in AMPP, 3.33 mean monthly attack frequency for women vs. 2.67 in AMPP) [42, 51]. MAST Study respondents with migraine reported slightly more disability and monthly attacks vs. AMPP Study respondents with migraine due to the MAST Study headache frequency inclusion criteria.

Baseline demographics are described in Table 1 for the migraine (*n* = 15,133) and non-migraine (*n* = 77,453) cohorts, and the overall analysis sample (*N* = 92,586). The mean age (SD) of the overall sample was 51 (15.8) years, with younger respondents represented in the migraine group (43 [13.6] years) versus the non-migraine group (52 [15.7] years, *P* < 0.001). The migraine vs. non-migraine group had a higher proportion of respondents aged 18–54 and a lower proportion of those aged 55 and older (*P* < 0.001). The migraine group also had a greater portion of women compared to the non-migraine group (73% vs. 46.4%, *P* < 0.001).

**Table 1:** Baseline demographics for the migraine (*n* = 15,133), non-migraine (*n* = 77,453) and total (*N* = 92,586) sample

	Migraine Sample (n = 15,133)	Non-migraine Sample (n = 77,453)	Total (N = 92,586)	Chi/ T test	*P*-value
Mean age, years (SD)	43 (13.6)	52 (15.7)	51 (15.8)	T = 67.900	< 0.001
Age, n (%)				4435.83	< 0.001
18–24 years	1147 (7.6)	2549 (3.3)	3696 (4.0)		
25–34 years	3674 (24.3)	10,933 (14.1)	14,607 (15.8)		
35–44 years	3648 (24.1)	11,771 (15.2)	15,419 (16.7)		
45–54 years	3386 (22.4)	14,929 (19.3)	18,315 (19.8)		
55–64 years	2091 (13.8)	15,414 (19.9)	17,505 (18.9)		
≥ 65 years	1187 (7.8)	21,855 (28.2)	23,042 (24.9)		
Women, n (%)	11,049 (73.0)	35,910 (46.4)	46,959 (50.7)	3596.916	< 0.001
White, n (%)	11,755 (77.7)	59,479 (76.8)	71,234 (76.9)	5.579	0.018
Hispanic Origin, n (%)	1546 (10.3)	5632 (7.4)	7178 (7.8)	148.616	< 0.001
Married, n (%)	8155 (53.9)	46,804 (60.4)	54,959 (59.4)	224.457	< 0.001
Employed, n (%)	10,903 (72.0)	47,916 (61.9)	58,819 (63.5)	566.591	< 0.001
Household Income, n (%)				537.545	< 0.001
< $25,000	1805 (12.3)	6518 (8.8)	8323 (9.4)		
$25,000–$49,000	3202 (21.8)	13,128 (17.8)	16,330 (18.4)		
$50,000–$74,999	3217 (21.9)	15,071 (20.4)	18,288 (20.6)		
$75,000–$99,999	2571 (17.5)	13,426 (18.2)	15,997 (18.0)		
≥ $100,000	3901 (26.5)	25,811 (34.9)	29,712 (33.5)		

With regard to race, ethnicity and marital status, 77.7% of those with migraine and 76.8% of those without migraine were White (*P* = 0.018), 10.3% in the migraine group and 7.4% in the non-migraine group were Hispanic (*P* < 0.001), and the migraine group was less likely to be married (53.9% vs. 60.4%, *P* < 0.001). The migraine group was more likely to be employed (72.0% vs. 61.9%, *P* < 0.001). However, the income distribution for those with migraine trended toward the lower income categories (*P* < 0.001). Baseline demographics were also analyzed by headache pain intensity ratings and MHD frequency, and results were similar to the overall population. (Additional files 2 and 3).

### Migraine versus non-migraine controls

**Fig. 1:**
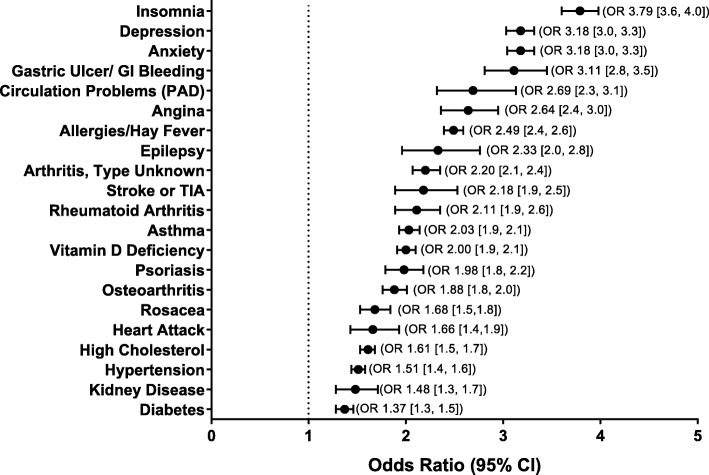
Relative odds of migraine (and 95% CI) vs. migraine free controls for each comorbid condition*. *Adjusted for sociodemographic characteristics (age, gender, Hispanic origin, race, marital status, employment, household income). Reference group is the non-migraine cohort. GI = gastrointestinal; OR = odds ratio; TIA = transient ischemic attack

Figure 1 provides the odds ratio and 95% CI for the migraine vs. non-migraine cohorts for each health condition, adjusting for sociodemographics. For all conditions except myocardial infarction, high cholesterol, hypertension, kidney disease and diabetes, the unadjusted percentages and the odds ratios were in the same direction, showing greater risk for the migraine cohort (Additional file 4). For these five conditions, the unadjusted rate of occurrence was higher in the non-migraine cohort, but after controlling for sociodemographic characteristics the migraine cohort demonstrated statistically greater odds of reporting each condition. Respondent age (not unexpectedly) was a main contributor to this shift in overall risk for those with migraine. After adjusting for sociodemographic variables, persons with migraine compared to the non-migraine cohort were greater than three times more likely to experience insomnia (OR 3.79 [3.6, 4.0]), depression (OR 3.18 [3.0, 3.3]), anxiety (OR 3.18 [3.0, 3.3]) and gastric ulcer/GI bleeding (OR 3.11 [2.8, 3.5]). The migraine group was at least twice as likely to experience peripheral artery disease (OR 2.69 [2.3, 3.1]), angina (OR 2.64 [2.4, 3.0]), allergies/hay fever (OR 2.49 [2.4, 2.6]), epilepsy (OR 2.33 [2.0, 2.8]), arthritis (type unknown) (OR 2.20 [2.1, 2.4]), stroke or TIA (OR 2.18 [1.9, 2.5]), rheumatoid arthritis (OR 2.11 [1.9, 2.4]), asthma (OR 2.03 [1.9, 2.1]), and vitamin D deficiency (OR 2.00 [1.9, 2.1]). Figure 1 provides the adjusted odds ratios for the remaining comorbid or co-occurring conditions included in this analysis (psoriasis, osteoarthritis, rosacea, myocardial infarction, high cholesterol, hypertension, kidney disease, and diabetes) and Additional file 7A to 7 U provides full modeling details.

### The impact of headache pain intensity and MHD frequency in the migraine cohort

For modeling *within* the migraine cohort, we evaluated the odds of each potentially comorbid condition in relation to headache pain intensity (Additional file 5) and to MHD frequency (Additional file 6) or the combined effect of both MHD and headache pain intensity. Modeling results are summarized here for the most conservative combined MHD and headache pain intensity models. Additional files 7A-7U  provide complete modeling details for each condition. Future analyses will look more closely at the relationship between migraine and individual comorbid health problems and evaluate their potential impact on patient reported outcomes.

#### Cardiovascular conditions

**Fig. 2:**
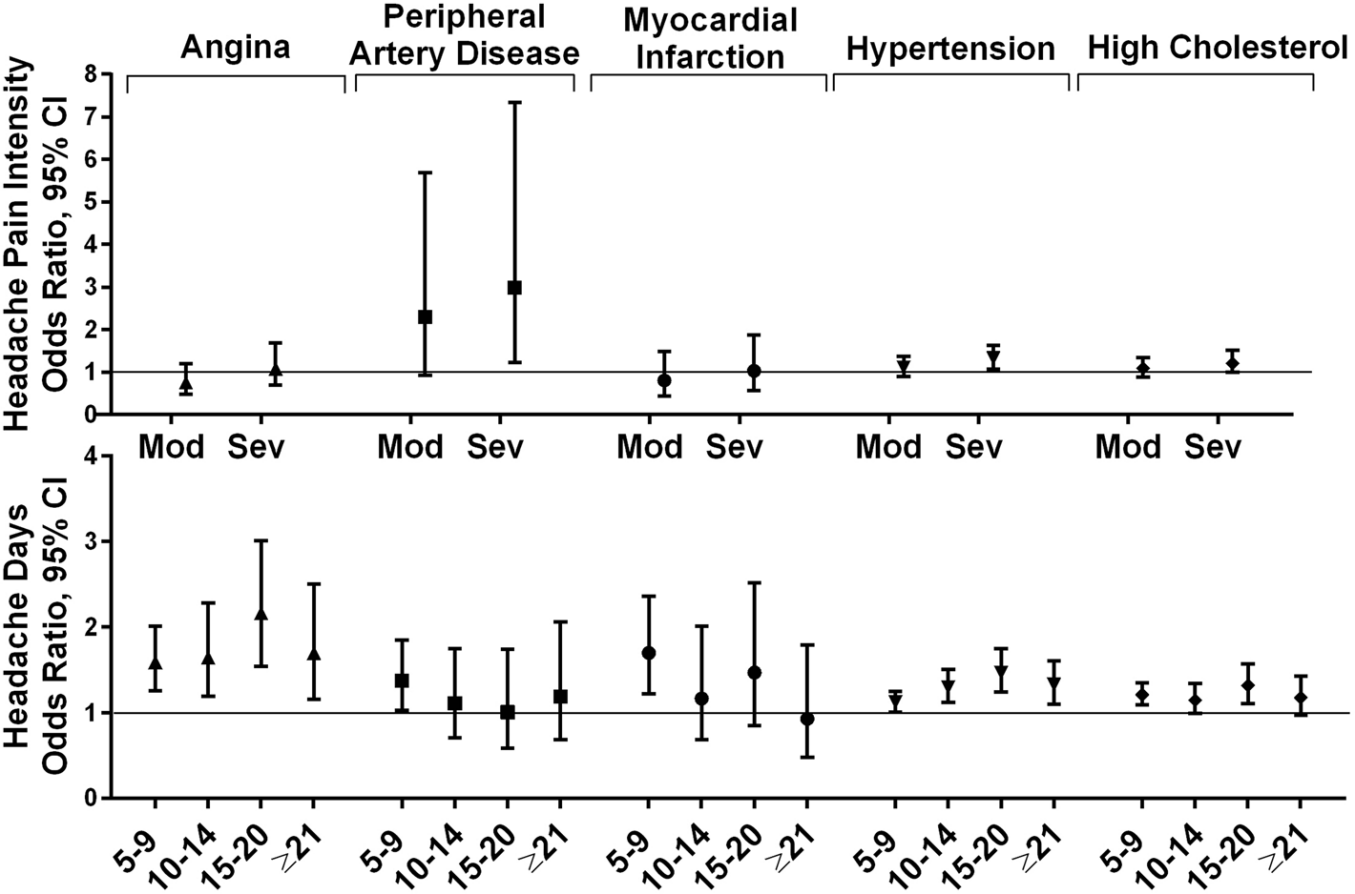
Among persons with migraine the odds ratios (and 95% CI) for each comorbid cardiovascular condition*. *Fully adjusted models with sociodemographic characteristics (age, gender, Hispanic origin, race, marital status, employment, household income), headache pain intensity and headache frequency. Reference groups are low headache pain intensity for headache pain intensity and low frequency headache (1–4 MHDs) for headache frequency

The cardiovascular comorbidities (Fig. 2) included in this analysis were angina, peripheral artery disease, myocardial infarction, hypertension, and high cholesterol. The risk of co-morbid angina increased with age and was more common among men, occurred less often among employed respondents, and as household incomes tended to increase. In comparison with the group with 1–4 MHDs, the odds of angina were greater in all groups with ≥5 MHDs. The risk of angina was not related to headache pain intensity (Additional file 7A). Results for peripheral artery disease followed a similar pattern for age and gender but only the 5–9 MHD category compared to 1–4 days was associated with a statistically significant increased risk for this condition. Severe headache pain intensity was associated with nearly 3 times the odds of peripheral artery disease (OR 2.99 [1.22, 7.35]) (Additional file 7B). Myocardial infarction modeling results followed the same pattern for sociodemographics and additional risk was only associated with the 5–9 MHD category and not at all for headache pain intensity ratings (Additional file 7C). The findings for hypertension and high cholesterol followed same pattern for age, gender, employment status and household income, and in addition, being married was associated with increased odds of hypertension (OR 1.12 [1.02, 1.22]) and high cholesterol (OR 1.12 [1.02, 1.23]). Increasing MHD frequency and severe headache pain intensity were both associated with increased risk in the individual models and in the combined MHD and headache pain intensity models (Additional file 7D and 7E).

#### Neurologic conditions

**Fig. 3:**
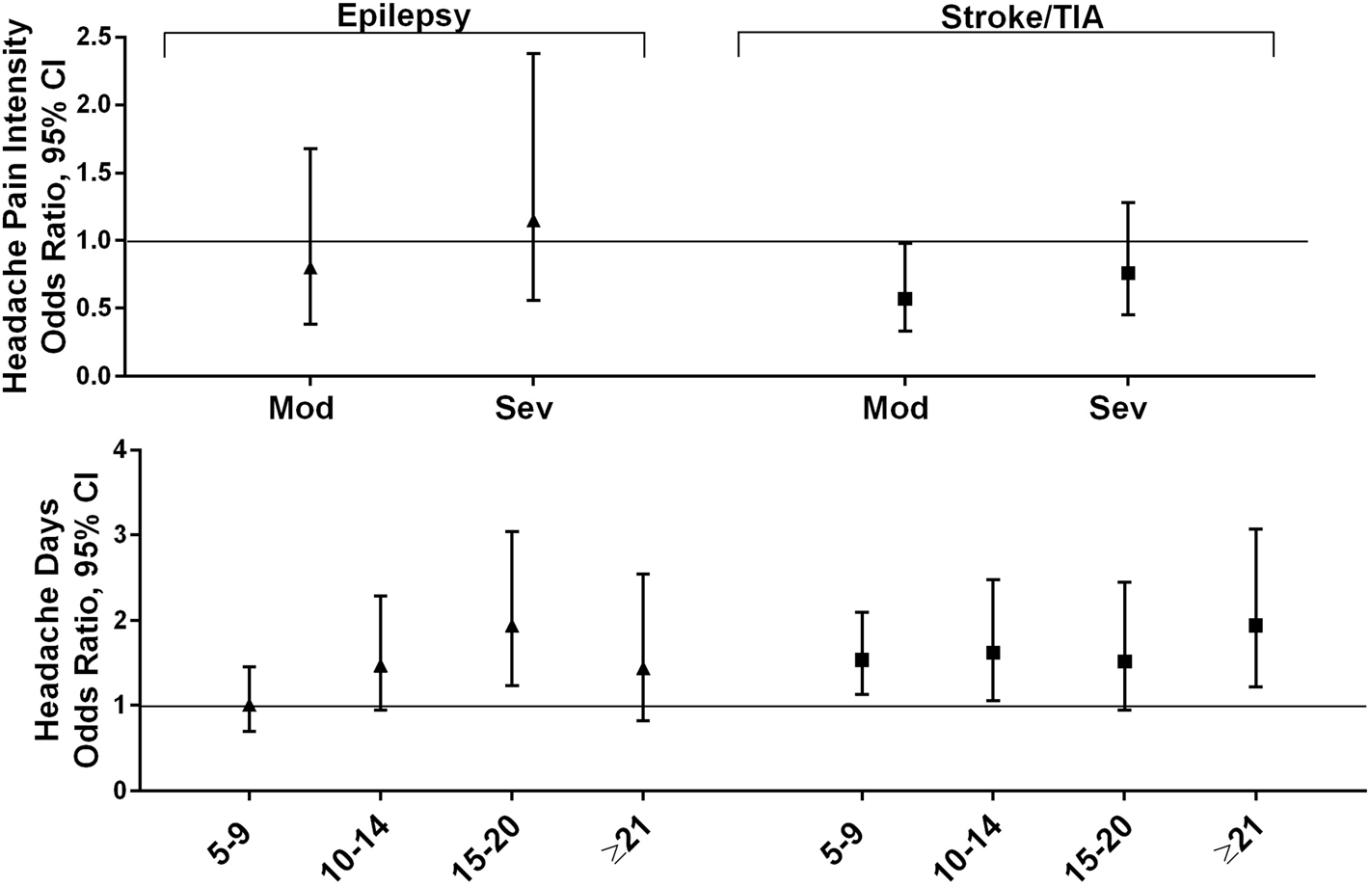
Among persons with migraine the odds ratios (and 95% CI) for each comorbid neurologic condition*. *Fully adjusted models with sociodemographic characteristics (age, gender, Hispanic origin, race, marital status, employment, household income), headache pain intensity and headache frequency. Reference groups are low headache pain intensity for headache pain intensity and low frequency headache (1–4 MHDs) for headache frequency

The neurologic conditions in this analysis were epilepsy and stroke or TIA (Fig. 3). Being employed and increasing household income were associated with lower odds of epilepsy, but headache pain intensity rating and MHD frequency were generally not associated with co-occurrence of epilepsy (Additional file 7F); the exception is that the odds of epilepsy given migraine were increased for the 15–20 day MHD group (OR 1.94 [1.23, 3.04]). The odds of reporting stroke increased with age (especially for those age ≥ 65, OR 14.41[4.39, 47.27]) and in men (OR 1.73 [1.34, 2.24]). Employment and increasing income were associated with decreased odds. Increasing MHD frequency was associated with increased risk for stroke or TIA and only moderate headache pain intensity (versus low headache pain intensity) was associated with decreased stroke risk (Additional file 7G).

#### General medical conditions

**Fig. 4:**
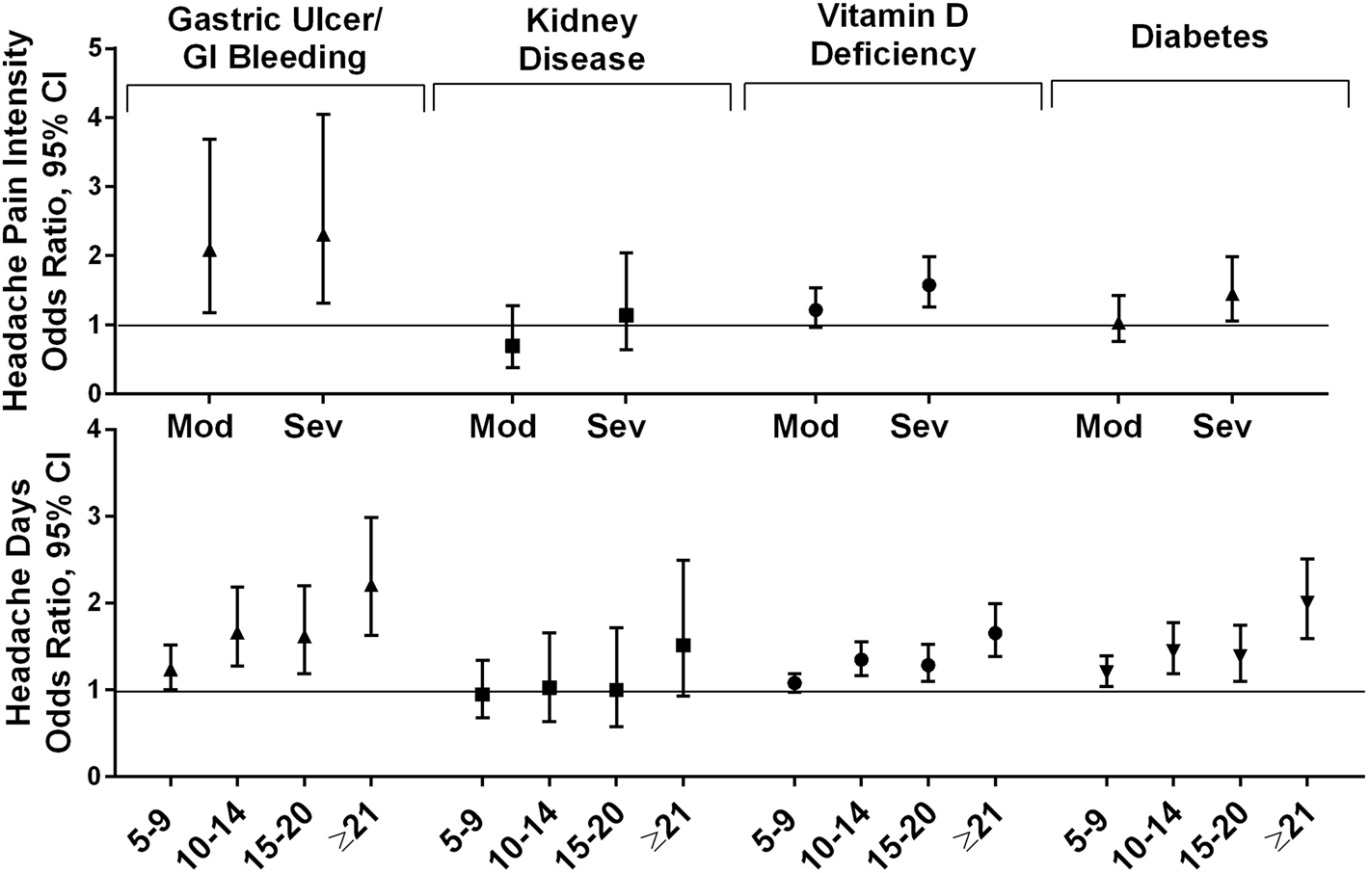
Among persons with migraine the odds ratios (and 95% CI) for each comorbid general medical condition*. Fully adjusted models with sociodemographic characteristics (age, gender, Hispanic origin, race, marital status, employment, household income), headache pain intensity and headache frequency. Reference groups are low headache pain intensity for headache pain intensity and low frequency headache (1–4 MHDs) for headache frequency

The general medical conditions in this analysis included gastric ulcer/GI bleeding, kidney disease, vitamin D deficiency, and diabetes (Fig. 4). For those with gastric ulcer/GI bleeding, respondent age and being male were associated with increased odds, as were both increasing MHD frequency and moderate and severe headache pain intensity (Additional file 7H). For kidney disease, increasing age and being male were associated with increased risk, and being employed was associated with less risk, but no association with MHD frequency or headache pain intensity ratings was observed (Additional file 7I). For vitamin D deficiency, older age was associated with increased risk, but the risk was reduced among men, non-Hispanics, Whites, and those married and employed. Both MHD frequency and headache pain intensity rating increases were associated with increased risk (Additional file 7J). Rates of diabetes were associated with increasing age, being male, and being married as well as increasing MHD frequency category and severe headache pain intensity. Decreased risk of diabetes was associated with being non-Hispanic, being employed, and with increasing income (Additional file 7K).

#### Psychiatric and sleep conditions

**Fig. 5:**
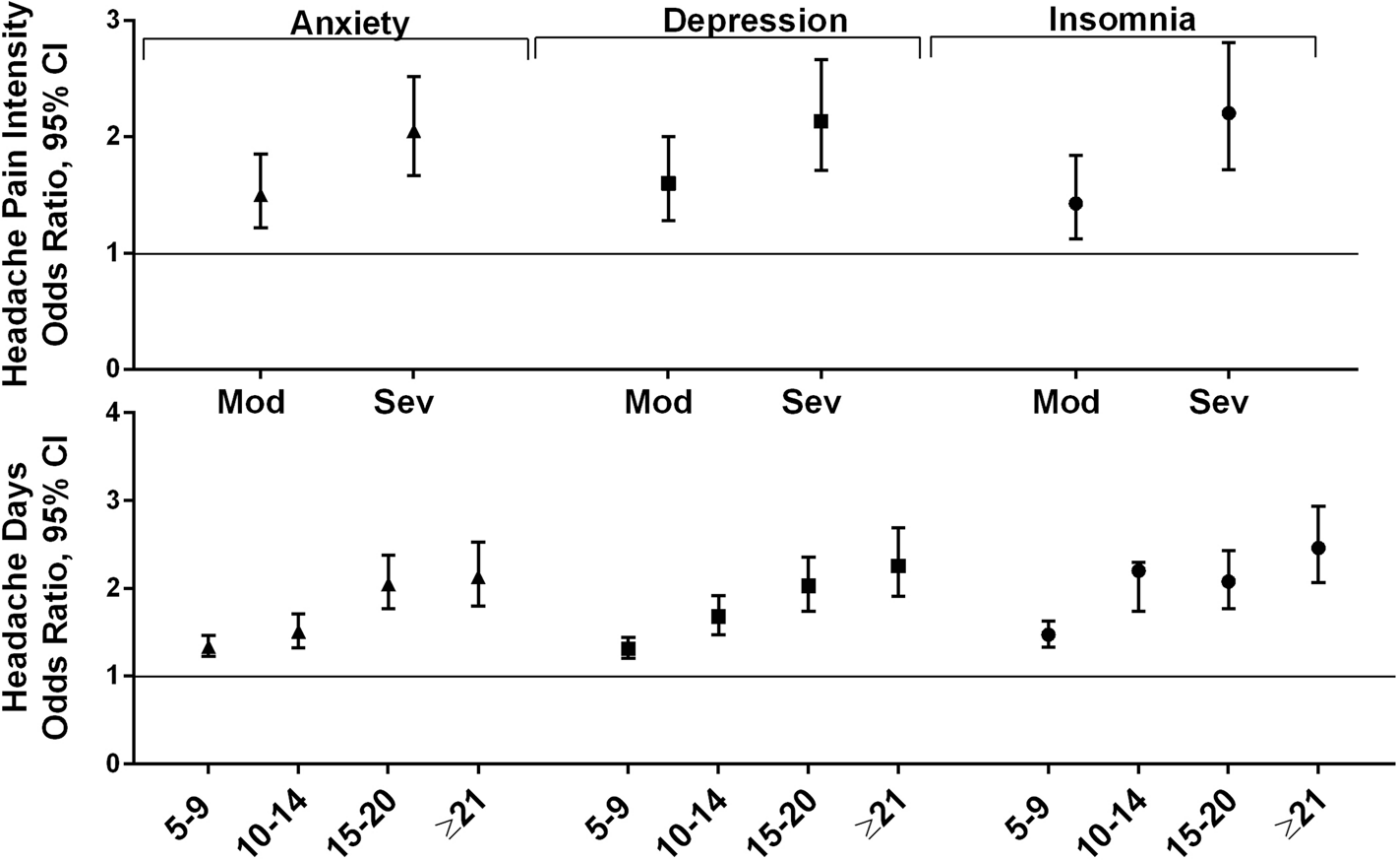
Among persons with migraine the odds ratios (and 95% CI) for comorbid psychiatric and sleep conditions*. Fully adjusted models with sociodemographic, characteristics (age, gender, Hispanic origin, race, marital status, employment, household income), headache pain intensity and headache frequency. Reference groups are low headache pain intensity for headache pain intensity and low frequency headache (1–4 MHDs) for headache frequency

The psychiatric and sleep conditions included in this analysis were anxiety, depression and insomnia **(**Fig. 5). The odds of anxiety increased among the lower age groups (age 23–34, 35–44, 45–54) and were lower for those in the ≥65 age group. Being a man, being married, being employed, and increasing household income were associated with lowered risk of anxiety, while being White, increasing MHD frequency and moderate and severe pain were associated with increased risk of anxiety (Additional file 7L). The odds of comorbid depression were lowered with increasing age, being married, being employed, and with increasing household income. Being White, MHD frequency and both moderate and severe pain were associated with increased odds of depression (Additional file 7M). The odds of insomnia increased with increasing age, increasing MHD frequency category, and with both moderate and severe headache pain intensity, and were lower among those who were married, employed, and with increasing income (Additional file 7N).

#### Respiratory and dermatologic conditions

**Fig. 6:**
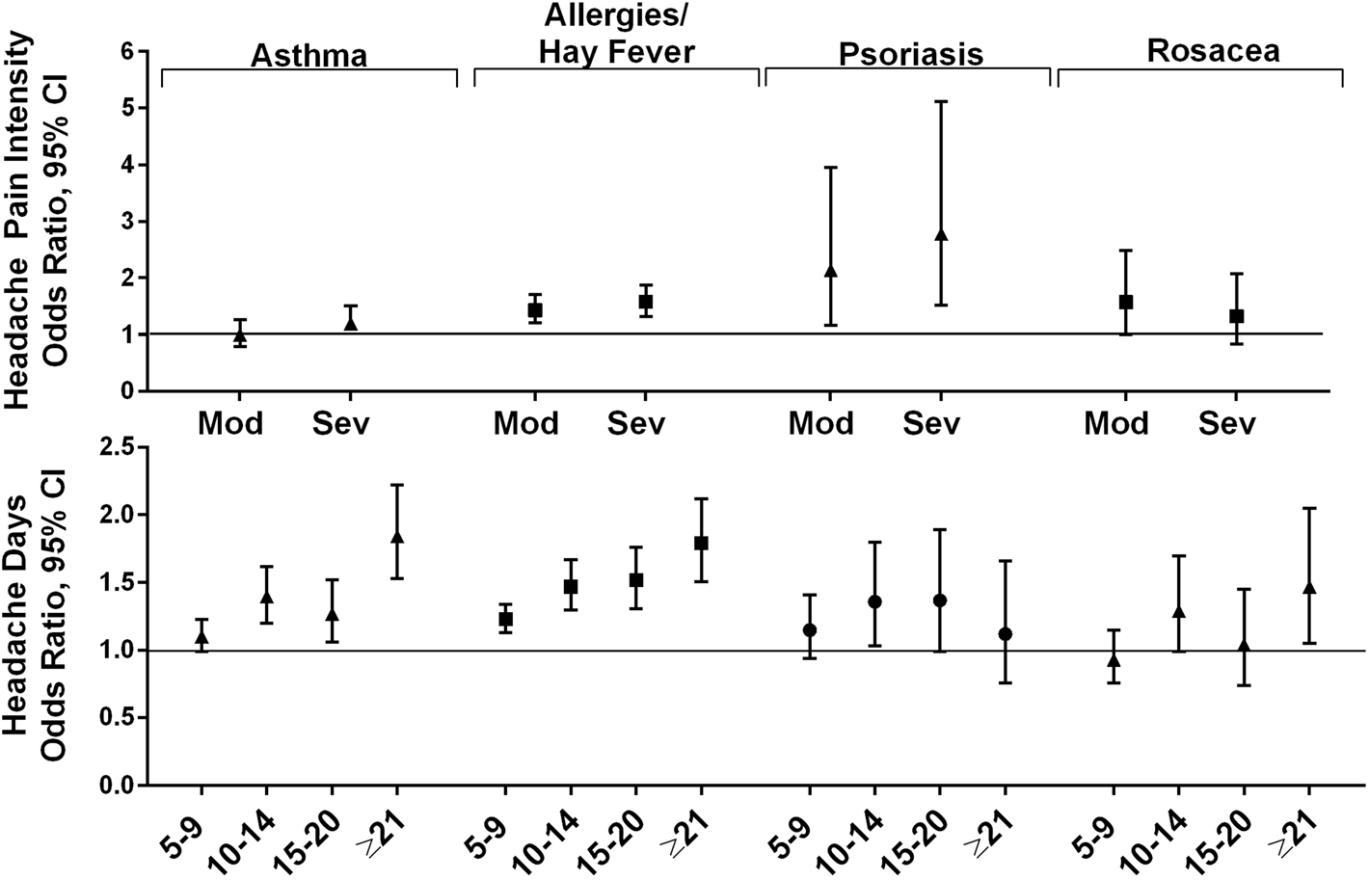
Among persons with migraine the odds ratios (and 95% CI) for comorbid respiratory and dermatologic conditions*. *Fully adjusted models with sociodemographic characteristics (age, gender, Hispanic origin, race, marital status, employment, household income), headache pain intensity and headache frequency. Reference groups were low headache pain intensity for headache pain intensity and low frequency headache (1–4 MHDs) for headache frequency

The respiratory conditions in this analysis were asthma and allergy/hay fever (Fig. 6). The risk of asthma was lower with increasing age, being non-Hispanic and being White, and the risk was higher with increasing MHD frequency category (Additional file 7O). The risk of allergy/hay fever was higher with increasing age, increasing MHD frequency category and both moderate and severe headache pain intensity, and lower for White and married respondents (Additional file 7P).

The dermatologic conditions in this analysis were psoriasis and rosacea (Fig. 6). The risk of psoriasis was not associated with age or other demographic characteristics except for being male (increased risk) and being employed (reduced risk). The only MHD frequency category associated with increased odds of psoriasis was the 10–14 MHD group and severe migraine headache pain intensity was also associated with increased odds (Additional file 7Q). Increasing age and being White were associated with increased risk of rosacea whereas being a man was associated with decreased risk. Only the highest MHD frequency category (≥21) and moderate headache pain intensity were associated with increased risk for rosacea (Additional file 7R).

#### Pain conditions

**Fig. 7:**
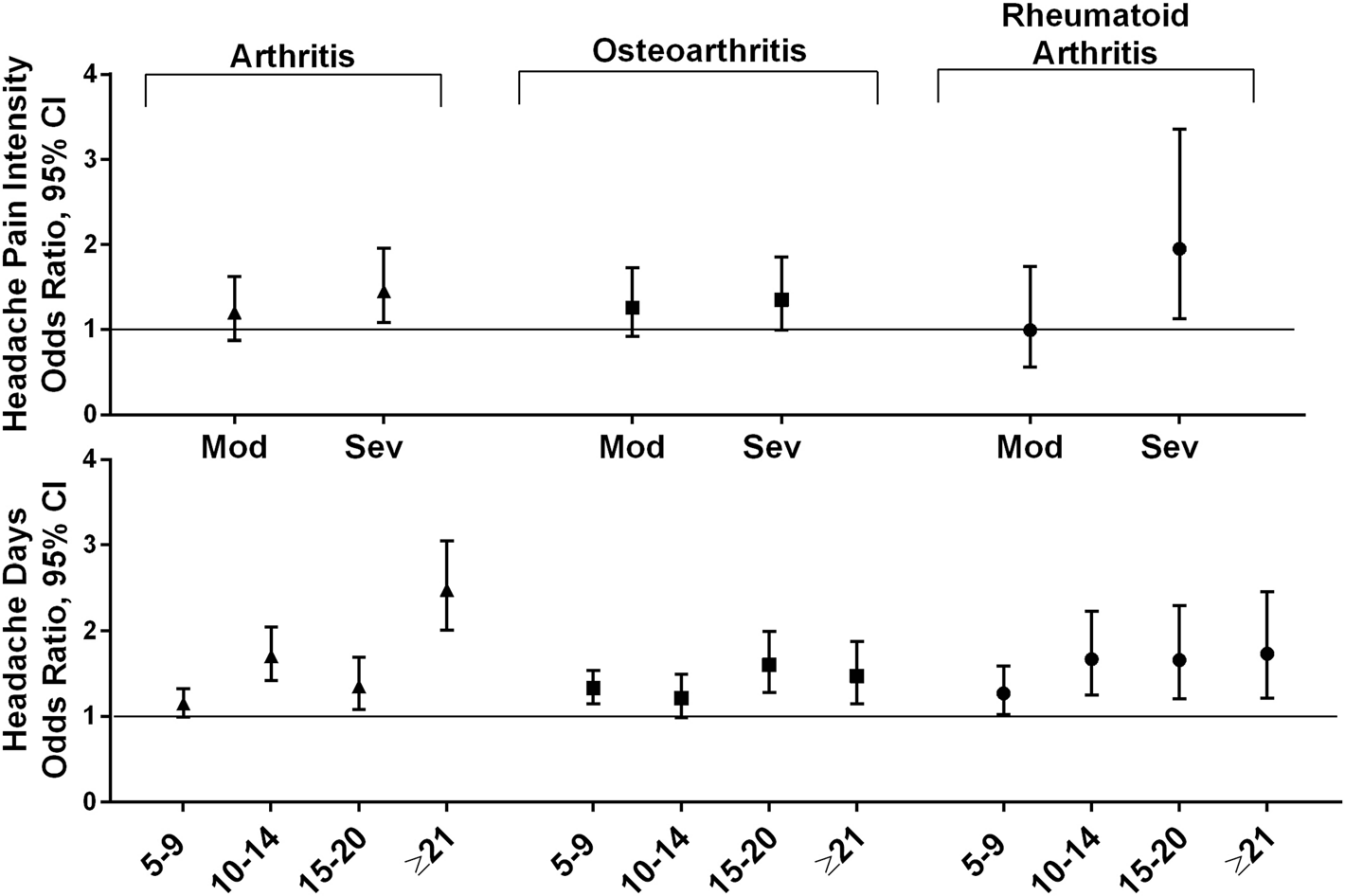
Among persons with migraine the odds ratios (and 95% CI) for each comorbid pain condition*. *Fully adjusted models with sociodemographic characteristics (age, gender, Hispanic origin, race, marital status, employment, household income), headache pain intensity and headache frequency. Reference groups are low headache pain intensity for headache pain intensity and low frequency headache (1–4 MHDs) for headache frequency

The pain conditions in this analysis were arthritis (type not known), osteoarthritis, and rheumatoid arthritis **(**Fig. 7**)**. A similar pattern of results was found for arthritis (type not known) and for rheumatoid arthritis. Age and being male were associated with increased risk, as was each MHD frequency category. Severe headache pain intensity for arthritis (type not known) and moderate headache pain intensity for rheumatoid arthritis were associated with increased risk, while being White, employed and increasing household income were associated with decreased risk (Additional files 7S and U). The pattern of results for osteoarthritis was the same for age and the other sociodemographic conditions, except that male gender was associated with lowered risk for this condition. As with arthritis (type not known), increasing MHD frequency category and severe headache pain intensity were associated with higher odds of osteoarthritis (Additional file 7T).

## Discussion

Many medical conditions are more common in people with migraine compared to the general population [33]. There is a small body of literature examining rates of single medical conditions among people with and without migraine, and over the past decade, a growing body of literature has emerged examining the effect of headache day frequency on rates of migraine comorbidities and the effects of selected comorbidities on headache progression [3–19, 22–28]. After adjusting for sociodemographic variables, the cohort with migraine was about three times more likely to report a medical diagnosis of insomnia, depression, anxiety, and gastric ulcer/GI bleeding. Those with migraine were at least twice as likely to report a medical diagnosis of peripheral artery disease, angina, allergies/hay fever, epilepsy, arthritis (type unknown), stroke or TIA, rheumatoid arthritis, asthma, and vitamin D deficiency. We also found that headache pain intensity was associated with higher risk for comorbidities including gastric ulcer disease, inflammatory disorders (psoriasis, allergy) and psychiatric (anxiety, depression) and sleep conditions (insomnia). Increasing MHD frequency was associated with increased risks for nearly all conditions. This effect was prominent among those with gastric ulcers/GI bleeding, diabetes, anxiety, depression, insomnia, asthma and allergies/hay fever.

Understanding comorbidity has implications for clinical care and for revealing disease pathophysiology and genetics. Although there are several potential mechanisms associated with the presence of comorbidities in migraine (i.e., artifact, uni- or bi-directional causality, shared environmental or genetic risk factors, among others), the identification of common comorbidities can enhance accurate diagnosis, effective treatment, adherence to treatment, prognosis, and minimization of disease burden [32]. However, it is possible that this population of people who self-reported medical diagnoses may be more likely to seek medical treatment. This Berkson’s bias may artificially inflate rates of comorbidity identification and diagnosis and can potentially influence the study findings [52, 53]. It should be noted that in a cross-sectional study such as this one, we cannot distinguish these influences and cannot determine if they are mutually exclusive. Nevertheless, healthcare professionals should be vigilant in assessing and treating comorbidities (or referring for treatment as appropriate). Since in many cases, they are known to complicate care and lead to poor outcomes, identifying and treating comorbidities may ease the burden of migraine and improve a variety of outcomes for patients, and is a key component of good clinical practice.

Several other studies have assessed individual co-occurring conditions and/or comorbidities with migraine that were also assessed in this MAST Study [3–19, 28]. In a large epidemiologic analysis conducted in Scotland, comorbidities observed in those with chronic migraine vs. non-migraine controls were evaluated. In that analysis, “chronic migraine” was defined differently from the *ICHD* definition as patients who had ≥4 prescriptions for migraine in the past 12 months, which is in contrast to the current MAST Study which used modified *ICHD-3* migraine criteria. Thirty-one comorbid health conditions and 7 mental health conditions were examined, of which 25 of the 31 health conditions and 3 of the 7 mental health conditions were found to occur with greater frequency in people with migraine [36]. The odds ratios observed in the MAST Study align with many of the comorbidities assessed in the Scottish study further substantiating these results.

Furthermore, several studies have reported that rates of comorbidities increase by headache day frequency among people with migraine comparing people with EM and CM and/or stratifying by low, moderate and high frequency EM [26, 30, 31, 37–41]. The existence of the comorbidities that we found with migraine are in general alignment with data reported in the scientific literature, although rates (whether odds ratios or percentages) vary by condition and by study, likely due in some part to methodological and measurement issues as well as differences in the subjects studied.

### Cardiovascular conditions

Severe headache pain intensity was associated with an increased risk of peripheral artery disease, hypertension, and high cholesterol but no association was found for angina and myocardial infarction. Increasing MHD frequency was associated with increasing risk for angina (5–9 MHDs, ≥21 MHDs) and hypertension (5–9 MHDs, ≥21 MHDs). No association with MHD frequency was found for peripheral artery disease, myocardial infarction or high cholesterol.

In a large Scottish database analysis, McLean et al. found the rates of peripheral artery disease were higher in migraine versus non-migraine controls (1.8% vs. 1.6%; OR 1.29 [1.10, 1.50]); a finding that was similar to the present report (1.8% vs. 1.2%; OR 2.69 [2.3, 3.1]) [36]. In a recent meta-analysis of 16 cohort studies, major adverse cardiovascular and cerebrovascular events were increased (adjusted hazard ratio 1.42 [1.26, 1.60], *P* < 0.001), an effect largely attributed to an increased risk of stroke and myocardial infarction among those with migraine. Interestingly, the longer the follow-up period, the greater the risk of developing the cardiovascular outcomes [3].

In a large Danish cohort study of 51,032 patients with migraine and 510,320 matched controls followed over 19 years, migraine was associated with increased risk of several cardiovascular events including myocardial infarction, stroke, venous thromboembolism, atrial fibrillation, and atrial flutter. The associations were stronger among women and in those with aura [4]. In another study that included patients with primary headache disorders, the OR of stroke was found to be 1.49 (1.15, 1.98) times higher than those without headache [54], a finding that was similar to the MAST Study (OR 2.18 [1.9, 2.5]).

Migraine and cardiovascular diseases have a few common etiologic links, including increased platelet aggregation, von Willebrand factor, hypercoagulable states, patent foramen ovale, shared genetic predisposition, higher frequency of cardiovascular risk factors, endothelial dysfunction, and increased sensitivity to ischemic injury [55]. In addition, cortical spreading depression may predispose to cerebral hypoperfusion; though this rarely falls below the ischemic threshold in true migrainous infarction, aura may directly contribute to the development of stroke [3, 56]. However, in the DUST (Dutch Acute Stroke Study) Study, patients with ischemic stroke who had a history of migraine did not have excess atherosclerosis in their large vessels suggesting that there may be mechanisms other than macrovascular cerebral atherosclerosis that may overlap in migraine and stroke [57]. However, in the present study, increasing MHD frequency was associated with increased risk for stroke/TIA (5–9 MHDs OR 1.54 [1.13, 2.1], ≥21 MHDs OR 1.94 [1.22, 3.07]).

### Neurologic conditions

Headache pain ratings were not associated with increased risk for the occurrence of epilepsy or for stroke/TIA. There was also no association between increasing MHD frequency and epilepsy risk. In the MAST Study, epilepsy was reported in 1.5% and 0.6% of the migraine and non-migraine sample, respectively (OR 2.33 [2.0, 2.8]). This finding was similar to findings from the Epilepsy Family Study of Columbia University were the relative risk of migraine and stroke was 2.4 [58]. A large Scottish analysis that found that epilepsy occurred in 2.0% of the migraine group and 0.9% in the non-migraine group (OR 2.19 [1.89, 2.54]) [36] However, in an analysis of patients with epilepsy who were asked if they have headache, the prevalence of migraine was 15% and was similar to prevalence figures in the general population, but far less than that observed in this analysis [59]. Overlapping mechanisms in epilepsy and migraine, such as mutations in genes for ion channels or neurotransmitter systems may contribute to comorbidities [60]. Some environmental risk factors, such as traumatic brain injury may also contribute.

### General medical conditions

Severe headache pain intensity was associated with an increased risk of gastric ulcer/GI bleeding (OR 2.31 [1.32, 4.05]), vitamin D deficiency (OR 1.58 [1.26, 1.99]), and diabetes (OR 1.45 [1.06, 1.98]), but no association with pain intensity was found for kidney disease. Increasing MHD frequency was associated with increasing risk for all general medical conditions studied: gastric ulcer/GI bleeding (5–9 MHDs OR 1.24 [1.01, 1.52], ≥21 MHDs OR 2.21 [1.63, 2.99]), kidney disease (5–9 MHDs OR 0.95 [0.68, 1.34], ≥21 MHDs OR 1.52 [0.93, 2.49]), vitamin D deficiency (5–9 MHDs OR 1.08 [0.97, 1.19], ≥21 MHDs OR 1.66 [1.39, 1.99]) and diabetes (5–9 MHDs OR 1.20 [1.04, 1.40], ≥21 MHDs OR 2.00 [1.59, 2.51]). It is possible that increasing rates of GI disease are associated with increasing MHDs due to an increase in acute medication use (such as nonsteroidal anti-inflammatory drugs [NSAIDs]) or perhaps there is a shared underlying pathophysiology.

In a systematic literature review of migraine and metabolic syndrome, 15 observational epidemiologic studies showed an increase in 11-year incidence in metabolic syndrome among those with migraine with aura (21.1%) or migraine without aura (16.8%) relative to those without headaches (14.5%). The majority of the subjects assessed were women, findings that are reflective of this MAST Study [61]. Similarly, the incidence of chronic kidney disease has been shown in this survey and others to be significantly increased in patients with migraine compared to those without migraine [62].

### Psychiatric and sleep conditions

Severe headache pain intensity was associated with an increased risk for all 3 of the conditions in this category: anxiety (OR 2.05 [1.67, 2.52]), depression (OR 2.13 [1.71, 2.66]), and insomnia (OR 2.20 [1.72, 2.81]). Increasing MHD frequency was associated with increasing risk for anxiety (5–9 MHDs OR 1.33 [1.22, 1.46], ≥21 MHDs OR 2.13 [1.8, 2.53]), depression (5–9 MHDs 1.31 [1.2, 1.44], ≥21 MHDs OR 2.26 [1.91, 2.69]) and insomnia (5–9 MHDs 1.47 [1.33, 1.63], ≥21 MHDs OR 2.46 [2.07, 2.94]).

In a large cross-sectional population study, people with probable migraine (based on *ICHD-2* criteria) were evaluated for sleep using the Pittsburgh Sleep Quality Index scale [63]. Results showed that the prevalence of poor sleep quality was significantly higher among those with migraine and probable migraine than among those without headache (47.6% [migraine] 35.4% [probable migraine vs. 21.0% [non-headache], *P* < 0.001) Interestingly, in this study, those with comorbid migraine and poor sleep quality also had a statistically significant higher prevalence of anxiety and depression and had migraine attacks that were more intense and of longer duration. As a result, a recent algorithm to manage sleep disorders and migraine has been developed which focuses on making sleep management complementary to headache management [64].

Many studies have observed increased depression and anxiety in patients who also report migraine [65–68], and there is evidence to support that anxiety and/or depression may be impacted by increasing headache frequency [66]. In an outpatient psychiatric clinic setting, patients with major depression were followed in a longitudinal manner for 10 years [69]. Headache was diagnosed based on *ICHD-2* (2-year assessment) or *ICHD-3b* (10-year assessments). Relative to those without migraine, those with migraine experienced reduced health-related quality of life (pain dimension), greater pain severity, and greater severity of anxiety and depression, each of which was independently associated with migraine. As migraine frequency increased, patients with depression experienced more pain-related symptoms. A recent review highlighted the bidirectional relationship between migraine and major depression as well as panic disorder, and underscored the importance of screening for psychiatric comorbidities and an integrated care approach to managing these conditions including the increased risk of suicide [20]. In the Scottish analysis by McLean and in this MAST Study, the OR for anxiety and depression all approximated 3, indicating a strong association with migraine.

### Respiratory and dermatologic conditions

Severe headache pain intensity was associated with an increased risk of allergy (OR 1.58 [1.32, 1.88]) and for psoriasis (OR 2.78 [1.51, 5.11]), but no association with pain was found for asthma or for rosacea. Increasing MHD frequency was associated with increasing risk for asthma (5–9 MHDs OR 1.10 [0.99, 1.23], ≥21 MHDs OR 1.84 [1.53, 2.22]), allergy/hay fever (5–9 MHDs 1.23 [1.13, 1.34], ≥21 MHDs OR 1.79 [1.51, 2.12]), and rosacea (5–9 MHDs 0.93 [0.76, 1.15], ≥21 MHDs OR 1.47[1.05, 2.05]) but not for psoriasis.

Rhinitis has been associated with migraine and may be aligned with allergies and hay fever. In the AMPP Study, frequency of migraine was increased in those who also reported rhinitis [41]. The risk of asthma in patients with migraine has been shown to be increased in a population-based cohort study of 6647 adult patients and comorbid asthma is associated with increased risk of new onset CM [25]. 

### Pain conditions

Severe headache pain intensity was associated with an increased risk of occurrence for all three pain conditions: arthritis (type not known) (OR 1.45 [1.08, 1.96]), osteoarthritis (OR 1.35 [0.99, 1.85]), and rheumatoid arthritis (OR 1.95 [1.13, 3.36]). Increasing MHD frequency was also associated with increasing risk of the pain conditions studied: arthritis (type not known) (5–9 MHDs OR 1.15 [0.99, 1.32], ≥21 MHDs OR 2.48 [2.01, 3.05]), osteoarthritis (5–9 MHDs 1.33 [1.15, 1.54], ≥21 MHDs OR 1.47 [1.15, 1.88]) and rheumatoid arthritis (5–9 MHDs 1.27 [1.02, 1.59], ≥21 MHDs OR 1.73 [1.21, 2.46]).

### Understanding migraine and comorbidities impacts clinical care

Several theories have been proposed to explain underlying mechanisms that could promote comorbid conditions, including the notion that one disease may cause the other, latent brain state models, shared environment, and shared genetic origins [32]. There are many reasons that understanding comorbidity is important, both for understanding a disease and also to facilitate disease management. In terms of clinical care, comorbidity may complicate diagnosis. Diagnostic parsimony may lead to under-diagnosis. Comorbidity may both inform and limit treatment. For example, cardiac comorbidity may limit the ability to use triptans, and GI comorbidity may limit the ability to use NSAIDs. However, knowing that a patient has both migraine and depression may lead a clinician to consider a preventive medication choice that is also an antidepressant. Comorbidity can also help predict prognosis. Several comorbidities have been identified as risk factors for the new onset of CM among people with EM. The identification of endophenotypes by comorbidity constellations may facilitate genetic research. Finally, comorbidity contributes to disease burden, both on an individual level where it is associated with increased disability and reduced health related quality of life [17] and on a societal level where it is associated with greater economic impact and burden [35]. While it has been established that treating migraine improves some of these comorbidities (e.g., depression and anxiety), it is less well known if treating the comorbidities improve or reduce migraine frequency, severity, or associated disability. Continued exploration of these questions would be valuable for improving patient care and outcomes.

### Limitations and strengths

Several limitations to this study should be considered when reviewing these data. Inclusion criteria for the population of migraine respondents was limited to those with active headache (≥1 MHD on average). Thus, these results cannot be applied to people with very infrequent headaches. In addition, since the MAST migraine sample may have more headache days, respondents may also be more likely to seek medical treatment, leading to higher rates of identification and diagnosis of comorbidities (Berksons’ bias). It should also be noted that some control sample respondents with adequate access to care and effective treatment may have had minimal or milder headache features and could be incorrectly classified as non-migraine. In this survey, not all possible comorbidities were collected. A large and ever expanding range of comorbidities have been associated with migraine, including irritable bowel disease, atrial fibrillation, Crohn’s disease, personality disorders, sleep apnea, fibromyalgia, Raynaud’s disease, and many more conditions that were not included in the MAST survey due to limitations of space and an attempt to minimize respondent fatigue [36]. In a previous study using a similar methodology (the CaMEO Study), noncephalic pain was a marker for the new onset of CM among a sample of people with EM [71]. Pain was not fully addressed in this analysis and other pain conditions may be part of the multi-comorbidity profile for some MAST Study respondents. As is common in observational research, a healthcare professional diagnosis of comorbid conditions was obtained via respondent self-report only. Healthcare professional report, electronic health records, and claims data were not available to confirm a self-reported diagnosis, and we do not know if the self-reported condition is currently active which could have led to overestimates in reporting. On the other hand, there are several conditions that we assessed which are commonly underdiagnosed and often not even discussed with healthcare professionals, such as insomnia, depression and anxiety, leading to a possible underestimate of the rates for these comorbidities. Access to care could also impact the rate and accuracy of self-reported provider diagnosis and we are also lacking data on this variable. The migraine and non-migraine control sample populations had distinct demographic differences, especially with respect to age and gender. However, the regression modeling took into account these differences when calculating the adjusted odds of reporting a given condition.

There are several strengths to this analysis. The web-based survey was drawn from a demographically diverse US population balanced to the US Census. The resulting migraine cohort was comparable to other migraine population studies [51], and the non-migraine cohort was demographically similar to the US population. Although respondents self-reported experiencing migraine or headache in the past year, modified *ICHD-3* criteria also had to be met using a validated screener in order to be included in the migraine cohort. Respondents were also asked to confirm that a healthcare professional gave them a diagnosis of the conditions that we assessed, which increased the reliability of reporting. And finally, in assessing comorbid health problem risk in the population, comparisons between the migraine and non-migraine cohorts were adjusted for sociodemographic covariates; and this was also true in assessing the potential incremental comorbid health problem risk associated with headache pain intensity and increasing MHD frequency within the migraine cohort. To our knowledge, this is the first analysis examining the effect of headache pain intensity on rates of migraine comorbidities.

## Conclusions

These results are a reminder that people with migraine may experience a wide range of additional diseases and health problems that also must be effectively managed, and that the risk of these problems increases with increases in headache day frequency and headache pain intensity. Future work is required to differentiate the causal sequence (direct causality, reverse causality, shared underlying predisposition), the potential confounding role of migraine treatment (i.e., NSAIDs may lead to gastric ulcers), as well as shared risk factors or potential detection bias. Exploration of the pathways that drive these comorbidities in migraine patients may lead to insights that clarify pathophysiology and improve treatment.

## Supplementary information

**Additional file 1.** Participant Flow Chart. *After consenting and removing incomplete surveys and respondents who did not meet inclusion criteria or failed quality control checks.

**Additional file 2.** Baseline Demographic Characteristics for the Migraine Cohort by Monthly Headache Day Frequency.

**Additional file 3.** Baseline Demographic Characteristics for the Migraine Cohort by Headache Pain Intensity Rating.

**Additional file 4.** Percent of Respondents with a Self-reported Healthcare Professional Diagnosis of a Comorbid Condition. Odds ratio (adjusted for sociodemographics) and 95% Confidence Interval (reference = non-migraine cohort).

**Additional file 5.** Percent of Respondents with a Diagnosis of a Comorbid Condition Based on Headache Pain Intensity Rating (Low = 1–3; Moderate = 4–6; Severe- ≥ 7).

**Additional file 6.** Percent of Respondents with a Diagnosis of a Comorbid Condition Based on the Number of Monthly Headache Days.

**Additional file 7.** 7A-7 U. Logistic Regression Modeling for each Co-morbid Condition. Model 1 for the Overall Population (*N* = 92,586), Contrasts Migraine and Non-migraine groups Adjusting for Sociodemographics (Non-migraine is the Reference Group). Model 2 in the Migraine Sample (*N* = 15,133) Looks at the Impact of Increasing MHD frequency (1–4, 5–9, 10–14, 15–20, ≥21 MHDs) Adjusting for Sociodemographics (1–4 MHD is the Reference Group). Model 3 in the Migraine Sample (N = 15,133) looks at the impact of Headache Pain Intensity Ratings (Low, Moderate, Severe) Adjusting for Sociodemographics (Low Headache Pain Intensity is the Reference Group). Model 4 in the Migraine sample (N = 15,133) Looks at the Combined Effect of MHD Frequency and Headache Pain Intensity Adjusting for Sociodemographics. Odds ratios and 95% Confidence Intervals are provided.

## Data Availability

All data generated or analyzed during this study are included in this published article [and its supplementary information files].

## Abbreviations

AMPP: American Migraine Prevalence and Prevention
AMS: American Migraine Study
CaMEO: Chronic Migraine Epidemiology and Outcomes
CI: Confidence interval
CM: Chronic migraine
EM: Episodic migraine
GI: Gastrointestinal
ICHD: International Committee of Headache Disorders
MAST: Migraine in America Symptoms and Treatment
MHD: Monthly headache days
MIDAS: Migraine Disability Assessment
NSAIDS: Nonsteroidal anti-inflammatory drugs
OR: Odds ratio
TIA: Transient ischemic attack
US: United States
