# Supplementary figures and images for: Comorbid and co-occurring conditions in migraine and associated risk of increasing headache pain intensity and headache frequency: results of the migraine in America symptoms and treatment (MAST) study
Source: J Headache Pain. 2020 Mar 2;21(1):23. doi: 10.1186/s10194-020-1084-y (PMC7053108; doi:10.1186/s10194-020-1084-y)

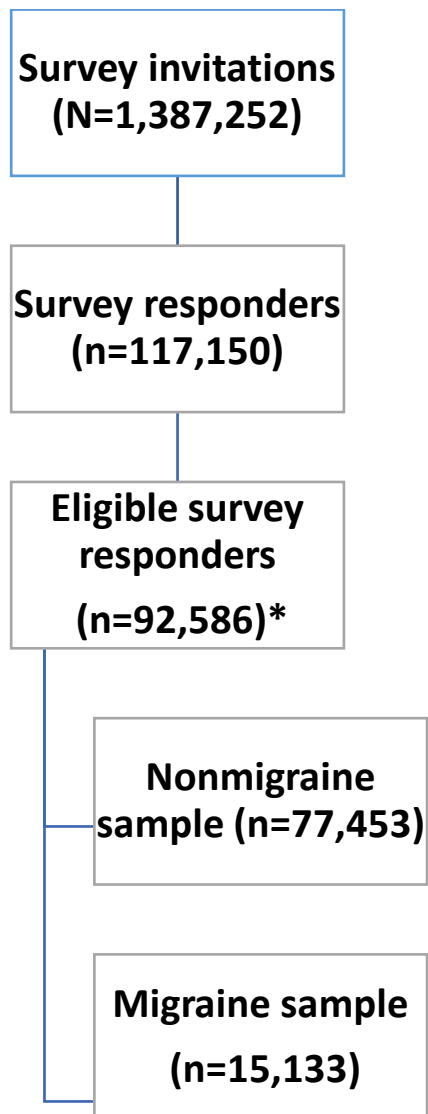

Supplement: Supplementary file 1 — Additional file 1. Participant Flow Chart. *After consenting and removing incomplete surveys and respondents who did not meet inclusion criteria or failed quality control checks. [file 10194_2020_1084_MOESM1_ESM.pdf]
