# Supplementary material for: Comorbid and co-occurring conditions in migraine and associated risk of increasing headache pain intensity and headache frequency: results of the migraine in America symptoms and treatment (MAST) study
Source: J Headache Pain. 2020 Mar 2;21(1):23. doi: 10.1186/s10194-020-1084-y (PMC7053108; doi:10.1186/s10194-020-1084-y)
Supplement: Supplementary file 2 — Additional file 2. Baseline Demographic Characteristics for the Migraine Cohort by Monthly Headache Day Frequency. [file 10194_2020_1084_MOESM2_ESM.docx]

**Additional File 2:** Baseline Demographic Characteristics for the Migraine Cohort by Monthly Headache Day Frequency.

|  | **1 -4 MHDs**  **(n=9,471)** | **5 -9 MHDs**  **(n=3,011)** | **10-14 MHDs**  **(n=1,193)** | **15-20 MHDs**  **(n=828)** | **≥21 MHDs**  **(n=630)** | **Chi/ F Statistic** | ***P*-value** |
| --- | --- | --- | --- | --- | --- | --- | --- |
| **Mean age, years (SD)** | 43.2 (13.6) | 42.1 (13.5) | 42.5 (13.7) | 44.4 (13.9) | 46.0 (13.8) | F=931.060 | <0.001 |
| **Age, n (%)** |  |  |  |  |  | 4,538.956 | <0.001 |
| 18-24 years | 669 (7.1) | 275 (9.1) | 102 (8.5) | 63 (7.6) | 38 (6.0) |  |  |
| 25-34 years | 2,338 (24.7) | 755 (25.1) | 313 (26.2) | 170 (20.5) | 98 (15.6) |  |  |
| 35-44 years | 2,294 (24.2) | 743 (24.7) | 259 (21.7) | 194 (23.4) | 158 (25.1) |  |  |
| 45-54 years | 2,067 (21.8) | 679 (22.6) | 273 (22.9) | 203 (24.5) | 164 (26) |  |  |
| 55-64 years | 1,356 (14.3) | 350 (11.6) | 162 (13.6) | 119 (14.4) | 104 (16.5) |  |  |
| ≥65 years | 747 (7.9) | 209 (6.9) | 84 (7.0) | 79 (9.5) | 68 (10.8) |  |  |
| **Women, n (%)** | 6,771 (71.5) | 2,290 (76.1) | 892 (74.8) | 651 (78.6) | 445 (70.6) | 3,630.151 | <0.001 |
| **White, n (%)** | 7247 (76.5) | 2,397 (79.6) | 946 (79.3) | 660 (79.7) | 505 (80.2) | 24.958 | <0.001 |
| **Hispanic Origin, yes, n (%)** | 981 (10.4) | 300 (10.0) | 119 (10.1) | 81 (9.9) | 65 (10.4) | 149.364 | <0.001 |
| **Married, n (%)** | 5,137 (54.2) | 1,588 (52.7) | 654 (54.8) | 430 (51.9) | 346 (54.9) | 228.607 | <0.001 |
| **Employed, n (%)** | 7,023 (74.2) | 2,183 (72.5) | 827 (69.3) | 510 (61.6) | 360 (57.1) | 688.254 | <0.001 |
| **Annual Household Income, n (%)** |  |  |  |  |  | 658.809 | <0.001 |
| <$25,000 | 1,010 (11.0) | 372 (12.7) | 164 (14.2) | 144 (17.8) | 115 (18.9) |  |  |
| $25,000−$49,000 | 1,925 (20.9) | 660 (22.5) | 274 (23.7) | 198 (24.4) | 145 (23.9) |  |  |
| $50,000−$74,999 | 2,033 (22.1) | 616 (21.0) | 267 (23.1) | 167 (20.6) | 134 (22.1) |  |  |
| $75,000−$99,999 | 1680 (18.3) | 501 (17.1) | 179 (15.5) | 131 (16.2) | 80 (13.2) |  |  |
| ≥$100,000 | 2,547 (27.7) | 779 (26.6) | 271 (23.5) | 171 (21.1) | 133 (21.9) |  |  |
