# Supplementary material for: Comorbid and co-occurring conditions in migraine and associated risk of increasing headache pain intensity and headache frequency: results of the migraine in America symptoms and treatment (MAST) study
Source: J Headache Pain. 2020 Mar 2;21(1):23. doi: 10.1186/s10194-020-1084-y (PMC7053108; doi:10.1186/s10194-020-1084-y)
Supplement: Supplementary file 3 — Additional file 3. Baseline Demographic Characteristics for the Migraine Cohort by Headache Pain Intensity Rating. [file 10194_2020_1084_MOESM3_ESM.docx]

**Additional File 3.** Baseline Demographic Characteristics for the Migraine Cohort by Headache Pain Intensity Rating.

|  | **Mild Pain**  **(n=610)** | **Moderate Pain**  **(n=5762)** | **Severe Pain**  **(n=8759)** | **Chi/ F Statistic** | ***P*-value** |
| --- | --- | --- | --- | --- | --- |
| **Mean age, years (SD)** | 45.5 (15.0) | 42.3 (14.0) | 43.5 (13.6) | F=23.762 | <0.001 |
| **Age, n (%)** |  |  |  | 127.973 | <0.001 |
| 18-24 years | 45 (7.4) | 557 (9.7) | 545 (6.2) |  |  |
| 25-34 years | 138 (22.6) | 1478 (25.7) | 2057 (23.5) |  |  |
| 35-44 years | 116 (19) | 1341 (23.3) | 2191 (25) |  |  |
| 45-54 years | 120 (19.7) | 1190 (20.7) | 2076 (23.7) |  |  |
| 55-64 years | 116 (19) | 726 (12.6) | 1248 (14.2) |  |  |
| ≥65 years | 75 (12.3) | 470 (8.2) | 642 (7.3) |  |  |
| **Women, n (%)** | 359 (58.9) | 4140 (71.9) | 6548 (74.8) | 79.552 | <0.001 |
| **White, n (%)** | 516 (84.6) | 4643 (80.6) | 6594 (75.3) | 73.767 | <0.001 |
| **Hispanic Origin, yes, n (%)** | 36 (5.9) | 495 (8.7) | 1015 (11.7) | 46.682 | <0.001 |
| **Married, n (%)** | 339 (55.6) | 3038 (52.7) | 4776 (54.5) | 5.273 | NS |
| **Employed, n (%)** | 422 (69.2) | 4334 (75.2) | 6147 (70.2) | 46.421 | <0.001 |
| **Annual Household Income, n (%)** |  |  |  | 42.646 | <0.001 |
| <$25,000 | 52 (8.9) | 594 (10.7) | 1159 (13.6) |  |  |
| $25,000−$49,000 | 114 (19.4) | 1214 (21.8) | 1873 (22) |  |  |
| $50,000−$74,999 | 123 (21) | 1246 (22.4) | 1847 (21.6) |  |  |
| $75,000−$99,999 | 118 (20.1) | 1005 (18) | 1448 (17) |  |  |
| ≥$100,000 | 180 (30.7) | 1515 (27.2) | 2206 (25.9) |  |  |
