# Supplementary material for: Comorbid and co-occurring conditions in migraine and associated risk of increasing headache pain intensity and headache frequency: results of the migraine in America symptoms and treatment (MAST) study
Source: J Headache Pain. 2020 Mar 2;21(1):23. doi: 10.1186/s10194-020-1084-y (PMC7053108; doi:10.1186/s10194-020-1084-y)
Supplement: Supplementary file 4 — Additional file 4. Percent of Respondents with a Self-reported Healthcare Professional Diagnosis of a Comorbid Condition. Odds ratio (adjusted for sociodemographics) and 95% Confidence Interval (reference = non-migraine cohort). [file 10194_2020_1084_MOESM4_ESM.docx]

**Additional File 4.** Percent of Respondents with a Self-reported Healthcare professional Diagnosis of a Comorbid Condition. Odds ratio (adjusted for sociodemographics) and 95% Confidence Interval (reference=non-migraine cohort)

|  | **Migraine Sample, % (n=15,133)** | **Non-migraine Sample, % (n=77,453)** | **Total Sample, % (N=92,586)** | **OR (95% CI)**** |
| --- | --- | --- | --- | --- |
| **Cardiovascular** |  |  |  |  |
| Angina | 3.2 | 2.5 | 2.6 | 2.64 (2.36, 2.95) |
| Peripheral Artery Disease | 1.8 | 1.2 | 1.3 | 2.69 (2.32, 3.13) |
| *Myocardial infarction | 1.4 | 2.2 | 2.1 | 1.66 (1.43, 1.93) |
| *Hypertension | 23.8 | 28.1 | 27.4 | 1.51 (1.44, 1.58) |
| *High Cholesterol | 25.4 | 29.6 | 28.9 | 1.61 (1.53, 1.68) |
| **Neurologic** |  |  |  |  |
| Epilepsy | 1.5 | 0.6 | 0.7 | 2.33 (1.96, 2.76) |
| Stroke or TIA | 1.8 | 1.6 | 1.6 | 2.18 (1.89, 2.53) |
| **General Medical** |  |  |  |  |
| Gastric Ulcer/ GI Bleeding | 4.5 | 1.9 | 2.3 | 3.11 (2.81, 3.45) |
| *Kidney Disease | 1.7 | 1.8 | 1.8 | 1.48 (1.28, 1.71) |
| Vitamin D Deficiency | 23.3 | 12.1 | 14.0 | 2.00(1.91, 2.10) |
| *Diabetes | 9.3 | 10.4 | 10.2 | 1.37 (1.28, 1.46) |
| **Psychiatric** |  |  |  |  |
| Anxiety | 34.8 | 11.4 | 15.2 | 3.18 (3.04, 3.32) |
| Depression | 30.5 | 10.1 | 13.4 | 3.18 (3.03, 3.32) |
| Insomnia | 23.1 | 7.5 | 10.0 | 3.79 (3.6, 3.98) |
| **Respiratory** |  |  |  |  |
| Asthma | 18.8 | 9.0 | 10.6 | 2.03 (1.93, 2.14) |
| Allergies/Hay fever | 48.2 | 26.2 | 29.8 | 2.49 (2.39, 2.59) |
| **Dermatologic** |  |  |  |  |
| Psoriasis | 4.2 | 2.5 | 2.8 | 1.98 (1.79, 2.18) |
| Rosacea | 4.8 | 3.2 | 3.4 | 1.68 (1.53, 1.84) |
| **Pain** |  |  |  |  |
| Arthritis-Type Unknown | 10.8 | 8.3 | 8.7 | 2.20 (2.07, 2.35) |
| Osteoarthritis | 10.3 | 9.0 | 9.2 | 1.88 (1.76, 2.01) |
| Rheumatoid Arthritis | 3.6 | 2.0 | 2.3 | 2.11 (1.89, 2.35) |

CI=confidence interval; GI=gastrointestinal; OR=odds ratio; TIA=transient ischemic attack.

*Raw percentages are in the opposite direction of the OR in Figure 1 due to demographic differences, primarily age.

**Because of the large number of covariates only adjusted odds ratios are presented.
