# Supplementary material for: Comorbid and co-occurring conditions in migraine and associated risk of increasing headache pain intensity and headache frequency: results of the migraine in America symptoms and treatment (MAST) study
Source: J Headache Pain. 2020 Mar 2;21(1):23. doi: 10.1186/s10194-020-1084-y (PMC7053108; doi:10.1186/s10194-020-1084-y)
Supplement: Supplementary file 5 — Additional file 5. Percent of Respondents with a Diagnosis of a Comorbid Condition Based on Headache Pain Intensity Rating (Low = 1–3; Moderate = 4–6; Severe- ≥ 7). [file 10194_2020_1084_MOESM5_ESM.docx]

**Additional File 5.** Percent of Respondents with a Diagnosis of a Comorbid Condition Based on Headache Pain Intensity Rating (Low=1−3; Moderate=4-6; Severe-≥7).

|  | **Total Migraine  (n=15,131)** | **Low Pain**  **(n=610)** | **Moderate Pain**  **(n=5,762)** | **Severe Pain**  **(n=8,759)** | |
| --- | --- | --- | --- | --- | --- |
|  | **% of Sample** | **% of Sample** | **% of Sample** | **% of Sample** |  |
| **Cardiovascular** | | | | |  |
| Angina | 3.2 | 3.8 | 2.4 | 3.7 |  |
| Peripheral Artery Disease | 1.8 | 0.8 | 1.5 | 2.1 |  |
| Myocardial infarction | 1.4 | 2.1 | 1.2 | 1.6 |  |
| Hypertension | 23.8 | 23.6 | 21.2 | 25.6 |  |
| High Cholesterol | 25.4 | 27.0 | 23.4 | 26.6 |  |
| **Neurologic** | | | | |  |
| Epilepsy | 1.5 | 1.3 | 1.1 | 1.8 |  |
| Stroke or TIA | 1.8 | 2.8 | 1.3 | 2.0 |  |
| **General Medical** | | | | |  |
| Gastric Ulcer/ GI Bleeding | 4.5 | 2.1 | 4.2 | 4.9 |  |
| Kidney Disease | 1.7 | 2.1 | 1.2 | 2.0 |  |
| Vitamin D Deficiency | 23.3 | 15.7 | 19.8 | 26.1 |  |
| Diabetes | 9.3 | 8.2 | 7.3 | 10.7 |  |
| **Psychiatric** | | | | |  |
| Anxiety | 34.8 | 20.8 | 30.2 | 38.8 |  |
| Depression | 30.5 | 17.7 | 26.1 | 34.3 |  |
| Insomnia | 23.1 | 13.3 | 17.7 | 27.3 |  |
| **Respiratory** | | | | |  |
| Asthma | 18.8 | 16.1 | 17.1 | 20.1 |  |
| Allergies/Hay fever | 48.2 | 36.6 | 46.0 | 50.5 |  |
| **Dermatologic** | | | | |  |
| Psoriasis | 4.2 | 1.8 | 3.7 | 4.8 |  |
| Rosacea | 4.8 | 3.4 | 5.2 | 4.6 |  |
| **Pain** | | | | |  |
| Arthritis-Unknown Type | 10.8 | 9.0 | 9.2 | 12.0 |  |
| Osteoarthritis | 10.3 | 8.9 | 9.1 | 11.2 |  |
| Rheumatoid Arthritis | 3.6 | 2.3 | 2.2 | 4.7 |  |

CI=confidence interval; GI=gastrointestinal; OR=odds ratio; TIA=transient ischemic attack
