# Supplementary material for: Comorbid and co-occurring conditions in migraine and associated risk of increasing headache pain intensity and headache frequency: results of the migraine in America symptoms and treatment (MAST) study
Source: J Headache Pain. 2020 Mar 2;21(1):23. doi: 10.1186/s10194-020-1084-y (PMC7053108; doi:10.1186/s10194-020-1084-y)
Supplement: Supplementary file 6 — Additional file 6. Percent of Respondents with a Diagnosis of a Comorbid Condition Based on the Number of Monthly Headache Days. [file 10194_2020_1084_MOESM6_ESM.docx]

**Additional File 6.** Percent of Respondents with a Diagnosis of a Comorbid Condition Based on the Number of Monthly Headache Days.

|  | **Total Migraine  (n=15,131)** | **1-4 MHDs**  **(n=9,471)** | **5 to 9 MHDs**  **(n=3,011)** | **10 to 14 MHDs (n=1,193)** | **15 to 20 MHDs (n=828)** | **≥21 MHDs**  **(n=630)** |  |  |
| --- | --- | --- | --- | --- | --- | --- | --- | --- |
|  | **% of Sample** | **% of Sample** | **% of Sample** | **% of Sample** | **% of Sample** | **% of Sample** |  |  |
| **Cardiovascular** | | | | | | | | |
| Angina | 3.2 | 2.5 | 3.7 | 4.1 | 5.7 | 5.7 | |  |
| Peripheral Artery Disease | 1.8 | 1.6 | 2.2 | 1.8 | 1.8 | 2.5 | |  |
| Myocardial infarction | 1.4 | 1.3 | 1.9 | 1.4 | 1.9 | 1.7 | |  |
| Hypertension | 23.8 | 22.6 | 23.1 | 26.4 | 31.2 | 32.1 | |  |
| High Cholesterol | 25.4 | 24.4 | 25.9 | 25.9 | 30.1 | 31.1 | |  |
| **Neurologic** | | | | | | | | |
| Epilepsy | 1.5 | 1.3 | 1.4 | 2.2 | 2.9 | 2.2 | |  |
| Stroke or TIA | 1.8 | 1.4 | 2.1 | 2.3 | 2.5 | 3.7 | |  |
| **General Medical** | | | | | | | | |
| Gastric Ulcer/ GI Bleeding | 4.5 | 3.8 | 4.7 | 6.1 | 6.8 | 8.6 | |  |
| Kidney Disease | 1.7 | 1.7 | 1.5 | 1.8 | 1.9 | 3.2 | |  |
| Vitamin D Deficiency | 23.3 | 21.7 | 23.4 | 27.4 | 27.9 | 32.9 | |  |
| Diabetes | 9.3 | 8.2 | 9.4 | 11.7 | 12.0 | 17.9 | |  |
| **Psychiatric** | | | | | | | | |
| Anxiety | 34.8 | 30.4 | 38.6 | 41.6 | 50.1 | 50.3 | |  |
| Depression | 30.5 | 26.2 | 33.0 | 39.1 | 45.3 | 47.6 | |  |
| Insomnia | 23.1 | 18.8 | 25.9 | 32.4 | 35.6 | 40.2 | |  |
| **Respiratory** | | | | | | | | |
| Asthma | 18.8 | 17.2 | 19.4 | 23.0 | 21.4 | 28.1 | |  |
| Allergies/Hay fever | 48.2 | 45.1 | 50.6 | 54.7 | 56.5 | 60.2 | |  |
| **Dermatologic** | | | | | | | | |
| Psoriasis | 4.2 | 3.9 | 4.5 | 5.3 | 5.4 | 4.9 | |  |
| Rosacea | 4.8 | 4.6 | 4.4 | 5.8 | 5.0 | 7.0 | |  |
| **Pain** | | | | | | | | |
| Arthritis-Unknown Type | 10.8 | 9.5 | 10.3 | 14.7 | 13.2 | 23.3 | |  |
| Osteoarthritis | 10.3 | 9.1 | 11.3 | 11.1 | 15.9 | 15.9 | |  |
| Rheumatoid Arthritis | 3.6 | 3.0 | 3.9 | 5.1 | 5.8 | 6.0 | |  |

CI=confidence interval; GI=gastrointestinal; OR=odds ratio; TIA=transient ischemic attack
