# Supplementary material for: Comorbid and co-occurring conditions in migraine and associated risk of increasing headache pain intensity and headache frequency: results of the migraine in America symptoms and treatment (MAST) study
Source: J Headache Pain. 2020 Mar 2;21(1):23. doi: 10.1186/s10194-020-1084-y (PMC7053108; doi:10.1186/s10194-020-1084-y)
Supplement: Supplementary file 7 — Additional file 7. 7A-7 U. Logistic Regression Modeling for each Co-morbid Condition. Model 1 for the Overall Population (N = 92,586), Contrasts Migraine and Non-migraine groups Adjusting for Sociodemographics (Non-migraine is the Reference Group). Model 2 in the Migraine Sample (N = 15,133) Looks at the Impact of Increasing MHD frequency (1–4, 5–9, 10–14, 15–20, ≥21 MHDs) Adjusting for Sociodemographics (1–4 MHD is the Reference Group). Model 3 in the Migraine Sample (N = 15,133) looks at the impact of Headache Pain Intensity Ratings (Low, Moderate, Severe) Adjusting for Sociodemographics (Low Headache Pain Intensity is the Reference Group). Model 4 in the Migraine sample (N = 15,133) Looks at the Combined Effect of MHD Frequency and Headache Pain Intensity Adjusting for Sociodemographics. Odds ratios and 95% Confidence Intervals are provided. [file 10194_2020_1084_MOESM7_ESM.docx]

**Additional File 7A-7U.** Logistic Regression Modeling for each Co-morbid Condition. Model 1 for the Overall Population (N=92,586), Contrasts Migraine and Non-migraine groups Adjusting for Sociodemographics (Non-migraine is the Reference Group). Model 2 in the Migraine Sample (N=15,133) Looks at the Impact of Increasing MHD frequency (1-4, 5-9, 10-14, 15-20, ≥21 MHDs) Adjusting for Sociodemographics (1-4 MHD is the Reference Group). Model 3 in the Migraine Sample (N=15,133) looks at the impact of Headache Pain Intensity Ratings (Low, Moderate, Severe) Adjusting for Sociodemographics (Low Headache Pain Intensity is the Reference Group). Model 4 in the Migraine sample (N=15,133) Looks at the Combined Effect of MHD Frequency and Headache Pain Intensity Adjusting for Sociodemographics. Odds ratios and 95% Confidence Intervals are provided.

| **Additional File 7A**  **Cardiovascular** | **MAST Overall Population  Migraine vs. Non-migraine Model (N=92,586)** | | **MAST Respondents with Migraine MHD Model**  **(N=15,133)** | | **MAST Respondents with Migraine Headache Pain Intensity Model  (N=15,133)** | | **MAST Respondents with Migraine**  **MHD + Headache Pain Intensity Model**  **(N=15,133)** | |
| --- | --- | --- | --- | --- | --- | --- | --- | --- |
| **Angina** | **OR (95%CI)** | **P-Value** | **OR (95%CI)** | **P-Value** | **OR (95%CI)** | **P-Value** | **OR (95%CI)** | **P-Value** |
| **Age (ref=18 -24)** |  |  |  |  |  |  |  |  |
| **25-34** | 1.15 (0.77, 1.73) | 0.500 | 1.38 (0.73, 2.61) | 0.317 | 1.31 (0.7, 2.48) | 0.401 | 1.34 (0.71, 2.52) | 0.371 |
| **35-44** | 1.31 (0.88, 1.95) | 0.191 | 2.02 (1.09, 3.74) | 0.026 | 1.9 (1.02, 3.53) | 0.042 | 1.93 (1.04, 3.58) | 0.038 |
| **45-54** | 2.17 (1.47, 3.19) | <0.001 | 3.36 (1.84, 6.15) | <0.001 | 3.17 (1.73, 5.81) | <0.001 | 3.21 (1.75, 5.88) | <0.001 |
| **55-64** | 3.26 (2.22, 4.78) | <0.001 | 3.7 (1.99, 6.87) | <0.001 | 3.4 (1.83, 6.33) | <0.001 | 3.54 (1.9, 6.59) | <0.001 |
| **≥65** | 5.6 (3.81, 8.23) | <0.001 | 5.81 (3.08, 10.96) | <0.001 | 5.45 (2.89, 10.28) | <0.001 | 5.67 (3, 10.7) | <0.001 |
| **Male** | 2.76 (2.5, 3.04) | <0.001 | 2.33 (1.92, 2.82) | <0.001 | 2.3 (1.9, 2.79) | <0.001 | 2.35 (1.94, 2.85) | <0.001 |
| **Non-Hispanic** | 1.03 (0.85, 1.25) | 0.762 | 1.07 (0.75, 1.51) | 0.724 | 1.1 (0.78, 1.56) | 0.595 | 1.08 (0.76, 1.53) | 0.667 |
| **Caucasian** | 1.08 (0.96, 1.23) | 0.199 | 0.86 (0.68, 1.1) | 0.234 | 0.89 (0.7, 1.14) | 0.360 | 0.88 (0.69, 1.12) | 0.307 |
| **Married** | 1.04 (0.94, 1.14) | 0.483 | 0.95 (0.77, 1.18) | 0.651 | 0.96 (0.78, 1.19) | 0.732 | 0.95 (0.77, 1.18) | 0.644 |
| **Employed** | 0.68 (0.61, 0.75) | <0.001 | 0.59 (0.48, 0.74) | <0.001 | 0.58 (0.47, 0.73) | <0.001 | 0.6 (0.48, 0.75) | <0.001 |
| **Household Income (ref=<$25,000)** |  |  |  |  |  |  |  |  |
| **$25,000 to $49,999** | 0.79 (0.67, 0.93) | 0.005 | 0.84 (0.61, 1.16) | 0.287 | 0.84 (0.61, 1.15) | 0.269 | 0.86 (0.62, 1.18) | 0.340 |
| **$50,000 to $74,999** | 0.7 (0.59, 0.82) | <0.001 | 0.68 (0.48, 0.95) | 0.026 | 0.66 (0.47, 0.93) | 0.017 | 0.69 (0.49, 0.97) | 0.032 |
| **$75,000 to $99,999** | 0.64 (0.54, 0.76) | <0.001 | 0.65 (0.45, 0.94) | 0.023 | 0.63 (0.44, 0.91) | 0.014 | 0.66 (0.46, 0.96) | 0.030 |
| **≥$100,000** | 0.63 (0.53, 0.74) | <0.001 | 0.85 (0.61, 1.2) | 0.353 | 0.81 (0.58, 1.14) | 0.223 | 0.86 (0.61, 1.21) | 0.382 |
| **Migraine Screen Positive** | 2.64 (2.36, 2.95) | <0.001 |  |  |  |  |  |  |
| **Monthly Headache Days (ref=1 to 4)** |  |  |  |  |  |  |  |  |
| **5 to 9** |  |  | 1.63 (1.29, 2.06) | <0.001 |  |  | 1.59 (1.26, 2.01) | <0.001 |
| **10 to 14** |  |  | 1.71 (1.24, 2.36) | 0.001 |  |  | 1.65 (1.19, 2.28) | 0.003 |
| **15 to 20** |  |  | 2.26 (1.63, 3.15) | <0.001 |  |  | 2.16 (1.55, 3.01) | <0.001 |
| **≥21** |  |  | 1.78 (1.22, 2.61) | 0.003 |  |  | 1.7 (1.16, 2.5) | 0.006 |
| **Headache Pain Intensity (ref=low pain, 1 to 3)** |  |  |  |  |  |  |  |  |
| **Moderate Pain, 4 to 6** |  |  |  |  | 0.8 (0.5, 1.26) | 0.332 | 0.76 (0.48, 1.2) | 0.242 |
| **Severe Pain, 7 to 10** |  |  |  |  | 1.21 (0.78, 1.89) | 0.392 | 1.08 (0.69, 1.69) | 0.727 |

| **Additional File 7B**  **Cardiovascular** | **MAST Overall Population  (N=92,586)** | | **MAST Respondents with Migraine MHD Model**  **(N=15,133)** | | **MAST Respondents with Migraine Headache Pain Intensity Model  (N=15,133)** | | **MAST Respondents with Migraine**  **MHD + Headache Pain Intensity Model**  **(N=15,133)** | |
| --- | --- | --- | --- | --- | --- | --- | --- | --- |
| **Peripheral Artery Disease** | **OR (95%CI)** | **P-Value** | **OR (95%CI)** | **P-Value** | **OR (95%CI)** | **P-Value** | **OR (95%CI)** | **P-Value** |
| **Age (ref=18 -24)** |  |  |  |  |  |  |  |  |
| **25-34** | 2.12 (1.21, 3.71) | 0.009 | 1.68 (0.82, 3.46) | 0.158 | 1.62 (0.78, 3.33) | 0.193 | 1.63 (0.79, 3.37) | 0.182 |
| **35-44** | 1.93 (1.1, 3.39) | 0.022 | 1.72 (0.84, 3.55) | 0.140 | 1.63 (0.79, 3.37) | 0.184 | 1.66 (0.8, 3.42) | 0.172 |
| **45-54** | 2.43 (1.4, 4.23) | 0.002 | 2.43 (1.19, 4.94) | 0.015 | 2.29 (1.12, 4.66) | 0.023 | 2.33 (1.14, 4.74) | 0.020 |
| **55-64** | 3.65 (2.11, 6.33) | <0.001 | 2.49 (1.19, 5.23) | 0.016 | 2.36 (1.13, 4.94) | 0.023 | 2.42 (1.16, 5.08) | 0.019 |
| **≥65** | 5.85 (3.37, 10.15) | <0.001 | 4.54 (2.13, 9.68) | <0.001 | 4.38 (2.06, 9.34) | <0.001 | 4.49 (2.11, 9.57) | <0.001 |
| **Male** | 2.05 (1.8, 2.33) | <0.001 | 2.12 (1.65, 2.72) | <0.001 | 2.17 (1.69, 2.78) | <0.001 | 2.18 (1.7, 2.8) | <0.001 |
| **Non-Hispanic** | 0.98 (0.76, 1.26) | 0.863 | 0.94 (0.62, 1.43) | 0.769 | 0.97 (0.64, 1.47) | 0.885 | 0.96 (0.64, 1.47) | 0.867 |
| **Caucasian** | 1.17 (0.98, 1.38) | 0.075 | 0.92 (0.67, 1.26) | 0.602 | 0.95 (0.7, 1.3) | 0.760 | 0.94 (0.69, 1.29) | 0.717 |
| **Married** | 0.96 (0.84, 1.1) | 0.593 | 1 (0.76, 1.32) | 0.998 | 1 (0.76, 1.32) | 0.991 | 0.99 (0.75, 1.31) | 0.965 |
| **Employed** | 0.68 (0.59, 0.79) | <0.001 | 0.76 (0.57, 1.02) | 0.070 | 0.76 (0.57, 1.02) | 0.070 | 0.77 (0.57, 1.03) | 0.075 |
| **Household Income (ref=<$25,000)** |  |  |  |  |  |  |  |  |
| **$25,000 to $49,999** | 0.87 (0.7, 1.08) | 0.202 | 0.79 (0.53, 1.18) | 0.251 | 0.8 (0.54, 1.2) | 0.285 | 0.8 (0.54, 1.2) | 0.288 |
| **$50,000 to $74,999** | 0.64 (0.51, 0.8) | <0.001 | 0.54 (0.35, 0.84) | 0.007 | 0.55 (0.35, 0.85) | 0.008 | 0.55 (0.35, 0.86) | 0.009 |
| **$75,000 to $99,999** | 0.57 (0.45, 0.73) | <0.001 | 0.5 (0.31, 0.81) | 0.005 | 0.5 (0.31, 0.82) | 0.006 | 0.51 (0.31, 0.83) | 0.006 |
| **≥$100,000** | 0.68 (0.54, 0.84) | 0.001 | 0.79 (0.52, 1.22) | 0.286 | 0.8 (0.52, 1.23) | 0.313 | 0.81 (0.52, 1.24) | 0.323 |
| **Migraine Screen Positive** | 2.69 (2.32, 3.13) | <0.001 |  |  |  |  |  |  |
| **Monthly Headache Days (ref=1 to 4)** |  |  |  |  |  |  |  |  |
| **5 to 9** |  |  | 1.45 (1.08, 1.94) | 0.013 |  |  | 1.38 (1.03, 1.85) | 0.032 |
| **10 to 14** |  |  | 1.17 (0.74, 1.84) | 0.500 |  |  | 1.11 (0.71, 1.75) | 0.645 |
| **15 to 20** |  |  | 1.07 (0.62, 1.84) | 0.806 |  |  | 1.01 (0.59, 1.74) | 0.973 |
| **≥21** |  |  | 1.26 (0.73, 2.17) | 0.401 |  |  | 1.19 (0.69, 2.06) | 0.522 |
| **Headache Pain Intensity (ref=low pain, 1 to 3)** |  |  |  |  |  |  |  |  |
| **Moderate Pain, 4 to 6** |  |  |  |  | 2.35 (0.95, 5.84) | 0.065 | 2.3 (0.92, 5.7) | 0.073 |
| **Severe Pain, 7 to 10** |  |  |  |  | 3.14 (1.28, 7.7) | 0.013 | 2.99 (1.22, 7.35) | 0.017 |

| **Additional File 7C**  **Cardiovascular** | **MAST Overall Population  (N=92,586)** | | **MAST Respondents with Migraine MHD Model**  **(N=15,133)** | | **MAST Respondents with Migraine Headache Pain Intensity Model  (N=15,133)** | | **MAST Respondents with Migraine**  **MHD + Headache Pain Intensity Model**  **(N=15,133)** | |
| --- | --- | --- | --- | --- | --- | --- | --- | --- |
| **Myocardial infarction** | **OR (95%CI)** | **P-Value** | **OR (95%CI)** | **P-Value** | **OR (95%CI)** | **P-Value** | **OR (95%CI)** | **P-Value** |
| **Age (ref=18 -24)** |  |  |  |  |  |  |  |  |
| **25-34** | 0.8 (0.39, 1.62) | 0.527 | 2.98 (0.38, 23.17) | 0.298 | 2.86 (0.37, 22.29) | 0.315 | 2.9 (0.37, 22.57) | 0.309 |
| **35-44** | 1.81 (0.94, 3.51) | 0.077 | 8.77 (1.19, 64.53) | 0.033 | 8.31 (1.13, 61.18) | 0.038 | 8.5 (1.15, 62.52) | 0.036 |
| **45-54** | 3.13 (1.65, 5.95) | <0.001 | 11.07 (1.51, 81) | 0.018 | 10.48 (1.43, 76.65) | 0.021 | 10.73 (1.47, 78.53) | 0.019 |
| **55-64** | 6.09 (3.23, 11.5) | <0.001 | 22.51 (3.08, 164.39) | 0.002 | 20.88 (2.86, 152.53) | 0.003 | 21.87 (2.99, 159.8) | 0.002 |
| **≥65** | 12.17 (6.45, 22.96) | <0.001 | 31.89 (4.32, 235.54) | 0.001 | 30.1 (4.07, 222.42) | 0.001 | 31.32 (4.24, 231.37) | 0.001 |
| **Male** | 3.76 (3.33, 4.24) | <0.001 | 3.68 (2.77, 4.88) | <0.001 | 3.62 (2.73, 4.81) | <0.001 | 3.71 (2.79, 4.93) | <0.001 |
| **Non-Hispanic** | 1.16 (0.9, 1.5) | 0.256 | 1.58 (0.82, 3.02) | 0.170 | 1.6 (0.84, 3.07) | 0.153 | 1.59 (0.83, 3.04) | 0.163 |
| **Caucasian** | 1.18 (1.02, 1.38) | 0.030 | 0.81 (0.56, 1.18) | 0.271 | 0.83 (0.57, 1.2) | 0.316 | 0.82 (0.56, 1.19) | 0.302 |
| **Married** | 1.03 (0.93, 1.16) | 0.545 | 1.17 (0.84, 1.62) | 0.358 | 1.18 (0.85, 1.64) | 0.325 | 1.17 (0.84, 1.62) | 0.356 |
| **Employed** | 0.68 (0.61, 0.77) | <0.001 | 0.58 (0.42, 0.8) | 0.001 | 0.58 (0.42, 0.81) | 0.001 | 0.59 (0.42, 0.81) | 0.001 |
| **Household Income (ref=<$25,000)** |  |  |  |  |  |  |  |  |
| **$25,000 to $49,999** | 0.88 (0.73, 1.06) | 0.182 | 0.68 (0.41, 1.12) | 0.129 | 0.69 (0.42, 1.13) | 0.138 | 0.69 (0.42, 1.14) | 0.145 |
| **$50,000 to $74,999** | 0.79 (0.66, 0.96) | 0.017 | 0.7 (0.42, 1.16) | 0.171 | 0.7 (0.43, 1.16) | 0.169 | 0.71 (0.43, 1.18) | 0.185 |
| **$75,000 to $99,999** | 0.61 (0.5, 0.75) | <0.001 | 0.5 (0.28, 0.88) | 0.016 | 0.5 (0.28, 0.88) | 0.016 | 0.5 (0.29, 0.89) | 0.018 |
| **≥$100,000** | 0.55 (0.46, 0.67) | <0.001 | 0.78 (0.47, 1.3) | 0.340 | 0.77 (0.47, 1.29) | 0.323 | 0.78 (0.47, 1.31) | 0.350 |
| **Migraine Screen Positive** | 1.66 (1.43, 1.93) | <0.001 |  |  |  |  |  |  |
| **Monthly Headache Days (ref=1 to 4)** |  |  |  |  |  |  |  |  |
| **5 to 9** |  |  | 1.72 (1.24, 2.39) | 0.001 |  |  | 1.7 (1.22, 2.36) | 0.002 |
| **10 to 14** |  |  | 1.21 (0.71, 2.06) | 0.492 |  |  | 1.17 (0.69, 2.01) | 0.557 |
| **15 to 20** |  |  | 1.51 (0.88, 2.6) | 0.133 |  |  | 1.47 (0.85, 2.52) | 0.165 |
| **≥21** |  |  | 0.96 (0.49, 1.86) | 0.902 |  |  | 0.93 (0.48, 1.8) | 0.818 |
| **Headache Pain Intensity (ref=low pain, 1 to 3)** |  |  |  |  |  |  |  |  |
| **Moderate Pain, 4 to 6** |  |  |  |  | 0.84 (0.45, 1.55) | 0.578 | 0.8 (0.43, 1.48) | 0.470 |
| **Severe Pain, 7 to 10** |  |  |  |  | 1.12 (0.62, 2.03) | 0.702 | 1.03 (0.57, 1.87) | 0.916 |

| **Additional File 7D**  **Cardiovascular** | **MAST Overall Population  (N=92,586)** | | **MAST Respondents with Migraine MHD Model**  **(N=15,133)** | | **MAST Respondents with Migraine Headache Pain Intensity Model  (N=15,133)** | | **MAST Respondents with Migraine**  **MHD + Headache Pain Intensity Model**  **(N=15,133)** | |
| --- | --- | --- | --- | --- | --- | --- | --- | --- |
| **Hypertension** | OR (95%CI) | P-Value | OR (95%CI) | P-Value | OR (95%CI) | P-Value | OR (95%CI) | P-Value |
| **Age (ref=18 -24)** |  |  |  |  |  |  |  |  |
| **25-34** | 1.87 (1.54, 2.27) | <0.001 | 1.86 (1.41, 2.46) | <0.001 | 1.81 (1.37, 2.39) | <0.001 | 1.83 (1.38, 2.41) | <0.001 |
| **35-44** | 4.42 (3.67, 5.33) | <0.001 | 3.45 (2.63, 4.52) | <0.001 | 3.34 (2.55, 4.38) | <0.001 | 3.36 (2.56, 4.4) | <0.001 |
| **45-54** | 10.07 (8.38, 12.11) | <0.001 | 7.04 (5.39, 9.21) | <0.001 | 6.83 (5.22, 8.93) | <0.001 | 6.85 (5.24, 8.96) | <0.001 |
| **55-64** | 16.33 (13.58, 19.64) | <0.001 | 9.62 (7.31, 12.67) | <0.001 | 9.29 (7.05, 12.23) | <0.001 | 9.41 (7.15, 12.39) | <0.001 |
| **≥65** | 24.53 (20.38, 29.53) | <0.001 | 11.53 (8.63, 15.42) | <0.001 | 11.27 (8.43, 15.06) | <0.001 | 11.41 (8.53, 15.26) | <0.001 |
| **Male** | 1.46 (1.41, 1.51) | <0.001 | 1.96 (1.8, 2.14) | <0.001 | 1.97 (1.81, 2.15) | <0.001 | 1.99 (1.82, 2.17) | <0.001 |
| **Non-Hispanic** | 1.16 (1.08, 1.25) | <0.001 | 1.07 (0.92, 1.23) | 0.382 | 1.09 (0.94, 1.25) | 0.264 | 1.08 (0.94, 1.25) | 0.294 |
| **Caucasian** | 0.74 (0.71, 0.77) | <0.001 | 0.69 (0.62, 0.76) | <0.001 | 0.71 (0.64, 0.78) | <0.001 | 0.7 (0.63, 0.77) | <0.001 |
| **Married** | 0.96 (0.93, 1) | 0.036 | 1.12 (1.02, 1.23) | 0.017 | 1.12 (1.02, 1.23) | 0.016 | 1.12 (1.02, 1.22) | 0.019 |
| **Employed** | 0.78 (0.75, 0.81) | <0.001 | 0.77 (0.69, 0.85) | <0.001 | 0.76 (0.69, 0.84) | <0.001 | 0.77 (0.7, 0.85) | <0.001 |
| **Household Income** |  |  |  |  |  |  |  |  |
| **$25,000 to $49,999 (ref: <$25,000)** | 0.97 (0.91, 1.04) | 0.376 | 0.96 (0.82, 1.11) | 0.553 | 0.95 (0.82, 1.11) | 0.532 | 0.96 (0.83, 1.12) | 0.638 |
| **$50,000 to $74,999** | 0.92 (0.86, 0.99) | 0.019 | 0.88 (0.75, 1.02) | 0.089 | 0.87 (0.74, 1.01) | 0.069 | 0.88 (0.76, 1.03) | 0.116 |
| **$75,000 to $99,999** | 0.86 (0.8, 0.92) | <0.001 | 0.89 (0.76, 1.05) | 0.173 | 0.88 (0.75, 1.04) | 0.133 | 0.9 (0.77, 1.06) | 0.221 |
| **more than $100,000** | 0.8 (0.75, 0.85) | <0.001 | 0.74 (0.63, 0.87) | <0.001 | 0.73 (0.62, 0.85) | <0.001 | 0.75 (0.64, 0.88) | <0.001 |
| **Migraine Screen Positive** | 1.51 (1.44, 1.58) | <0.001 |  |  |  |  |  |  |
| **Monthly HA days (ref= 1 to 4 MHD)** |  |  |  |  |  |  |  |  |
| **5 to 9** |  |  | 1.15 (1.04, 1.28) | 0.009 |  |  | 1.13 (1.01, 1.25) | 0.029 |
| **10 to 14** |  |  | 1.34 (1.15, 1.55) | <0.001 |  |  | 1.3 (1.12, 1.51) | 0.001 |
| **15 to 20** |  |  | 1.52 (1.29, 1.8) | <0.001 |  |  | 1.47 (1.24, 1.75) | <0.001 |
| **21 or more** |  |  | 1.37 (1.13, 1.66) | 0.001 |  |  | 1.33 (1.1, 1.61) | 0.003 |
| **Headache Pain Intensity (ref=low pain, 1 to 3)** |  |  |  |  |  |  |  |  |
| **Moderate Pain, 4 to 6** |  |  |  |  | 1.12 (0.9, 1.39) | 0.296 | 1.11 (0.89, 1.37) | 0.348 |
| **Severe Pain, 7 to 10** |  |  |  |  | 1.38 (1.12, 1.7) | 0.003 | 1.33 (1.07, 1.64) | 0.009 |

| **Additional File 7E**  **Cardiovascular** | **MAST Overall Population  (N=92,586)** | | | **MAST Respondents with Migraine MHD Model**  **(N=15,133)** | | | **MAST Respondents with Migraine Headache Pain Intensity Model  (N=15,133)** | | | **MAST Respondents with Migraine**  **MHD + Headache Pain Intensity Model**  **(N=15,133)** | | |
| --- | --- | --- | --- | --- | --- | --- | --- | --- | --- | --- | --- | --- |
| **High Cholesterol** | **OR (95%CI)** | **P-Value** | **OR (95%CI)** | | **P-Value** | **OR (95%CI)** | | **P-Value** | **OR (95%CI)** | | **P-Value** |  |
| **Age (ref=18 -24)** |  |  |  | |  |  | |  |  | |  |  |
| **35-44** | 3.55 (2.98, 4.24) | <0.001 | 3.07 (2.34, 4.02) | | <0.001 | 2.99 (2.28, 3.92) | | <0.001 | 3.01 (2.3, 3.95) | | <0.001 |  |
| **45-54** | 7.62 (6.41, 9.07) | <0.001 | 6.79 (5.2, 8.88) | | <0.001 | 6.61 (5.06, 8.64) | | <0.001 | 6.67 (5.1, 8.72) | | <0.001 |  |
| **55-64** | 13.38 (11.25, 15.92) | <0.001 | 11 (8.36, 14.47) | | <0.001 | 10.64 (8.08, 13.99) | | <0.001 | 10.83 (8.23, 14.26) | | <0.001 |  |
| **≥65** | 19.12 (16.05, 22.77) | <0.001 | 15.26 (11.42, 20.38) | | <0.001 | 14.9 (11.15, 19.9) | | <0.001 | 15.15 (11.33, 20.24) | | <0.001 |  |
| **Male** | 1.61 (1.55, 1.66) | <0.001 | 2.08 (1.9, 2.26) | | <0.001 | 2.08 (1.9, 2.27) | | <0.001 | 2.1 (1.92, 2.29) | | <0.001 |  |
| **Non-Hispanic** | 1 (0.94, 1.07) | 0.975 | 1.02 (0.88, 1.18) | | 0.765 | 1.04 (0.9, 1.2) | | 0.619 | 1.03 (0.89, 1.19) | | 0.670 |  |
| **Caucasian** | 1.04 (1, 1.09) | 0.057 | 0.87 (0.79, 0.97) | | 0.012 | 0.89 (0.8, 0.99) | | 0.031 | 0.88 (0.8, 0.98) | | 0.021 |  |
| **Married** | 1.05 (1.01, 1.09) | 0.008 | 1.12 (1.02, 1.23) | | 0.016 | 1.12 (1.02, 1.23) | | 0.016 | 1.12 (1.02, 1.23) | | 0.018 |  |
| **Employed** | 0.75 (0.72, 0.78) | <0.001 | 0.77 (0.7, 0.85) | | <0.001 | 0.76 (0.69, 0.84) | | <0.001 | 0.77 (0.7, 0.85) | | <0.001 |  |
| **Household Income** |  |  |  | |  |  | |  |  | |  |  |
| **$25,000 to $49,999 (ref: <$25,000)** | 1 (0.94, 1.07) | 0.907 | 0.86 (0.73, 1) | | 0.044 | 0.86 (0.73, 1) | | 0.045 | 0.86 (0.74, 1) | | 0.054 |  |
| **$50,000 to $74,999** | 1.09 (1.02, 1.16) | 0.016 | 0.89 (0.77, 1.05) | | 0.161 | 0.89 (0.76, 1.04) | | 0.145 | 0.9 (0.77, 1.05) | | 0.191 |  |
| **$75,000 to $99,999** | 1.08 (1.01, 1.16) | 0.031 | 0.98 (0.83, 1.15) | | 0.795 | 0.97 (0.83, 1.15) | | 0.748 | 0.99 (0.84, 1.16) | | 0.873 |  |
| **more than $100,000** | 1.11 (1.04, 1.18) | 0.003 | 1.01 (0.86, 1.18) | | 0.896 | 1 (0.86, 1.17) | | 0.972 | 1.02 (0.87, 1.19) | | 0.824 |  |
| **Migraine Screen Positive** | 1.61 (1.53, 1.68) | <0.001 |  | |  |  | |  |  | |  |  |
| **Monthly HA days (ref= 1 to 4 MHD)** |  |  |  | |  |  | |  |  | |  |  |
| **5 to 9** |  |  | 1.23 (1.11, 1.37) | | <0.001 |  | |  | 1.21 (1.09, 1.35) | | <0.001 |  |
| **10 to 14** |  |  | 1.18 (1.01, 1.37) | | 0.037 |  | |  | 1.15 (0.99, 1.34) | | 0.065 |  |
| **15 to 20** |  |  | 1.35 (1.14, 1.61) | | 0.001 |  | |  | 1.32 (1.11, 1.57) | | 0.002 |  |
| **21 or more** |  |  | 1.2 (0.99, 1.46) | | 0.064 |  | |  | 1.18 (0.97, 1.43) | | 0.103 |  |
| **Headache pain intensity (ref=low pain, 1 to 3)** |  |  |  | |  |  | |  |  | |  |  |
| **Moderate Pain, 4 to 6** |  |  |  | |  | 1.1 (0.89, 1.36) | | 0.353 | 1.09 (0.88, 1.34) | | 0.436 |  |
| **Severe Pain, 7 to 10** |  |  |  | |  | 1.28 (1.04, 1.57) | | 0.019 | 1.23 (1, 1.52) | | 0.050 |  |

| **Additional File 7F**  **Neurologic** | **MAST Overall Population  (N=92,586)** | | | **MAST Respondents with Migraine MHD Model**  **(N=15,133)** | | | **MAST Respondents with Migraine  Headache Pain Intensity Model  (N=15,133)** | | | **MAST Respondents with Migraine**  **MHD + Headache Pain Intensity Model**  **(N=15,133)** | |
| --- | --- | --- | --- | --- | --- | --- | --- | --- | --- | --- | --- |
| **Epilepsy** | **OR (95%CI)** | **P-Value** | **OR (95%CI)** | | **P-Value** | **OR (95%CI)** | | **P-Value** | **OR (95%CI)** | | **P-Value** |
| **Age (ref=18 -24)** |  |  |  | |  |  | |  |  | |  |
| **25-34** | 1.28 (0.86, 1.89) | 0.224 | 1.38 (0.76, 2.51) | | 0.290 | 1.34 (0.74, 2.43) | | 0.339 | 1.33 (0.73, 2.42) | | 0.347 |
| **35-44** | 1.31 (0.88, 1.94) | 0.185 | 1.43 (0.79, 2.61) | | 0.239 | 1.37 (0.75, 2.51) | | 0.302 | 1.36 (0.75, 2.49) | | 0.314 |
| **45-54** | 1.17 (0.79, 1.74) | 0.437 | 1.37 (0.74, 2.51) | | 0.313 | 1.32 (0.72, 2.43) | | 0.374 | 1.3 (0.7, 2.38) | | 0.406 |
| **55-64** | 0.95 (0.63, 1.43) | 0.804 | 1.01 (0.51, 1.98) | | 0.976 | 0.97 (0.49, 1.91) | | 0.928 | 0.96 (0.49, 1.89) | | 0.909 |
| **≥65** | 0.46 (0.3, 0.72) | 0.001 | 0.64 (0.28, 1.48) | | 0.294 | 0.62 (0.27, 1.43) | | 0.259 | 0.62 (0.27, 1.43) | | 0.263 |
| **Male** | 1.33 (1.13, 1.56) | 0.001 | 1.28 (0.95, 1.73) | | 0.101 | 1.29 (0.96, 1.74) | | 0.091 | 1.29 (0.96, 1.74) | | 0.089 |
| **Non-Hispanic** | 0.84 (0.66, 1.08) | 0.172 | 0.9 (0.6, 1.35) | | 0.626 | 0.93 (0.62, 1.4) | | 0.741 | 0.92 (0.62, 1.38) | | 0.701 |
| **Caucasian** | 1.15 (0.95, 1.4) | 0.141 | 0.74 (0.55, 1) | | 0.050 | 0.77 (0.57, 1.04) | | 0.087 | 0.76 (0.56, 1.03) | | 0.073 |
| **Married** | 0.94 (0.79, 1.12) | 0.465 | 1.23 (0.91, 1.66) | | 0.170 | 1.23 (0.91, 1.65) | | 0.179 | 1.22 (0.91, 1.65) | | 0.186 |
| **Employed** | 0.6 (0.5, 0.72) | <0.001 | 0.64 (0.47, 0.88) | | 0.005 | 0.63 (0.46, 0.86) | | 0.003 | 0.65 (0.48, 0.89) | | 0.007 |
| **Household Income (ref=<$25,000)** |  |  |  | |  |  | |  |  | |  |
| **$25,000 to $49,999** | 0.6 (0.46, 0.77) | <0.001 | 0.51 (0.34, 0.76) | | 0.001 | 0.51 (0.34, 0.76) | | 0.001 | 0.52 (0.35, 0.78) | | 0.001 |
| **$50,000 to $74,999** | 0.59 (0.46, 0.77) | <0.001 | 0.52 (0.34, 0.8) | | 0.003 | 0.52 (0.34, 0.79) | | 0.002 | 0.53 (0.35, 0.81) | | 0.003 |
| **$75,000 to $99,999** | 0.51 (0.38, 0.68) | <0.001 | 0.41 (0.25, 0.67) | | <0.001 | 0.4 (0.25, 0.65) | | <0.001 | 0.42 (0.26, 0.68) | | <0.001 |
| **≥$100,000** | 0.48 (0.37, 0.63) | <0.001 | 0.39 (0.25, 0.62) | | <0.001 | 0.38 (0.24, 0.61) | | <0.001 | 0.4 (0.25, 0.64) | | <0.001 |
| **Migraine Screen Positive** | 2.33 (1.96, 2.76) | <0.001 | 1.38 (0.76, 2.51) | | 0.290 | 1.34 (0.74, 2.43) | | 0.339 | 1.33 (0.73, 2.42) | | 0.347 |
| **Monthly Headache Days (ref=1 to 4)** |  |  |  | |  |  | |  |  | |  |
| **5 to 9** |  |  | 1.04 (0.73, 1.49) | | 0.812 |  | |  | 1.01 (0.7, 1.45) | | 0.958 |
| **10 to 14** |  |  | 1.54 (1, 2.39) | | 0.052 |  | |  | 1.47 (0.95, 2.29) | | 0.083 |
| **15 to 20** |  |  | 2.06 (1.31, 3.23) | | 0.002 |  | |  | 1.94 (1.23, 3.04) | | 0.004 |
| **≥21** |  |  | 1.52 (0.86, 2.68) | | 0.145 |  | |  | 1.44 (0.82, 2.55) | | 0.203 |
| **Headache Pain Intensity (ref=low pain, 1 to 3)** |  |  |  | |  |  | |  |  | |  |
| **Moderate Pain, 4 to 6** |  |  |  | |  | 0.8 (0.38, 1.68) | | 0.555 | 0.8 (0.38, 1.68) | | 0.549 |
| **Severe Pain, 7 to 10** |  |  |  | |  | 1.22 (0.59, 2.5) | | 0.594 | 1.15 (0.56, 2.38) | | 0.701 |

| **Additional File 7G**  **Neurologic** | **MAST Overall Population  (N=92,586)** | | | **MAST Respondents with Migraine MHD Model**  **(N=15,133)** | | | **MAST Respondents with Migraine Headache Pain Intensity Model  (N=15,133)** | | | **MAST Respondents with Migraine**  **MHD + Headache Pain Intensity Model**  **(N=15,133)** | |
| --- | --- | --- | --- | --- | --- | --- | --- | --- | --- | --- | --- |
| **Stroke or TIA** | **OR (95%CI)** | **P-Value** | **OR (95%CI)** | | **P-Value** | **OR (95%CI)** | | **P-Value** | **OR (95%CI)** | | **P-Value** |
| **Age (ref=18 -24)** |  |  |  | |  |  | |  |  | |  |
| **25-34** | 1.54 (0.68, 3.45) | 0.300 | 1.76 (0.51, 6.08) | | 0.371 | 1.68 (0.49, 5.82) | | 0.409 | 1.71 (0.5, 5.91) | | 0.396 |
| **35-44** | 3.3 (1.52, 7.17) | 0.003 | 4.7 (1.45, 15.27) | | 0.010 | 4.48 (1.38, 14.58) | | 0.013 | 4.53 (1.39, 14.73) | | 0.012 |
| **45-54** | 6.88 (3.23, 14.69) | 0.000 | 8.14 (2.54, 26.1) | | 0.000 | 7.82 (2.44, 25.08) | | 0.001 | 7.84 (2.44, 25.14) | | 0.001 |
| **55-64** | 10.76 (5.06, 22.88) | 0.000 | 10.92 (3.38, 35.3) | | 0.000 | 10.2 (3.15, 33) | | 0.000 | 10.48 (3.24, 33.9) | | 0.000 |
| **≥65** | 16.44 (7.72, 35.01) | 0.000 | 14.84 (4.53, 48.64) | | 0.000 | 14.06 (4.29, 46.12) | | 0.000 | 14.41 (4.39, 47.27) | | 0.000 |
| **Male** | 1.47 (1.31, 1.65) | 0.000 | 1.73 (1.34, 2.24) | | 0.000 | 1.71 (1.32, 2.21) | | 0.000 | 1.73 (1.34, 2.24) | | 0.000 |
| **Non-Hispanic** | 1.03 (0.8, 1.34) | 0.800 | 0.92 (0.59, 1.45) | | 0.721 | 0.94 (0.6, 1.48) | | 0.791 | 0.93 (0.59, 1.46) | | 0.737 |
| **Caucasian** | 0.9 (0.77, 1.04) | 0.140 | 0.76 (0.55, 1.04) | | 0.084 | 0.77 (0.56, 1.05) | | 0.102 | 0.77 (0.56, 1.05) | | 0.097 |
| **Married** | 0.96 (0.85, 1.09) | 0.560 | 0.99 (0.75, 1.32) | | 0.958 | 1 (0.76, 1.33) | | 0.986 | 0.99 (0.75, 1.32) | | 0.970 |
| **Employed** | 0.51 (0.45, 0.58) | 0.000 | 0.5 (0.37, 0.66) | | 0.000 | 0.49 (0.37, 0.65) | | 0.000 | 0.5 (0.38, 0.67) | | 0.000 |
| **Household Income (ref=<$25,000)** |  |  |  | |  |  | |  |  | |  |
| **$25,000 to $49,999** | 0.78 (0.64, 0.95) | 0.012 | 0.64 (0.42, 0.96) | | 0.032 | 0.63 (0.42, 0.95) | | 0.028 | 0.64 (0.42, 0.97) | | 0.036 |
| **$50,000 to $74,999** | 0.66 (0.54, 0.81) | 0.000 | 0.8 (0.53, 1.21) | | 0.283 | 0.78 (0.51, 1.17) | | 0.227 | 0.8 (0.53, 1.21) | | 0.298 |
| **$75,000 to $99,999** | 0.61 (0.49, 0.75) | 0.000 | 0.68 (0.43, 1.08) | | 0.101 | 0.66 (0.42, 1.04) | | 0.075 | 0.69 (0.43, 1.09) | | 0.111 |
| **≥$100,000** | 0.6 (0.49, 0.73) | 0.000 | 0.6 (0.38, 0.94) | | 0.025 | 0.57 (0.36, 0.89) | | 0.013 | 0.6 (0.38, 0.94) | | 0.025 |
| **Migraine Screen Positive** | 2.18 (1.89, 2.53) | 0.000 | 1.76 (0.51, 6.08) | | 0.371 | 1.68 (0.49, 5.82) | | 0.409 | 1.71 (0.5, 5.91) | | 0.396 |
| **Monthly Headache Days (ref=1 to 4)** |  |  |  | |  |  | |  |  | |  |
| **5 to 9** |  |  | 1.54 (1.13, 2.1) | | 0.006 |  | |  | 1.54 (1.13, 2.1) | | 0.007 |
| **10 to 14** |  |  | 1.64 (1.08, 2.51) | | 0.021 |  | |  | 1.62 (1.06, 2.48) | | 0.026 |
| **15 to 20** |  |  | 1.56 (0.97, 2.51) | | 0.065 |  | |  | 1.52 (0.95, 2.45) | | 0.082 |
| **≥21** |  |  | 1.99 (1.25, 3.15) | | 0.003 |  | |  | 1.94 (1.22, 3.07) | | 0.005 |
| **Headache Pain Intensity (ref=low pain, 1 to 3)** |  |  |  | |  |  | |  |  | |  |
| **Moderate Pain, 4 to 6** |  |  |  | |  | 0.6 (0.35, 1.02) | | 0.061 | 0.57 (0.33, 0.98) | | 0.042 |
| **Severe Pain, 7 to 10** |  |  |  | |  | 0.84 (0.5, 1.41) | | 0.511 | 0.76 (0.45, 1.28) | | 0.303 |

| **Additional File 7H**  **General Medical** | **MAST Overall Population  (N=92,586)** | | **MAST Respondents with Migraine MHD Model**  **(N=15,133)** | | | **MAST Respondents with Migraine Headache Pain Intensity Model  (N=15,133)** | | **MAST Respondents with Migraine**  **MHD + Headache Pain Intensity Model**  **(N=15,133)** | |
| --- | --- | --- | --- | --- | --- | --- | --- | --- | --- |
| **Gastric Ulcer/GI Bleeding** | **OR (95%CI)** | **P-Value** | **OR (95%CI)** | **P-Value** | **OR (95%CI)** | | **P-Value** | **OR (95%CI)** | **P-Value** |
| **Age (ref=18 -24)** |  |  |  |  |  | |  |  |  |
| **25-34** | 1 (0.75, 1.33) | 0.987 | 1.06 (0.71, 1.57) | 0.790 | 1.03 (0.69, 1.52) | | 0.897 | 1.04 (0.7, 1.55) | 0.841 |
| **35-44** | 1.24 (0.93, 1.64) | 0.141 | 1.48 (1.01, 2.18) | 0.046 | 1.44 (0.98, 2.12) | | 0.064 | 1.45 (0.99, 2.14) | 0.058 |
| **45-54** | 1.54 (1.17, 2.03) | 0.002 | 1.73 (1.18, 2.54) | 0.005 | 1.69 (1.15, 2.49) | | 0.007 | 1.7 (1.16, 2.49) | 0.007 |
| **55-64** | 1.61 (1.22, 2.13) | 0.001 | 1.61 (1.07, 2.43) | 0.023 | 1.57 (1.04, 2.36) | | 0.032 | 1.6 (1.06, 2.41) | 0.026 |
| **≥65** | 2.12 (1.59, 2.81) | <0.001 | 2.19 (1.41, 3.4) | <0.001 | 2.15 (1.39, 3.33) | | 0.001 | 2.19 (1.41, 3.39) | <0.001 |
| **Male** | 1.13 (1.03, 1.25) | 0.009 | 1.29 (1.09, 1.54) | 0.003 | 1.31 (1.11, 1.55) | | 0.002 | 1.32 (1.11, 1.56) | 0.002 |
| **Non-Hispanic** | 0.86 (0.73, 1.02) | 0.079 | 0.77 (0.6, 0.98) | 0.037 | 0.79 (0.61, 1.01) | | 0.059 | 0.78 (0.61, 1) | 0.049 |
| **Caucasian** | 1.16 (1.03, 1.31) | 0.014 | 1.19 (0.97, 1.47) | 0.097 | 1.23 (1, 1.52) | | 0.051 | 1.21 (0.98, 1.49) | 0.074 |
| **Married** | 0.9 (0.82, 1) | 0.052 | 0.95 (0.79, 1.13) | 0.557 | 0.95 (0.8, 1.14) | | 0.598 | 0.94 (0.79, 1.13) | 0.518 |
| **Employed** | 0.77 (0.69, 0.86) | <0.001 | 0.87 (0.72, 1.06) | 0.166 | 0.84 (0.69, 1.02) | | 0.077 | 0.87 (0.72, 1.06) | 0.166 |
| **Household Income (ref=<$25,000)** |  |  |  |  |  | |  |  |  |
| **$25,000 to $49,999** | 1.15 (0.97, 1.38) | 0.113 | 0.87 (0.66, 1.15) | 0.328 | 0.86 (0.66, 1.13) | | 0.286 | 0.88 (0.67, 1.16) | 0.362 |
| **$50,000 to $74,999** | 1.03 (0.86, 1.23) | 0.771 | 0.7 (0.52, 0.93) | 0.016 | 0.68 (0.51, 0.91) | | 0.009 | 0.7 (0.52, 0.94) | 0.018 |
| **$75,000 to $99,999** | 0.99 (0.82, 1.2) | 0.932 | 0.84 (0.62, 1.14) | 0.272 | 0.81 (0.6, 1.1) | | 0.180 | 0.85 (0.63, 1.16) | 0.310 |
| **≥$100,000** | 1.18 (0.99, 1.41) | 0.071 | 1 (0.75, 1.33) | 0.994 | 0.96 (0.72, 1.28) | | 0.779 | 1.01 (0.76, 1.35) | 0.934 |
| **Migraine Screen Positive** | 3.11 (2.81, 3.45) | <0.001 |  |  |  | |  |  |  |
| **Monthly Headache Days (ref=1 to 4)** |  |  |  |  |  | |  |  |  |
| **5 to 9** |  |  | 1.27 (1.04, 1.56) | 0.020 |  | |  | 1.24 (1.01, 1.52) | 0.040 |
| **10 to 14** |  |  | 1.71 (1.32, 2.23) | <0.001 |  | |  | 1.67 (1.28, 2.18) | <0.001 |
| **15 to 20** |  |  | 1.66 (1.22, 2.26) | 0.001 |  | |  | 1.62 (1.19, 2.2) | 0.002 |
| **≥21** |  |  | 2.27 (1.68, 3.07) | <0.001 |  | |  | 2.21 (1.63, 2.99) | <0.001 |
| **Headache Pain Intensity (ref=low pain, 1 to 3)** |  |  |  |  |  | |  |  |  |
| **Moderate Pain, 4 to 6** |  |  |  |  | 2.13 (1.21, 3.75) | | 0.009 | 2.09 (1.18, 3.69) | 0.011 |
| **Severe Pain, 7 to 10** |  |  |  |  | 2.49 (1.42, 4.37) | | 0.001 | 2.31 (1.32, 4.05) | 0.004 |

| **Additional File 7I**  **General Medical** | **MAST Overall Population  (N=92,586)** | | **MAST Respondents with Migraine MHD Model**  **(N=15,133)** | | **MAST Respondents with Migraine  Headache Pain Intensity Model  (N=15,133)** | | **MAST Respondents with Migraine**  **MHD + Headache Pain Intensity Model**  **(N=15,133)** | |
| --- | --- | --- | --- | --- | --- | --- | --- | --- |
| **Kidney Disease** | **OR (95%CI)** | **P-Value** | **OR (95%CI)** | **P-Value** | **OR (95%CI)** | **P-Value** | **OR (95%CI)** | **P-Value** |
| **Age (ref=18 -24)** |  |  |  |  |  |  |  |  |
| **25-34** | 1.35 (0.88, 2.09) | 0.171 | 2.07 (0.87, 4.94) | 0.100 | 1.97 (0.83, 4.69) | 0.127 | 1.97 (0.83, 4.7) | 0.125 |
| **35-44** | 1.55 (1.01, 2.38) | 0.047 | 2.02 (0.84, 4.82) | 0.114 | 1.9 (0.79, 4.54) | 0.149 | 1.89 (0.79, 4.51) | 0.154 |
| **45-54** | 1.97 (1.3, 3.01) | 0.002 | 2.97 (1.26, 6.99) | 0.013 | 2.79 (1.19, 6.58) | 0.019 | 2.77 (1.18, 6.52) | 0.020 |
| **55-64** | 2.95 (1.94, 4.47) | <0.001 | 4.17 (1.76, 9.92) | 0.001 | 3.93 (1.65, 9.35) | 0.002 | 3.9 (1.64, 9.29) | 0.002 |
| **≥65** | 4.28 (2.82, 6.49) | <0.001 | 6.13 (2.53, 14.83) | <0.001 | 5.92 (2.44, 14.32) | <0.001 | 5.87 (2.42, 14.21) | <0.001 |
| **Male** | 1.49 (1.34, 1.66) | <0.001 | 1.9 (1.47, 2.47) | <0.001 | 1.94 (1.5, 2.52) | <0.001 | 1.93 (1.49, 2.51) | <0.001 |
| **Non-Hispanic** | 0.96 (0.78, 1.19) | 0.734 | 0.9 (0.58, 1.41) | 0.653 | 0.92 (0.59, 1.44) | 0.720 | 0.92 (0.59, 1.44) | 0.729 |
| **Caucasian** | 0.81 (0.71, 0.92) | 0.001 | 0.81 (0.59, 1.12) | 0.203 | 0.83 (0.6, 1.15) | 0.265 | 0.83 (0.61, 1.15) | 0.270 |
| **Married** | 0.96 (0.85, 1.08) | 0.469 | 0.83 (0.63, 1.11) | 0.216 | 0.83 (0.63, 1.11) | 0.218 | 0.83 (0.62, 1.11) | 0.210 |
| **Employed** | 0.62 (0.55, 0.7) | <0.001 | 0.53 (0.39, 0.71) | <0.001 | 0.53 (0.39, 0.72) | <0.001 | 0.54 (0.4, 0.73) | <0.001 |
| **Household Income (ref=<$25,000)** |  |  |  |  |  |  |  |  |
| **$25,000 to $49,999** | 0.71 (0.59, 0.85) | <0.001 | 1.01 (0.65, 1.59) | 0.959 | 1.02 (0.65, 1.61) | 0.916 | 1.03 (0.66, 1.62) | 0.882 |
| **$50,000 to $74,999** | 0.62 (0.52, 0.75) | <0.001 | 0.83 (0.51, 1.34) | 0.445 | 0.83 (0.51, 1.35) | 0.455 | 0.84 (0.52, 1.37) | 0.487 |
| **$75,000 to $99,999** | 0.63 (0.52, 0.77) | <0.001 | 1.19 (0.73, 1.94) | 0.486 | 1.19 (0.73, 1.95) | 0.477 | 1.21 (0.74, 1.98) | 0.435 |
| **≥$100,000** | 0.61 (0.51, 0.73) | <0.001 | 1.16 (0.72, 1.87) | 0.537 | 1.16 (0.72, 1.86) | 0.550 | 1.18 (0.73, 1.9) | 0.501 |
| **Migraine Screen Positive** | 1.48 (1.28, 1.71) | <0.001 |  |  |  |  |  |  |
| **Monthly Headache Days (ref=1 to 4)** |  |  |  |  |  |  |  |  |
| **5 to 9** |  |  | 0.99 (0.71, 1.39) | 0.954 |  |  | 0.95 (0.68, 1.34) | 0.778 |
| **10 to 14** |  |  | 1.09 (0.68, 1.75) | 0.724 |  |  | 1.03 (0.64, 1.66) | 0.890 |
| **15 to 20** |  |  | 1.06 (0.62, 1.83) | 0.822 |  |  | 1 (0.58, 1.72) | 1.000 |
| **≥21** |  |  | 1.61 (0.99, 2.64) | 0.057 |  |  | 1.52 (0.93, 2.49) | 0.095 |
| **Headache Pain Intensity (ref=low pain, 1 to 3)** |  |  |  |  |  |  |  |  |
| **Moderate Pain, 4 to 6** |  |  |  |  | 0.7 (0.38, 1.28) | 0.243 | 0.7 (0.38, 1.28) | 0.247 |
| **Severe Pain, 7 to 10** |  |  |  |  | 1.15 (0.64, 2.05) | 0.637 | 1.14 (0.64, 2.04) | 0.653 |

| **Additional File 7J**  **General Medical** | **MAST Overall Population  (N=92,586)** | | **MAST Respondents with Migraine MHD Model**  **(N=15,133)** | | **MAST Respondents with Migraine Headache Pain Intensity Model  (N=15,133)** | | **MAST Respondents with Migraine**  **MHD + Headache Pain Intensity Model**  **(N=15,133)** | |
| --- | --- | --- | --- | --- | --- | --- | --- | --- |
| **Vitamin D Deficiency** | **OR (95%CI)** | **P-Value** | **OR (95%CI)** | **P-Value** | **OR (95%CI)** | **P-Value** | **OR (95%CI)** | **P-Value** |
| **Age (ref=18 -24)** |  |  |  |  |  |  |  |  |
| **25-34** | 1.33 (1.17, 1.5) | <0.001 | 1.28 (1.06, 1.53) | 0.010 | 1.23 (1.02, 1.48) | 0.028 | 1.24 (1.03, 1.49) | 0.023 |
| **35-44** | 1.82 (1.62, 2.06) | <0.001 | 1.95 (1.63, 2.34) | <0.001 | 1.86 (1.55, 2.24) | <0.001 | 1.87 (1.56, 2.25) | <0.001 |
| **45-54** | 2.13 (1.89, 2.4) | <0.001 | 2.29 (1.91, 2.75) | <0.001 | 2.19 (1.82, 2.64) | <0.001 | 2.19 (1.82, 2.63) | <0.001 |
| **55-64** | 2.09 (1.85, 2.36) | <0.001 | 2.08 (1.7, 2.53) | <0.001 | 1.98 (1.63, 2.42) | <0.001 | 2 (1.64, 2.44) | <0.001 |
| **≥65** | 1.73 (1.53, 1.96) | <0.001 | 1.8 (1.44, 2.25) | <0.001 | 1.75 (1.4, 2.19) | <0.001 | 1.76 (1.4, 2.21) | <0.001 |
| **Male** | 0.54 (0.51, 0.56) | <0.001 | 0.57 (0.52, 0.62) | <0.001 | 0.58 (0.52, 0.63) | <0.001 | 0.58 (0.52, 0.63) | <0.001 |
| **Non-Hispanic** | 0.8 (0.75, 0.86) | <0.001 | 0.83 (0.73, 0.94) | 0.004 | 0.85 (0.75, 0.97) | 0.013 | 0.85 (0.75, 0.96) | 0.011 |
| **Caucasian** | 0.73 (0.7, 0.76) | <0.001 | 0.77 (0.71, 0.85) | <0.001 | 0.8 (0.73, 0.88) | <0.001 | 0.79 (0.72, 0.87) | <0.001 |
| **Married** | 0.99 (0.94, 1.03) | 0.510 | 0.9 (0.83, 0.98) | 0.020 | 0.9 (0.82, 0.98) | 0.018 | 0.9 (0.82, 0.98) | 0.015 |
| **Employed** | 0.92 (0.88, 0.97) | 0.001 | 0.9 (0.81, 0.99) | 0.029 | 0.89 (0.8, 0.98) | 0.014 | 0.9 (0.82, 1) | 0.041 |
| **Household Income (ref=<$25,000)** |  |  |  |  |  |  |  |  |
| **$25,000 to $49,999** | 0.95 (0.88, 1.03) | 0.218 | 0.9 (0.78, 1.03) | 0.138 | 0.9 (0.78, 1.04) | 0.141 | 0.91 (0.79, 1.05) | 0.194 |
| **$50,000 to $74,999** | 0.98 (0.91, 1.06) | 0.681 | 0.95 (0.82, 1.1) | 0.482 | 0.95 (0.82, 1.09) | 0.443 | 0.96 (0.83, 1.11) | 0.606 |
| **$75,000 to $99,999** | 0.98 (0.91, 1.07) | 0.688 | 1.01 (0.86, 1.17) | 0.939 | 1 (0.86, 1.16) | 0.981 | 1.03 (0.88, 1.2) | 0.749 |
| **≥$100,000** | 1.04 (0.96, 1.12) | 0.333 | 1.07 (0.92, 1.24) | 0.377 | 1.06 (0.91, 1.22) | 0.454 | 1.09 (0.94, 1.26) | 0.256 |
| **Migraine Screen Positive** | 2 (1.91, 2.1) | <0.001 |  |  |  |  |  |  |
| **Monthly Headache Days (ref=1 to 4)** |  |  |  |  |  |  |  |  |
| **5 to 9** |  |  | 1.11 (1, 1.23) | 0.042 |  |  | 1.08 (0.97, 1.19) | 0.159 |
| **10 to 14** |  |  | 1.4 (1.22, 1.61) | <0.001 |  |  | 1.35 (1.17, 1.56) | <0.001 |
| **15 to 20** |  |  | 1.35 (1.15, 1.6) | <0.001 |  |  | 1.29 (1.1, 1.53) | 0.002 |
| **≥21** |  |  | 1.74 (1.45, 2.08) | <0.001 |  |  | 1.66 (1.39, 1.99) | <0.001 |
| **Headache Pain Intensity (ref=low pain, 1 to 3)** |  |  |  |  |  |  |  |  |
| **Moderate Pain, 4 to 6** |  |  |  |  | 1.23 (0.98, 1.55) | 0.078 | 1.22 (0.97, 1.54) | 0.090 |
| **Severe Pain, 7 to 10** |  |  |  |  | 1.64 (1.31, 2.06) | <0.001 | 1.58 (1.26, 1.99) | <0.001 |

| **Additional File 7K**  **General Medical** | **MAST Overall Population  (N=92,586)** | | **MAST Respondents with Migraine MHD Model**  **(N=15,133)** | | **MAST Respondents with Migraine Headache Pain Intensity Model  (N=15,133)** | | **MAST Respondents with Migraine**  **MHD + Headache Pain Intensity Model**  **(N=15,133)** | |
| --- | --- | --- | --- | --- | --- | --- | --- | --- |
| **Diabetes** | **OR (95%CI)** | **P-Value** | **OR (95%CI)** | **P-Value** | **OR (95%CI)** | **P-Value** | **OR (95%CI)** | **P-Value** |
| **Age (ref=18 -24)** |  |  |  |  |  |  |  |  |
| **25-34** | 1.55 (1.24, 1.94) | <0.001 | 1.59 (1.1, 2.28) | 0.013 | 1.51 (1.05, 2.18) | 0.025 | 1.53 (1.06, 2.2) | 0.022 |
| **35-44** | 2.41 (1.94, 3) | <0.001 | 2.46 (1.72, 3.51) | <0.001 | 2.34 (1.64, 3.34) | <0.001 | 2.35 (1.64, 3.35) | <0.001 |
| **45-54** | 4.3 (3.47, 5.32) | <0.001 | 3.64 (2.56, 5.17) | <0.001 | 3.47 (2.44, 4.94) | <0.001 | 3.47 (2.44, 4.93) | <0.001 |
| **55-64** | 5.7 (4.6, 7.06) | <0.001 | 3.89 (2.7, 5.6) | <0.001 | 3.69 (2.56, 5.31) | <0.001 | 3.74 (2.6, 5.38) | <0.001 |
| **≥65** | 6.65 (5.36, 8.24) | <0.001 | 4.38 (2.99, 6.43) | <0.001 | 4.23 (2.89, 6.21) | <0.001 | 4.29 (2.92, 6.29) | <0.001 |
| **Male** | 1.75 (1.67, 1.84) | <0.001 | 1.91 (1.69, 2.15) | <0.001 | 1.94 (1.72, 2.19) | <0.001 | 1.95 (1.73, 2.2) | <0.001 |
| **Non-Hispanic** | 0.82 (0.75, 0.9) | <0.001 | 0.77 (0.64, 0.93) | 0.007 | 0.79 (0.66, 0.96) | 0.015 | 0.79 (0.65, 0.95) | 0.012 |
| **Caucasian** | 0.67 (0.64, 0.71) | <0.001 | 0.71 (0.62, 0.82) | <0.001 | 0.73 (0.64, 0.84) | <0.001 | 0.73 (0.63, 0.84) | <0.001 |
| **Married** | 1.06 (1.01, 1.12) | 0.019 | 1.16 (1.02, 1.33) | 0.023 | 1.17 (1.02, 1.33) | 0.020 | 1.16 (1.02, 1.32) | 0.027 |
| **Employed** | 0.73 (0.69, 0.78) | <0.001 | 0.65 (0.57, 0.75) | <0.001 | 0.64 (0.56, 0.73) | <0.001 | 0.66 (0.58, 0.76) | <0.001 |
| **Household Income (ref=<$25,000)** |  |  |  | 0.013 |  |  |  |  |
| **$25,000 to $49,999** | 0.87 (0.8, 0.95) | 0.002 | 0.72 (0.59, 0.87) | <0.001 | 0.71 (0.59, 0.87) | 0.001 | 0.73 (0.6, 0.88) | 0.001 |
| **$50,000 to $74,999** | 0.75 (0.68, 0.81) | <0.001 | 0.69 (0.56, 0.84) | <0.001 | 0.68 (0.55, 0.83) | <0.001 | 0.7 (0.57, 0.85) | 0.001 |
| **$75,000 to $99,999** | 0.68 (0.62, 0.75) | <0.001 | 0.63 (0.51, 0.79) | <0.001 | 0.62 (0.5, 0.77) | <0.001 | 0.64 (0.52, 0.8) | <0.001 |
| **≥$100,000** | 0.57 (0.53, 0.63) | <0.001 | 0.66 (0.53, 0.81) | <0.001 | 0.64 (0.52, 0.79) | <0.001 | 0.67 (0.54, 0.82) | <0.001 |
| **Migraine Screen Positive** | 1.37 (1.28, 1.46) | <0.001 |  |  |  |  |  |  |
| **Monthly Headache Days (ref=1 to 4)** |  |  |  |  |  |  |  |  |
| **5 to 9** |  |  | 1.25 (1.08, 1.45) | 0.003 |  |  | 1.2 (1.04, 1.4) | 0.014 |
| **10 to 14** |  |  | 1.52 (1.24, 1.85) | <0.001 |  |  | 1.45 (1.19, 1.78) | <0.001 |
| **15 to 20** |  |  | 1.47 (1.16, 1.85) | 0.001 |  |  | 1.39 (1.1, 1.75) | 0.006 |
| **≥21** |  |  | 2.1 (1.67, 2.64) | <0.001 |  |  | 2 (1.59, 2.51) | <0.001 |
| **Headache Pain Intensity (ref=low pain, 1 to 3)** |  |  |  |  |  |  |  |  |
| **Moderate Pain, 4 to 6** |  |  |  |  | 1.06 (0.77, 1.46) | 0.715 | 1.04 (0.76, 1.43) | 0.803 |
| **Severe Pain, 7 to 10** |  |  |  |  | 1.54 (1.13, 2.1) | 0.006 | 1.45 (1.06, 1.98) | 0.020 |

| **Additional File 7L**  **Psychiatric** | **MAST Overall Population  (N=92,586)** | | **MAST Respondents with Migraine MHD Model**  **(N=15,133)** | | **MAST Respondents with Migraine Headache Pain Intensity Model  (N=15,133)** | | **MAST Respondents with Migraine**  **MHD + Headache Pain Intensity Model**  **(N=15,133)** | |
| --- | --- | --- | --- | --- | --- | --- | --- | --- |
| **Anxiety** | **OR (95%CI)** | **P-Value** | **OR (95%CI)** | **P-Value** | **OR (95%CI)** | **P-Value** | **OR (95%CI)** | **P-Value** |
| **Age (ref=18 -24)** |  |  |  |  |  |  |  |  |
| **25-34** | 1.15 (1.05, 1.27) | 0.004 | 1.26 (1.08, 1.46) | 0.002 | 1.19 (1.03, 1.38) | 0.019 | 1.22 (1.05, 1.41) | 0.009 |
| **35-44** | 1.26 (1.14, 1.39) | <0.001 | 1.38 (1.19, 1.6) | <0.001 | 1.29 (1.11, 1.5) | 0.001 | 1.31 (1.13, 1.52) | <0.001 |
| **45-54** | 1.21 (1.1, 1.33) | <0.001 | 1.38 (1.19, 1.61) | <0.001 | 1.3 (1.12, 1.51) | 0.001 | 1.31 (1.12, 1.52) | 0.001 |
| **55-64** | 0.9 (0.82, 1) | 0.046 | 1.07 (0.9, 1.26) | 0.437 | 1 (0.85, 1.18) | 0.987 | 1.02 (0.86, 1.21) | 0.802 |
| **≥65** | 0.55 (0.49, 0.61) | <0.001 | 0.68 (0.56, 0.83) | <0.001 | 0.65 (0.54, 0.79) | <0.001 | 0.66 (0.54, 0.81) | <0.001 |
| **Male** | 0.64 (0.61, 0.66) | <0.001 | 0.78 (0.72, 0.85) | <0.001 | 0.79 (0.73, 0.85) | <0.001 | 0.79 (0.73, 0.86) | <0.001 |
| **Non-Hispanic** | 0.95 (0.89, 1.02) | 0.134 | 0.94 (0.84, 1.05) | 0.277 | 0.98 (0.87, 1.1) | 0.684 | 0.97 (0.86, 1.09) | 0.570 |
| **Caucasian** | 1.66 (1.58, 1.75) | <0.001 | 1.49 (1.36, 1.62) | <0.001 | 1.57 (1.44, 1.72) | <0.001 | 1.54 (1.41, 1.68) | <0.001 |
| **Married** | 0.73 (0.7, 0.77) | <0.001 | 0.73 (0.68, 0.79) | <0.001 | 0.73 (0.68, 0.79) | <0.001 | 0.73 (0.67, 0.78) | <0.001 |
| **Employed** | 0.78 (0.74, 0.82) | <0.001 | 0.73 (0.67, 0.8) | <0.001 | 0.71 (0.65, 0.78) | <0.001 | 0.74 (0.67, 0.8) | <0.001 |
| **Household Income (ref=<$25,000)** |  |  |  |  |  |  |  |  |
| **$25,000 to $49,999** | 0.81 (0.76, 0.87) | <0.001 | 0.81 (0.72, 0.92) | 0.001 | 0.82 (0.72, 0.92) | 0.001 | 0.83 (0.73, 0.94) | 0.003 |
| **$50,000 to $74,999** | 0.72 (0.67, 0.78) | <0.001 | 0.74 (0.65, 0.83) | <0.001 | 0.73 (0.64, 0.83) | <0.001 | 0.75 (0.66, 0.85) | <0.001 |
| **$75,000 to $99,999** | 0.69 (0.64, 0.74) | <0.001 | 0.68 (0.59, 0.78) | <0.001 | 0.67 (0.58, 0.77) | <0.001 | 0.7 (0.61, 0.8) | <0.001 |
| **≥$100,000** | 0.67 (0.62, 0.72) | <0.001 | 0.68 (0.6, 0.78) | <0.001 | 0.67 (0.59, 0.76) | <0.001 | 0.7 (0.61, 0.79) | <0.001 |
| **Migraine Screen Positive** | 3.18 (3.04, 3.32) | <0.001 |  |  |  |  |  |  |
| **Monthly Headache Days (ref=1 to 4)** |  |  |  |  |  |  |  |  |
| **5 to 9** |  |  | 1.39 (1.27, 1.52) | <0.001 |  |  | 1.33 (1.22, 1.46) | <0.001 |
| **10 to 14** |  |  | 1.58 (1.39, 1.79) | <0.001 |  |  | 1.51 (1.32, 1.71) | <0.001 |
| **15 to 20** |  |  | 2.17 (1.87, 2.51) | <0.001 |  |  | 2.05 (1.77, 2.38) | <0.001 |
| **≥21** |  |  | 2.25 (1.9, 2.66) | <0.001 |  |  | 2.13 (1.8, 2.53) | <0.001 |
| **Headache Pain Intensity (ref=low pain, 1 to 3)** |  |  |  |  |  |  |  |  |
| **Moderate Pain, 4 to 6** |  |  |  |  | 1.53 (1.24, 1.88) | <0.001 | 1.5 (1.22, 1.85) | <0.001 |
| **Severe Pain, 7 to 10** |  |  |  |  | 2.21 (1.8, 2.71) | <0.001 | 2.05 (1.67, 2.52) | <0.001 |

| **Additional File 7M**  **Psychiatric** | **MAST Overall Population  (N=92,586)** | | **MAST Respondents with Migraine MHD Model**  **(N=15,133)** | | **MAST Respondents with Migraine Headache Pain Intensity Model  (N=15,133)** | | **MAST Respondents with Migraine**  **MHD + Headache Pain Intensity Model**  **(N=15,133)** | |
| --- | --- | --- | --- | --- | --- | --- | --- | --- |
| **Depression** | **OR (95%CI)** | **P-Value** | **OR (95%CI)** | **P-Value** | **OR (95%CI)** | **P-Value** | **OR (95%CI)** | **P-Value** |
| **Age (ref=18 -24)** |  |  |  |  |  |  |  |  |
| **25-34** | 1.22 (1.1, 1.36) | <0.001 | 1.39 (1.19, 1.63) | <0.001 | 1.32 (1.13, 1.55) | <0.001 | 1.35 (1.15, 1.58) | <0.001 |
| **35-44** | 1.47 (1.32, 1.63) | <0.001 | 1.63 (1.39, 1.91) | <0.001 | 1.54 (1.31, 1.8) | <0.001 | 1.56 (1.33, 1.83) | <0.001 |
| **45-54** | 1.5 (1.35, 1.66) | <0.001 | 1.68 (1.43, 1.97) | <0.001 | 1.59 (1.35, 1.86) | <0.001 | 1.6 (1.36, 1.88) | <0.001 |
| **55-64** | 1.19 (1.07, 1.32) | 0.001 | 1.36 (1.14, 1.62) | 0.001 | 1.28 (1.07, 1.52) | 0.006 | 1.31 (1.1, 1.56) | 0.003 |
| **≥65** | 0.67 (0.6, 0.75) | <0.001 | 0.89 (0.72, 1.09) | 0.258 | 0.86 (0.7, 1.05) | 0.135 | 0.87 (0.71, 1.07) | 0.185 |
| **Male** | 0.72 (0.69, 0.75) | <0.001 | 0.95 (0.88, 1.04) | 0.287 | 0.97 (0.89, 1.05) | 0.425 | 0.98 (0.9, 1.06) | 0.592 |
| **Non-Hispanic** | 1.03 (0.95, 1.1) | 0.498 | 1.09 (0.96, 1.23) | 0.168 | 1.13 (1, 1.28) | 0.047 | 1.12 (0.99, 1.27) | 0.067 |
| **Caucasian** | 1.49 (1.41, 1.57) | <0.001 | 1.29 (1.18, 1.42) | <0.001 | 1.37 (1.25, 1.5) | <0.001 | 1.34 (1.22, 1.46) | <0.001 |
| **Married** | 0.66 (0.63, 0.69) | <0.001 | 0.68 (0.63, 0.74) | <0.001 | 0.68 (0.63, 0.74) | <0.001 | 0.67 (0.62, 0.73) | <0.001 |
| **Employed** | 0.67 (0.64, 0.71) | <0.001 | 0.64 (0.59, 0.7) | <0.001 | 0.62 (0.57, 0.68) | <0.001 | 0.64 (0.59, 0.71) | <0.001 |
| **Household Income (ref=<$25,000)** |  |  |  |  |  |  |  |  |
| **$25,000 to $49,999** | 0.72 (0.67, 0.77) | <0.001 | 0.76 (0.67, 0.86) | <0.001 | 0.76 (0.67, 0.86) | <0.001 | 0.77 (0.68, 0.87) | <0.001 |
| **$50,000 to $74,999** | 0.6 (0.56, 0.64) | <0.001 | 0.63 (0.56, 0.72) | <0.001 | 0.62 (0.55, 0.71) | <0.001 | 0.64 (0.56, 0.73) | <0.001 |
| **$75,000 to $99,999** | 0.52 (0.48, 0.56) | <0.001 | 0.51 (0.44, 0.58) | <0.001 | 0.5 (0.43, 0.57) | <0.001 | 0.52 (0.45, 0.59) | <0.001 |
| **≥$100,000** | 0.49 (0.45, 0.52) | <0.001 | 0.47 (0.41, 0.54) | <0.001 | 0.46 (0.4, 0.53) | <0.001 | 0.48 (0.42, 0.55) | <0.001 |
| **Migraine Screen Positive** | 3.18 (3.03, 3.32) | <0.001 |  |  |  |  |  |  |
| **Monthly Headache Days (ref=1 to 4)** |  |  |  |  |  |  |  |  |
| **5 to 9** |  |  | 1.36 (1.24, 1.5) | <0.001 |  |  | 1.31 (1.2, 1.44) | <0.001 |
| **10 to 14** |  |  | 1.75 (1.54, 2) | <0.001 |  |  | 1.68 (1.47, 1.92) | <0.001 |
| **15 to 20** |  |  | 2.14 (1.84, 2.49) | <0.001 |  |  | 2.03 (1.74, 2.36) | <0.001 |
| **≥21** |  |  | 2.38 (2.01, 2.83) | <0.001 |  |  | 2.26 (1.91, 2.69) | <0.001 |
| **Headache Pain Intensity (ref=low pain, 1 to 3)** |  |  |  |  |  |  |  |  |
| **Moderate Pain, 4 to 6** |  |  |  |  | 1.62 (1.3, 2.03) | <0.001 | 1.6 (1.28, 2) | <0.001 |
| **Severe Pain, 7 to 10** |  |  |  |  | 2.31 (1.85, 2.87) | <0.001 | 2.13 (1.71, 2.66) | <0.001 |

| **Additional File 7N**  **Psychiatric** | **MAST Overall Population  (N=92,586)** | | **MAST Respondents with Migraine MHD Model**  **(N=15,133)** | | **MAST Respondents with Migraine Headache Pain Intensity Model  (N=15,133)** | | **MAST Respondents with Migraine**  **MHD + Headache Pain Intensity Model**  **(N=15,133)** | |
| --- | --- | --- | --- | --- | --- | --- | --- | --- |
| **Insomnia** | **OR (95%CI)** | **P-Value** | **OR (95%CI)** | **P-Value** | **OR (95%CI)** | **P-Value** | **OR (95%CI)** | **P-Value** |
| **Age (ref=18 -24)** |  |  |  |  |  |  |  |  |
| **25-34** | 1.43 (1.24, 1.66) | <0.001 | 1.52 (1.24, 1.85) | <0.001 | 1.42 (1.16, 1.73) | 0.001 | 1.45 (1.19, 1.78) | <0.001 |
| **35-44** | 2.01 (1.74, 2.32) | <0.001 | 2.27 (1.86, 2.77) | <0.001 | 2.08 (1.71, 2.54) | <0.001 | 2.14 (1.75, 2.61) | <0.001 |
| **45-54** | 2.79 (2.42, 3.21) | <0.001 | 3.13 (2.57, 3.81) | <0.001 | 2.87 (2.36, 3.5) | <0.001 | 2.93 (2.4, 3.58) | <0.001 |
| **55-64** | 2.97 (2.57, 3.42) | <0.001 | 3.28 (2.66, 4.05) | <0.001 | 2.97 (2.41, 3.66) | <0.001 | 3.11 (2.52, 3.84) | <0.001 |
| **≥65** | 2.14 (1.85, 2.48) | <0.001 | 2.48 (1.97, 3.14) | <0.001 | 2.32 (1.84, 2.93) | <0.001 | 2.42 (1.91, 3.06) | <0.001 |
| **Male** | 0.79 (0.75, 0.83) | <0.001 | 1.02 (0.93, 1.12) | 0.673 | 1.03 (0.94, 1.13) | 0.477 | 1.05 (0.96, 1.15) | 0.311 |
| **Non-Hispanic** | 0.81 (0.75, 0.88) | <0.001 | 0.89 (0.78, 1.02) | 0.095 | 0.94 (0.82, 1.07) | 0.350 | 0.93 (0.81, 1.06) | 0.250 |
| **Caucasian** | 1.12 (1.06, 1.19) | <0.001 | 0.98 (0.89, 1.08) | 0.704 | 1.05 (0.95, 1.16) | 0.334 | 1.02 (0.92, 1.13) | 0.698 |
| **Married** | 0.74 (0.71, 0.78) | <0.001 | 0.81 (0.74, 0.89) | <0.001 | 0.81 (0.74, 0.89) | <0.001 | 0.8 (0.73, 0.88) | <0.001 |
| **Employed** | 0.74 (0.7, 0.78) | <0.001 | 0.72 (0.65, 0.79) | <0.001 | 0.7 (0.63, 0.77) | <0.001 | 0.73 (0.66, 0.8) | <0.001 |
| **Household Income (ref=<$25,000)** |  |  |  |  |  |  |  |  |
| **$25,000 to $49,999** | 0.81 (0.74, 0.88) | <0.001 | 0.77 (0.67, 0.88) | <0.001 | 0.77 (0.67, 0.88) | <0.001 | 0.79 (0.69, 0.9) | 0.001 |
| **$50,000 to $74,999** | 0.76 (0.7, 0.83) | <0.001 | 0.74 (0.64, 0.86) | <0.001 | 0.73 (0.63, 0.84) | <0.001 | 0.76 (0.66, 0.87) | <0.001 |
| **$75,000 to $99,999** | 0.75 (0.68, 0.82) | <0.001 | 0.68 (0.58, 0.8) | <0.001 | 0.66 (0.57, 0.78) | <0.001 | 0.7 (0.6, 0.82) | <0.001 |
| **≥$100,000** | 0.81 (0.75, 0.89) | <0.001 | 0.73 (0.63, 0.85) | <0.001 | 0.71 (0.61, 0.82) | <0.001 | 0.75 (0.65, 0.87) | <0.001 |
| **Migraine Screen Positive** | 3.79 (3.6, 3.98) | <0.001 |  |  |  |  |  |  |
| **Monthly Headache Days (ref=1 to 4)** |  |  |  |  |  |  |  |  |
| **5 to 9** |  |  | 1.55 (1.4, 1.71) | <0.001 |  |  | 1.47 (1.33, 1.63) | <0.001 |
| **10 to 14** |  |  | 2.11 (1.84, 2.42) | <0.001 |  |  | 2 (1.74, 2.3) | <0.001 |
| **15 to 20** |  |  | 2.23 (1.9, 2.61) | <0.001 |  |  | 2.08 (1.77, 2.43) | <0.001 |
| **≥21** |  |  | 2.63 (2.21, 3.13) | <0.001 |  |  | 2.46 (2.07, 2.94) | <0.001 |
| **Headache Pain Intensity (ref=low pain, 1 to 3)** |  |  |  |  |  |  |  |  |
| **Moderate Pain, 4 to 6** |  |  |  |  | 1.48 (1.15, 1.89) | 0.002 | 1.43 (1.12, 1.84) | 0.005 |
| **Severe Pain, 7 to 10** |  |  |  |  | 2.43 (1.9, 3.1) | <0.001 | 2.2 (1.72, 2.81) | <0.001 |

| **Additional File 7O**  **Respiratory** | **MAST Overall Population  (N=92,586)** | | **MAST Respondents with Migraine MHD Model**  **(N=15,133)** | | **MAST Respondents with Migraine  Headache Pain Intensity Model  (N=15,133)** | | **MAST Respondents with Migraine**  **MHD + Headache Pain Intensity Model**  **(N=15,133)** | |
| --- | --- | --- | --- | --- | --- | --- | --- | --- |
| **Asthma** | **OR (95%CI)** | **P-Value** | **OR (95%CI)** | **P-Value** | **OR (95%CI)** | **P-Value** | **OR (95%CI)** | **P-Value** |
| **Age (ref=18 -24)** |  |  |  |  |  |  |  |  |
| **25-34** | 0.92 (0.83, 1.03) | 0.137 | 0.85 (0.72, 1) | 0.057 | 0.83 (0.71, 0.98) | 0.026 | 0.84 (0.71, 0.99) | 0.035 |
| **35-44** | 0.8 (0.72, 0.89) | <0.001 | 0.78 (0.66, 0.92) | 0.003 | 0.76 (0.64, 0.89) | 0.001 | 0.76 (0.64, 0.9) | 0.001 |
| **45-54** | 0.68 (0.61, 0.76) | <0.001 | 0.66 (0.56, 0.79) | <0.001 | 0.64 (0.54, 0.77) | <0.001 | 0.64 (0.54, 0.76) | <0.001 |
| **55-64** | 0.69 (0.61, 0.77) | <0.001 | 0.64 (0.53, 0.78) | <0.001 | 0.62 (0.51, 0.75) | <0.001 | 0.63 (0.52, 0.76) | <0.001 |
| **≥65** | 0.6 (0.54, 0.67) | <0.001 | 0.52 (0.41, 0.65) | <0.001 | 0.51 (0.4, 0.64) | <0.001 | 0.51 (0.41, 0.64) | <0.001 |
| **Male** | 0.8 (0.76, 0.84) | <0.001 | 1.01 (0.91, 1.11) | 0.901 | 1.01 (0.92, 1.11) | 0.816 | 1.01 (0.92, 1.12) | 0.805 |
| **Non-Hispanic** | 0.85 (0.79, 0.92) | <0.001 | 0.85 (0.75, 0.97) | 0.015 | 0.87 (0.76, 0.99) | 0.031 | 0.86 (0.76, 0.98) | 0.027 |
| **Caucasian** | 0.91 (0.86, 0.96) | <0.001 | 0.89 (0.8, 0.98) | 0.017 | 0.91 (0.82, 1.01) | 0.068 | 0.9 (0.81, 0.99) | 0.036 |
| **Married** | 0.93 (0.89, 0.98) | 0.004 | 0.94 (0.86, 1.03) | 0.198 | 0.94 (0.86, 1.03) | 0.206 | 0.94 (0.85, 1.03) | 0.170 |
| **Employed** | 0.9 (0.85, 0.95) | <0.001 | 0.9 (0.81, 1) | 0.044 | 0.88 (0.79, 0.98) | 0.017 | 0.9 (0.81, 1) | 0.054 |
| **Household Income (ref=<$25,000)** |  |  |  |  |  |  |  |  |
| **$25,000 to $49,999** | 0.88 (0.81, 0.95) | 0.002 | 0.91 (0.78, 1.05) | 0.182 | 0.9 (0.78, 1.04) | 0.171 | 0.91 (0.79, 1.06) | 0.226 |
| **$50,000 to $74,999** | 0.9 (0.83, 0.98) | 0.012 | 0.82 (0.71, 0.95) | 0.010 | 0.81 (0.7, 0.95) | 0.007 | 0.83 (0.71, 0.96) | 0.015 |
| **$75,000 to $99,999** | 0.89 (0.82, 0.98) | 0.012 | 0.91 (0.78, 1.07) | 0.256 | 0.89 (0.76, 1.05) | 0.169 | 0.92 (0.78, 1.08) | 0.314 |
| **≥$100,000** | 0.9 (0.83, 0.98) | 0.017 | 0.9 (0.77, 1.05) | 0.165 | 0.88 (0.76, 1.03) | 0.107 | 0.91 (0.78, 1.06) | 0.210 |
| **Migraine Screen Positive** | 2.03 (1.93, 2.14) | <0.001 |  |  |  |  |  |  |
| **Monthly Headache Days (ref=1 to 4)** |  |  |  |  |  |  |  |  |
| **5 to 9** |  |  | 1.13 (1.01, 1.25) | 0.031 |  |  | 1.1 (0.99, 1.23) | 0.072 |
| **10 to 14** |  |  | 1.43 (1.23, 1.66) | <0.001 |  |  | 1.4 (1.2, 1.62) | <0.001 |
| **15 to 20** |  |  | 1.31 (1.1, 1.57) | 0.003 |  |  | 1.27 (1.06, 1.52) | 0.008 |
| **≥21** |  |  | 1.89 (1.57, 2.28) | <0.001 |  |  | 1.84 (1.53, 2.22) | <0.001 |
| **Headache Pain Intensity (ref=low pain, 1 to 3)** |  |  |  |  |  |  |  |  |
| **Moderate Pain, 4 to 6** |  |  |  |  | 1.01 (0.8, 1.27) | 0.926 | 1 (0.79, 1.26) | 0.991 |
| **Severe Pain, 7 to 10** |  |  |  |  | 1.25 (0.99, 1.56) | 0.058 | 1.19 (0.95, 1.5) | 0.132 |

| **Additional File 7P**  **Respiratory** | **MAST Overall Population  (N=92,586)** | | **MAST Respondents with Migraine MHD Model**  **(N=15,133)** | | **MAST Respondents with Migraine Headache Pain Intensity Model  (N=15,133)** | | **MAST Respondents with Migraine**  **MHD + Headache Pain Intensity Model**  **(N=15,133)** | |
| --- | --- | --- | --- | --- | --- | --- | --- | --- |
| **Allergies/Hay fever** | **OR (95%CI)** | **P-Value** | **OR (95%CI)** | **P-Value** | **OR (95%CI)** | **P-Value** | **OR (95%CI)** | **P-Value** |
| **Age (ref=18 -24)** |  |  |  |  |  |  |  |  |
| **25-34** | 1.05 (0.96, 1.14) | 0.280 | 1.16 (1.01, 1.34) | 0.035 | 1.13 (0.98, 1.3) | 0.082 | 1.15 (1, 1.33) | 0.051 |
| **35-44** | 1.33 (1.22, 1.45) | <0.001 | 1.55 (1.34, 1.78) | <0.001 | 1.5 (1.3, 1.73) | <0.001 | 1.52 (1.32, 1.75) | <0.001 |
| **45-54** | 1.42 (1.3, 1.55) | <0.001 | 1.59 (1.38, 1.84) | <0.001 | 1.55 (1.34, 1.79) | <0.001 | 1.56 (1.35, 1.81) | <0.001 |
| **55-64** | 1.47 (1.35, 1.61) | <0.001 | 1.54 (1.32, 1.81) | <0.001 | 1.49 (1.28, 1.75) | <0.001 | 1.53 (1.3, 1.79) | <0.001 |
| **≥65** | 1.3 (1.19, 1.42) | <0.001 | 1.48 (1.24, 1.77) | <0.001 | 1.45 (1.21, 1.73) | <0.001 | 1.47 (1.23, 1.77) | <0.001 |
| **Male** | 0.71 (0.68, 0.73) | <0.001 | 0.86 (0.79, 0.92) | <0.001 | 0.86 (0.8, 0.93) | <0.001 | 0.87 (0.8, 0.94) | <0.001 |
| **Non-Hispanic** | 0.98 (0.93, 1.04) | 0.501 | 0.96 (0.86, 1.07) | 0.474 | 0.98 (0.88, 1.09) | 0.710 | 0.97 (0.87, 1.09) | 0.628 |
| **Caucasian** | 0.95 (0.92, 0.99) | 0.011 | 0.85 (0.78, 0.92) | <0.001 | 0.88 (0.81, 0.95) | 0.002 | 0.86 (0.79, 0.93) | <0.001 |
| **Married** | 0.96 (0.93, 1) | 0.032 | 0.93 (0.87, 1) | 0.063 | 0.93 (0.87, 1) | 0.067 | 0.93 (0.86, 1) | 0.050 |
| **Employed** | 1 (0.97, 1.04) | 0.805 | 0.99 (0.91, 1.08) | 0.884 | 0.97 (0.89, 1.05) | 0.464 | 0.99 (0.91, 1.08) | 0.884 |
| **Household Income (ref=<$25,000)** |  |  |  |  |  |  |  |  |
| **$25,000 to $49,999** | 1.03 (0.97, 1.09) | 0.405 | 1.02 (0.91, 1.15) | 0.691 | 1.02 (0.9, 1.15) | 0.759 | 1.03 (0.92, 1.16) | 0.599 |
| **$50,000 to $74,999** | 1.05 (0.99, 1.12) | 0.106 | 0.96 (0.85, 1.08) | 0.507 | 0.95 (0.84, 1.07) | 0.375 | 0.97 (0.86, 1.09) | 0.596 |
| **$75,000 to $99,999** | 1.09 (1.02, 1.16) | 0.008 | 1.07 (0.94, 1.22) | 0.302 | 1.05 (0.92, 1.19) | 0.472 | 1.08 (0.95, 1.24) | 0.231 |
| **≥$100,000** | 1.13 (1.07, 1.2) | <0.001 | 0.97 (0.86, 1.1) | 0.664 | 0.95 (0.84, 1.08) | 0.448 | 0.98 (0.87, 1.12) | 0.793 |
| **Migraine Screen Positive** | 2.49 (2.39, 2.59) | <0.001 |  |  |  |  |  |  |
| **Monthly Headache Days (ref=1 to 4)** |  |  |  |  |  |  |  |  |
| **5 to 9** |  |  | 1.26 (1.16, 1.37) | <0.001 |  |  | 1.23 (1.13, 1.34) | <0.001 |
| **10 to 14** |  |  | 1.5 (1.33, 1.7) | <0.001 |  |  | 1.47 (1.3, 1.67) | <0.001 |
| **15 to 20** |  |  | 1.55 (1.34, 1.8) | <0.001 |  |  | 1.52 (1.31, 1.76) | <0.001 |
| **≥21** |  |  | 1.83 (1.55, 2.17) | <0.001 |  |  | 1.79 (1.51, 2.12) | <0.001 |
| **Headache Pain Intensity (ref=low pain, 1 to 3)** |  |  |  |  |  |  |  |  |
| **Moderate Pain, 4 to 6** |  |  |  |  | 1.45 (1.22, 1.73) | <0.001 | 1.43 (1.2, 1.71) | <0.001 |
| **Severe Pain, 7 to 10** |  |  |  |  | 1.67 (1.4, 1.99) | <0.001 | 1.58 (1.32, 1.88) | <0.001 |

| **Additional File 7Q**  **Dermatologic** | **MAST Overall Population  (N=92,586)** | | **MAST Respondents with Migraine MHD Model**  **(N=15,133)** | | **MAST Respondents with Migraine Headache Pain Intensity Model  (N=15,133)** | | **MAST Respondents with Migraine**  **MHD + Headache Pain Intensity Model**  **(N=15,133)** | |
| --- | --- | --- | --- | --- | --- | --- | --- | --- |
| **Psoriasis** | **OR (95%CI)** | **P-Value** | **OR (95%CI)** | **P-Value** | **OR (95%CI)** | **P-Value** | **OR (95%CI)** | **P-Value** |
| **Age (ref=18 -24)** |  |  |  |  |  |  |  |  |
| **25-34** | 1.07 (0.83, 1.39) | 0.608 | 1.06 (0.73, 1.53) | 0.752 | 1.02 (0.71, 1.48) | 0.906 | 1.03 (0.71, 1.49) | 0.876 |
| **35-44** | 1.29 (1, 1.66) | 0.054 | 1.25 (0.87, 1.81) | 0.225 | 1.19 (0.83, 1.72) | 0.349 | 1.2 (0.83, 1.74) | 0.320 |
| **45-54** | 1.37 (1.06, 1.76) | 0.015 | 1.3 (0.9, 1.87) | 0.167 | 1.23 (0.85, 1.78) | 0.271 | 1.24 (0.86, 1.79) | 0.250 |
| **55-64** | 1.49 (1.16, 1.93) | 0.002 | 0.95 (0.63, 1.42) | 0.788 | 0.9 (0.6, 1.36) | 0.615 | 0.92 (0.61, 1.38) | 0.679 |
| **≥65** | 1.6 (1.24, 2.08) | <0.001 | 1.04 (0.66, 1.63) | 0.881 | 1 (0.64, 1.58) | 0.994 | 1.02 (0.65, 1.61) | 0.926 |
| **Male** | 1.2 (1.11, 1.31) | <0.001 | 1.61 (1.36, 1.91) | <0.001 | 1.63 (1.38, 1.94) | <0.001 | 1.64 (1.39, 1.95) | <0.001 |
| **Non-Hispanic** | 0.97 (0.83, 1.13) | 0.663 | 0.79 (0.61, 1.01) | 0.059 | 0.81 (0.63, 1.04) | 0.104 | 0.81 (0.63, 1.04) | 0.094 |
| **Caucasian** | 1.42 (1.27, 1.58) | <0.001 | 1.17 (0.95, 1.45) | 0.134 | 1.22 (0.99, 1.5) | 0.065 | 1.2 (0.98, 1.49) | 0.083 |
| **Married** | 0.87 (0.8, 0.96) | 0.003 | 0.89 (0.74, 1.06) | 0.191 | 0.88 (0.74, 1.06) | 0.177 | 0.88 (0.73, 1.05) | 0.160 |
| **Employed** | 0.89 (0.81, 0.99) | 0.029 | 0.78 (0.64, 0.95) | 0.014 | 0.77 (0.63, 0.94) | 0.012 | 0.78 (0.64, 0.96) | 0.017 |
| **Household Income (ref=<$25,000)** |  |  |  |  |  |  |  |  |
| **$25,000 to $49,999** | 0.89 (0.75, 1.05) | 0.161 | 0.88 (0.65, 1.18) | 0.393 | 0.89 (0.66, 1.2) | 0.437 | 0.89 (0.66, 1.21) | 0.461 |
| **$50,000 to $74,999** | 1.06 (0.9, 1.25) | 0.468 | 1.06 (0.79, 1.43) | 0.685 | 1.07 (0.79, 1.44) | 0.662 | 1.08 (0.8, 1.46) | 0.601 |
| **$75,000 to $99,999** | 1.02 (0.86, 1.21) | 0.833 | 1 (0.73, 1.39) | 0.979 | 1.01 (0.73, 1.39) | 0.966 | 1.03 (0.74, 1.42) | 0.880 |
| **≥$100,000** | 1.19 (1.01, 1.39) | 0.040 | 1.23 (0.91, 1.66) | 0.186 | 1.23 (0.91, 1.67) | 0.179 | 1.25 (0.93, 1.7) | 0.145 |
| **Migraine Screen Positive** | 1.98 (1.79, 2.18) | <0.001 |  |  |  |  |  |  |
| **Monthly Headache Days (ref=1 to 4)** |  |  |  |  |  |  |  |  |
| **5 to 9** |  |  | 1.2 (0.98, 1.47) | 0.078 |  |  | 1.15 (0.94, 1.41) | 0.185 |
| **10 to 14** |  |  | 1.43 (1.08, 1.88) | 0.012 |  |  | 1.36 (1.03, 1.8) | 0.031 |
| **15 to 20** |  |  | 1.44 (1.04, 2) | 0.027 |  |  | 1.37 (0.99, 1.89) | 0.061 |
| **≥21** |  |  | 1.18 (0.8, 1.75) | 0.396 |  |  | 1.12 (0.76, 1.66) | 0.569 |
| **Headache Pain Intensity (ref=low pain, 1 to 3)** |  |  |  |  |  |  |  |  |
| **Moderate Pain, 4 to 6** |  |  |  |  | 2.16 (1.17, 4) | 0.014 | 2.14 (1.16, 3.96) | 0.015 |
| **Severe Pain, 7 to 10** |  |  |  |  | 2.89 (1.58, 5.3) | 0.001 | 2.78 (1.51, 5.11) | 0.001 |

| **Additional File 7R**  **Dermatologic** | **MAST Overall Population  (N=92,586)** | | **MAST Respondents with Migraine MHD Model**  **(N=15,133)** | | **MAST Respondents with Migraine Headache Pain Intensity Model  (N=15,133)** | | **MAST Respondents with Migraine**  **MHD + Headache Pain Intensity Model**  **(N=15,133)** | |
| --- | --- | --- | --- | --- | --- | --- | --- | --- |
| **Rosacea** | **OR (95%CI)** | **P-Value** | **OR (95%CI)** | **P-Value** | **OR (95%CI)** | **P-Value** | **OR (95%CI)** | **P-Value** |
| **Age (ref=18 -24)** |  |  |  |  |  |  |  |  |
| **25-34** | 1.01 (0.74, 1.38) | 0.955 | 0.97 (0.61, 1.53) | 0.885 | 0.98 (0.62, 1.55) | 0.936 | 0.98 (0.62, 1.56) | 0.941 |
| **35-44** | 1.88 (1.39, 2.55) | <0.001 | 1.98 (1.28, 3.06) | 0.002 | 2.02 (1.31, 3.13) | 0.002 | 2.03 (1.31, 3.13) | 0.002 |
| **45-54** | 2.74 (2.04, 3.68) | <0.001 | 2.45 (1.59, 3.77) | <0.001 | 2.52 (1.64, 3.9) | <0.001 | 2.52 (1.63, 3.88) | <0.001 |
| **55-64** | 3.44 (2.56, 4.62) | <0.001 | 3.25 (2.08, 5.07) | <0.001 | 3.37 (2.16, 5.27) | <0.001 | 3.36 (2.15, 5.25) | <0.001 |
| **≥65** | 4.11 (3.05, 5.54) | <0.001 | 3.66 (2.29, 5.86) | <0.001 | 3.75 (2.34, 6.01) | <0.001 | 3.74 (2.33, 5.99) | <0.001 |
| **Male** | 0.44 (0.41, 0.48) | <0.001 | 0.46 (0.38, 0.56) | <0.001 | 0.46 (0.38, 0.56) | <0.001 | 0.46 (0.38, 0.56) | <0.001 |
| **Non-Hispanic** | 0.91 (0.78, 1.06) | 0.234 | 0.92 (0.69, 1.21) | 0.532 | 0.9 (0.69, 1.19) | 0.476 | 0.9 (0.69, 1.19) | 0.479 |
| **Caucasian** | 3.12 (2.73, 3.57) | <0.001 | 2.53 (1.95, 3.29) | <0.001 | 2.51 (1.93, 3.26) | <0.001 | 2.51 (1.93, 3.26) | <0.001 |
| **Married** | 0.87 (0.8, 0.95) | 0.001 | 1.01 (0.84, 1.2) | 0.934 | 1.01 (0.85, 1.21) | 0.909 | 1.01 (0.84, 1.2) | 0.940 |
| **Employed** | 0.85 (0.78, 0.93) | 0.001 | 0.96 (0.8, 1.16) | 0.662 | 0.94 (0.78, 1.13) | 0.514 | 0.95 (0.79, 1.14) | 0.584 |
| **Household Income (ref=<$25,000)** |  |  |  |  |  |  |  |  |
| **$25,000 to $49,999** | 1.12 (0.94, 1.33) | 0.192 | 1.09 (0.8, 1.49) | 0.589 | 1.07 (0.79, 1.46) | 0.666 | 1.08 (0.79, 1.48) | 0.609 |
| **$50,000 to $74,999** | 1.41 (1.19, 1.66) | <0.001 | 1.29 (0.94, 1.75) | 0.112 | 1.26 (0.93, 1.72) | 0.142 | 1.28 (0.94, 1.75) | 0.119 |
| **$75,000 to $99,999** | 1.64 (1.38, 1.94) | <0.001 | 1.55 (1.13, 2.14) | 0.007 | 1.52 (1.1, 2.09) | 0.011 | 1.55 (1.12, 2.13) | 0.008 |
| **≥$100,000** | 1.82 (1.55, 2.15) | <0.001 | 1.35 (0.98, 1.85) | 0.065 | 1.31 (0.95, 1.79) | 0.098 | 1.34 (0.98, 1.84) | 0.071 |
| **Migraine Screen Positive** | 1.68 (1.53, 1.84) | <0.001 |  |  |  |  |  |  |
| **Monthly Headache Days (ref=1 to 4)** |  |  |  |  |  |  |  |  |
| **5 to 9** |  |  | 0.93 (0.76, 1.14) | 0.477 |  |  | 0.93 (0.76, 1.15) | 0.516 |
| **10 to 14** |  |  | 1.28 (0.98, 1.67) | 0.074 |  |  | 1.29 (0.99, 1.7) | 0.061 |
| **15 to 20** |  |  | 1.02 (0.73, 1.42) | 0.909 |  |  | 1.04 (0.74, 1.45) | 0.822 |
| **≥21** |  |  | 1.45 (1.03, 2.02) | 0.031 |  |  | 1.47 (1.05, 2.05) | 0.025 |
| **Headache Pain Intensity (ref=low pain, 1 to 3)** |  |  |  |  |  |  |  |  |
| **Moderate Pain, 4 to 6** |  |  |  |  | 1.57 (1, 2.48) | 0.051 | 1.57 (1, 2.49) | 0.051 |
| **Severe Pain, 7 to 10** |  |  |  |  | 1.33 (0.85, 2.09) | 0.216 | 1.32 (0.84, 2.07) | 0.236 |

| **Additional File 7S**  **Pain** | **MAST Overall Population  (N=92,586)** | | **MAST Respondents with Migraine MHD Model**  **(N=15,133)** | | **MAST Respondents with Migraine Headache Pain Intensity Model  (N=15,133)** | | **MAST Respondents with Migraine**  **MHD + Headache Pain Intensity Model**  **(N=15,133)** | |
| --- | --- | --- | --- | --- | --- | --- | --- | --- |
| **Arthritis (Type not known)** | **OR (95%CI)** | **P-Value** | **OR (95%CI)** | **P-Value** | **OR (95%CI)** | **P-Value** | **OR (95%CI)** | **P-Value** |
| **Age (ref=18 -24)** |  |  |  |  |  |  |  |  |
| **25-34** | 1.53 (1.16, 2.01) | 0.003 | 1.69 (1.14, 2.5) | 0.009 | 1.63 (1.1, 2.42) | 0.014 | 1.65 (1.12, 2.45) | 0.012 |
| **35-44** | 2.7 (2.06, 3.53) | <0.001 | 3.09 (2.11, 4.52) | <0.001 | 2.98 (2.04, 4.37) | <0.001 | 3 (2.05, 4.39) | <0.001 |
| **45-54** | 5.2 (3.99, 6.77) | <0.001 | 5.49 (3.77, 8) | <0.001 | 5.33 (3.66, 7.77) | <0.001 | 5.33 (3.66, 7.76) | <0.001 |
| **55-64** | 8.26 (6.35, 10.75) | <0.001 | 7.76 (5.3, 11.38) | <0.001 | 7.46 (5.09, 10.93) | <0.001 | 7.57 (5.16, 11.11) | <0.001 |
| **≥65** | 11.56 (8.88, 15.07) | <0.001 | 8.38 (5.62, 12.48) | <0.001 | 8.14 (5.47, 12.13) | <0.001 | 8.28 (5.55, 12.34) | <0.001 |
| **Male** | 1.11 (1.06, 1.17) | <0.001 | 1.31 (1.17, 1.47) | <0.001 | 1.33 (1.18, 1.49) | <0.001 | 1.33 (1.19, 1.5) | <0.001 |
| **Non-Hispanic** | 1.04 (0.94, 1.16) | 0.455 | 0.91 (0.75, 1.1) | 0.326 | 0.93 (0.77, 1.12) | 0.447 | 0.92 (0.76, 1.11) | 0.405 |
| **Caucasian** | 1 (0.94, 1.07) | 0.956 | 0.85 (0.75, 0.98) | 0.024 | 0.88 (0.77, 1.01) | 0.060 | 0.87 (0.76, 1) | 0.045 |
| **Married** | 1.12 (1.06, 1.18) | <0.001 | 1.12 (0.99, 1.27) | 0.070 | 1.13 (1, 1.28) | 0.053 | 1.12 (0.99, 1.27) | 0.075 |
| **Employed** | 0.81 (0.77, 0.86) | <0.001 | 0.79 (0.7, 0.9) | <0.001 | 0.77 (0.68, 0.88) | <0.001 | 0.8 (0.7, 0.91) | 0.001 |
| **Household Income (ref=<$25,000)** |  |  |  |  |  |  |  |  |
| **$25,000 to $49,999** | 0.98 (0.89, 1.07) | 0.632 | 0.91 (0.75, 1.1) | 0.327 | 0.9 (0.74, 1.08) | 0.248 | 0.92 (0.76, 1.11) | 0.383 |
| **$50,000 to $74,999** | 0.81 (0.73, 0.89) | <0.001 | 0.72 (0.59, 0.88) | 0.001 | 0.71 (0.58, 0.86) | 0.001 | 0.73 (0.6, 0.89) | 0.002 |
| **$75,000 to $99,999** | 0.78 (0.7, 0.86) | <0.001 | 0.74 (0.6, 0.91) | 0.005 | 0.71 (0.58, 0.88) | 0.002 | 0.75 (0.61, 0.93) | 0.008 |
| **≥$100,000** | 0.71 (0.65, 0.79) | <0.001 | 0.71 (0.58, 0.87) | 0.001 | 0.68 (0.56, 0.83) | <0.001 | 0.72 (0.59, 0.88) | 0.002 |
| **Migraine Screen Positive** | 2.2 (2.07, 2.35) | <0.001 |  |  |  |  |  |  |
| **Monthly Headache Days (ref=1 to 4)** |  |  |  |  |  |  |  |  |
| **5 to 9** |  |  | 1.17 (1.02, 1.35) | 0.026 |  |  | 1.15 (0.99, 1.32) | 0.061 |
| **10 to 14** |  |  | 1.75 (1.46, 2.1) | <0.001 |  |  | 1.7 (1.42, 2.05) | <0.001 |
| **15 to 20** |  |  | 1.4 (1.12, 1.74) | 0.003 |  |  | 1.35 (1.08, 1.69) | 0.007 |
| **≥21** |  |  | 2.55 (2.07, 3.15) | <0.001 |  |  | 2.48 (2.01, 3.05) | <0.001 |
| **Headache Pain Intensity (ref=low pain, 1 to 3)** |  |  |  |  |  |  |  |  |
| **Moderate Pain, 4 to 6** |  |  |  |  | 1.22 (0.9, 1.64) | 0.202 | 1.2 (0.88, 1.62) | 0.246 |
| **Severe Pain, 7 to 10** |  |  |  |  | 1.55 (1.15, 2.08) | 0.004 | 1.45 (1.08, 1.96) | 0.014 |

| **Additional File 7T**  **Pain** | **MAST Overall Population  (N=92,586)** | | **MAST Respondents with Migraine MHD Model**  **(N=15,133)** | | **MAST Respondents with Migraine Headache Pain Intensity Model  (N=15,133)** | | **MAST Respondents with Migraine**  **MHD + Headache Pain Intensity Model**  **(N=15,133)** | |
| --- | --- | --- | --- | --- | --- | --- | --- | --- |
| **Osteoarthritis** | **OR (95%CI)** | **P-Value** | **OR (95%CI)** | **P-Value** | **OR (95%CI)** | **P-Value** | **OR (95%CI)** | **P-Value** |
| **Age (ref=18 -24)** |  |  |  |  |  |  |  |  |
| **25-34** | 1.94 (1.25, 3.02) | 0.003 | 3.73 (1.71, 8.16) | 0.001 | 3.66 (1.67, 7.98) | 0.001 | 3.7 (1.7, 8.09) | 0.001 |
| **35-44** | 5.67 (3.71, 8.65) | <0.001 | 10.5 (4.91, 22.45) | <0.001 | 10.24 (4.79, 21.9) | <0.001 | 10.35 (4.84, 22.14) | <0.001 |
| **45-54** | 14.95 (9.86, 22.65) | <0.001 | 28.29 (13.32, 60.06) | <0.001 | 27.7 (13.04, 58.82) | <0.001 | 27.83 (13.1, 59.12) | <0.001 |
| **55-64** | 31.77 (20.98, 48.1) | <0.001 | 50.53 (23.74, 107.57) | <0.001 | 48.85 (22.95, 103.99) | <0.001 | 49.99 (23.47, 106.44) | <0.001 |
| **≥65** | 45.87 (30.27, 69.5) | <0.001 | 69.9 (32.63, 149.71) | <0.001 | 68.13 (31.81, 145.91) | <0.001 | 69.52 (32.45, 148.92) | <0.001 |
| **Male** | 0.45 (0.43, 0.47) | <0.001 | 0.55 (0.48, 0.63) | <0.001 | 0.55 (0.48, 0.63) | <0.001 | 0.55 (0.48, 0.63) | <0.001 |
| **Non-Hispanic** | 1.24 (1.1, 1.4) | 0.001 | 0.99 (0.8, 1.23) | 0.942 | 1.01 (0.81, 1.26) | 0.915 | 1 (0.8, 1.24) | 0.995 |
| **Caucasian** | 1.3 (1.22, 1.39) | <0.001 | 1.15 (0.98, 1.34) | 0.081 | 1.17 (1, 1.37) | 0.051 | 1.16 (0.99, 1.36) | 0.062 |
| **Married** | 0.87 (0.82, 0.92) | <0.001 | 0.91 (0.8, 1.04) | 0.162 | 0.91 (0.8, 1.04) | 0.158 | 0.91 (0.8, 1.04) | 0.159 |
| **Employed** | 0.66 (0.62, 0.7) | <0.001 | 0.59 (0.52, 0.67) | <0.001 | 0.58 (0.51, 0.66) | <0.001 | 0.59 (0.52, 0.67) | <0.001 |
| **Household Income (ref=<$25,000)** |  |  |  |  |  |  |  |  |
| **$25,000 to $49,999** | 0.9 (0.82, 0.98) | 0.022 | 0.74 (0.61, 0.9) | 0.003 | 0.74 (0.61, 0.9) | 0.002 | 0.75 (0.61, 0.91) | 0.003 |
| **$50,000 to $74,999** | 0.87 (0.79, 0.96) | 0.004 | 0.73 (0.6, 0.89) | 0.002 | 0.72 (0.59, 0.88) | 0.001 | 0.73 (0.6, 0.9) | 0.003 |
| **$75,000 to $99,999** | 0.85 (0.77, 0.94) | 0.002 | 0.66 (0.53, 0.83) | <0.001 | 0.65 (0.52, 0.81) | <0.001 | 0.67 (0.53, 0.83) | <0.001 |
| **≥$100,000** | 0.89 (0.81, 0.98) | 0.014 | 0.68 (0.55, 0.84) | <0.001 | 0.66 (0.53, 0.81) | <0.001 | 0.68 (0.55, 0.84) | <0.001 |
| **Migraine Screen Positive** | 1.88 (1.76, 2.01) | <0.001 |  |  |  |  |  |  |
| **Monthly Headache Days (ref=1 to 4)** |  |  |  |  |  |  |  |  |
| **5 to 9** |  |  | 1.35 (1.17, 1.56) | <0.001 |  |  | 1.33 (1.15, 1.54) | <0.001 |
| **10 to 14** |  |  | 1.22 (0.99, 1.51) | 0.064 |  |  | 1.21 (0.98, 1.49) | 0.083 |
| **15 to 20** |  |  | 1.63 (1.31, 2.02) | <0.001 |  |  | 1.6 (1.28, 1.99) | <0.001 |
| **≥21** |  |  | 1.49 (1.17, 1.91) | 0.001 |  |  | 1.47 (1.15, 1.88) | 0.002 |
| **Headache Pain Intensity (ref=low pain, 1 to 3)** |  |  |  |  |  |  |  |  |
| **Moderate Pain, 4 to 6** |  |  |  |  | 1.3 (0.95, 1.78) | 0.105 | 1.26 (0.92, 1.73) | 0.152 |
| **Severe Pain, 7 to 10** |  |  |  |  | 1.44 (1.06, 1.97) | 0.020 | 1.35 (0.99, 1.85) | 0.057 |

| **Additional File 7U**  **Pain** | **MAST Overall Population  (N=92,586)** | | **MAST Respondents with Migraine MHD Model**  **(N=15,133)** | | **MAST Respondents with Migraine Headache Pain Intensity Model  (N=15,133)** | | **MAST Respondents with Migraine**  **MHD + Headache Pain Intensity Model**  **(N=15,133)** | |
| --- | --- | --- | --- | --- | --- | --- | --- | --- |
| **Rheumatoid Arthritis** | **OR (95%CI)** | **P-Value** | **OR (95%CI)** | **P-Value** | **OR (95%CI)** | **P-Value** | **OR (95%CI)** | **P-Value** |
| **Age (ref=18 -24)** |  |  |  |  |  |  |  |  |
| **25-34** | 1.63 (1.13, 2.34) | 0.008 | 1.94 (1.11, 3.38) | 0.020 | 1.79 (1.03, 3.12) | 0.040 | 1.81 (1.04, 3.16) | 0.037 |
| **35-44** | 2.23 (1.56, 3.19) | <0.001 | 2.64 (1.53, 4.57) | 0.001 | 2.39 (1.38, 4.13) | 0.002 | 2.41 (1.39, 4.17) | 0.002 |
| **45-54** | 3.2 (2.25, 4.54) | <0.001 | 3.78 (2.2, 6.51) | <0.001 | 3.43 (1.99, 5.9) | <0.001 | 3.43 (1.99, 5.91) | <0.001 |
| **55-64** | 3.65 (2.56, 5.19) | <0.001 | 3.41 (1.94, 6) | <0.001 | 3.07 (1.74, 5.41) | <0.001 | 3.13 (1.78, 5.51) | <0.001 |
| **≥65** | 3.63 (2.54, 5.19) | <0.001 | 3.59 (1.97, 6.53) | <0.001 | 3.34 (1.84, 6.09) | <0.001 | 3.42 (1.88, 6.23) | <0.001 |
| **Male** | 0.9 (0.82, 0.99) | 0.032 | 1.3 (1.07, 1.57) | 0.007 | 1.33 (1.1, 1.61) | 0.003 | 1.34 (1.11, 1.62) | 0.003 |
| **Non-Hispanic** | 0.64 (0.55, 0.75) | <0.001 | 0.81 (0.61, 1.07) | 0.136 | 0.85 (0.64, 1.13) | 0.258 | 0.84 (0.64, 1.11) | 0.226 |
| **Caucasian** | 0.83 (0.75, 0.93) | 0.001 | 0.77 (0.63, 0.95) | 0.015 | 0.82 (0.67, 1.01) | 0.064 | 0.81 (0.66, 1) | 0.047 |
| **Married** | 1.12 (1.01, 1.25) | 0.025 | 1.02 (0.84, 1.24) | 0.841 | 1.02 (0.84, 1.24) | 0.856 | 1.01 (0.83, 1.23) | 0.911 |
| **Employed** | 0.75 (0.67, 0.83) | <0.001 | 0.75 (0.61, 0.92) | 0.007 | 0.74 (0.6, 0.91) | 0.005 | 0.77 (0.62, 0.94) | 0.013 |
| **Household Income (ref=<$25,000)** |  |  |  |  |  |  |  |  |
| **$25,000 to $49,999** | 0.79 (0.67, 0.92) | 0.004 | 0.7 (0.52, 0.94) | 0.019 | 0.71 (0.53, 0.95) | 0.023 | 0.72 (0.54, 0.97) | 0.032 |
| **$50,000 to $74,999** | 0.67 (0.57, 0.79) | <0.001 | 0.72 (0.53, 0.98) | 0.035 | 0.71 (0.53, 0.97) | 0.032 | 0.74 (0.54, 1.01) | 0.054 |
| **$75,000 to $99,999** | 0.72 (0.61, 0.86) | <0.001 | 0.84 (0.61, 1.16) | 0.300 | 0.84 (0.61, 1.15) | 0.277 | 0.87 (0.63, 1.21) | 0.416 |
| **≥$100,000** | 0.67 (0.57, 0.79) | <0.001 | 0.72 (0.52, 0.99) | 0.043 | 0.71 (0.52, 0.97) | 0.033 | 0.74 (0.54, 1.02) | 0.068 |
| **Migraine Screen Positive** | 2.11 (1.89, 2.35) | <0.001 |  |  |  |  |  |  |
| **Monthly Headache Days (ref=1 to 4)** |  |  |  |  |  |  |  |  |
| **5 to 9** |  |  | 1.36 (1.09, 1.7) | 0.007 |  |  | 1.27 (1.02, 1.59) | 0.035 |
| **10 to 14** |  |  | 1.81 (1.36, 2.41) | <0.001 |  |  | 1.67 (1.25, 2.23) | <0.001 |
| **15 to 20** |  |  | 1.84 (1.33, 2.54) | <0.001 |  |  | 1.66 (1.2, 2.3) | 0.002 |
| **≥21** |  |  | 1.89 (1.33, 2.7) | <0.001 |  |  | 1.73 (1.21, 2.46) | 0.003 |
| **Headache Pain Intensity (ref=low pain, 1 to 3)** |  |  |  |  |  |  |  |  |
| **Moderate Pain, 4 to 6** |  |  |  |  | 1.01 (0.58, 1.78) | 0.960 | 0.99 (0.56, 1.74) | 0.971 |
| **Severe Pain, 7 to 10** |  |  |  |  | 2.11 (1.22, 3.63) | 0.007 | 1.95 (1.13, 3.36) | 0.016 |
